# The role of Arabidopsis *Splicing Factor 30* in floral transition and the implications of its alternative splicing

**DOI:** 10.1093/plphys/kiaf335

**Published:** 2025-07-31

**Authors:** Di Zhang, Min Li, Mehtab Muhammad Aslam, Mingkun Huang, Mo-Xian Chen, Ying-Gao Liu, Jianhua Zhang

**Affiliations:** State Key Laboratory of Green Pesticide, Ministry of Education, Center for R&D of Fine Chemicals of Guizhou University, Guiyang 550000, China; State Key Laboratory of Agrobiotechnology, The Chinese University of Hong Kong, Shatin, Hong Kong; Department of Biology, Hong Kong Baptist University, Kowloon Tong, Hong Kong; State Key Laboratory of Crop Biology, College of Life Science, Shandong Agricultural University, Taian 271000, China; State Key Laboratory of Agrobiotechnology, The Chinese University of Hong Kong, Shatin, Hong Kong; State Key Laboratory of Agrobiotechnology, The Chinese University of Hong Kong, Shatin, Hong Kong; Lushan Botanical Garden, Chinese Academy of Sciences, Jiujiang, Jiangxi 332900, China; State Key Laboratory of Green Pesticide, Ministry of Education, Center for R&D of Fine Chemicals of Guizhou University, Guiyang 550000, China; State Key Laboratory of Crop Biology, College of Life Science, Shandong Agricultural University, Taian 271000, China; State Key Laboratory of Agrobiotechnology, The Chinese University of Hong Kong, Shatin, Hong Kong; Department of Biology, Hong Kong Baptist University, Kowloon Tong, Hong Kong

## Abstract

Splicing factor 30 (SPF30) is a pivotal spliceosomal protein in human pre-mRNA splicing; however, its function in plants remains unclear. Previously, we identified the *SPF30* genes throughout the plant kingdom and found that they have a conserved second intron that undergoes frequent alternative splicing (AS). In this study, we characterized *SPF30* and its various alternative isoforms in Arabidopsis (*Arabidopsis thaliana*). Loss-of-function mutation in *SPF30* caused early flowering and impaired expression and splicing of the floral repressor *FLOWERING LOCUS C* (*FLC*). Subsequent genetic and molecular analyses further suggested that *SPF30* may regulate floral transition mostly through *FLC*. The primary transcript, *SPF30.1*, encodes a functional splicing factor associated with spliceosomal core proteins, while isoforms retaining a partial fragment of the second intron are subjected to nonsense-mediated mRNA decay (NMD). Moreover, a long, NMD-immune isoform with the entire second intron retained can be further processed to either *SPF30.1* or NMD-sensitive isoforms, potentially enabling the fine-tuning of *SPF30* expression post-transcriptionally. Analysis of the addition and deletion of the second intron further indicated that it negatively controls *SPF30* function. Our results highlight the critical role of SPF30 as a plant splicing factor involved in floral transition and propose a mechanism for the regulation of *SPF30* itself via AS.

## Teaser

A plant splicing factor influences flowering time and its activity is regulated by how its own mRNA is spliced.

## Introduction

Eukaryotic genomes are known for containing introns. In higher eukaryotes, intron removal is a flexible process, and a single pre-mRNA can be spliced into different mature mRNAs through various alternative splicing (AS) patterns, including alternative 5′ splice site, alternative 3′ splice site, exon skipping and intron retention. AS is a major post-transcriptional regulation mode and can influence gene expression at different levels. First, transcript isoforms generated by AS have the potential to encode protein isoforms with different domains or structures, thereby expanding the range of proteins a genome can produce (Yu et al. 2016; Liu et al. 2017). Additionally, some transcript isoforms carry signals such as a premature termination codon (PTC) and undergo degradation through the nonsense-mediated mRNA decay (NMD) pathway, which enables negative regulation of mRNA levels by a mechanism known as AS-coupled NMD (AS-NMD) (Lewis et al. 2003; McGlincy and Smith 2008; Shaul 2015). AS is ubiquitous among higher eukaryotes. Estimate suggests that more than 95% and 61% of the multiexonic genes undergo AS in human (Wang et al. 2008; Pan et al. 2009) and in Arabidopsis (*Arabidopsis thaliana*; Marquez et al. 2012; Zhang et al. 2022), respectively. In plants, a number of genes have been reported to have alternative isoforms with diverse physiological roles (Tognacca et al. 2023). However, despite the extensive identification of splice isoforms, only a small fraction of them has been experimentally studied, and there is ongoing debate over whether most of the alternative isoforms are functionally important (McGlincy and Smith 2008; Blencowe 2017; Tress et al. 2017).

Spliceosome is the cellular machinery that cuts out introns from the newly synthesized pre-mRNAs. The major type of the spliceosome is made of five uridine-rich small nuclear ribonucleoprotein particles (U snRNPs), U1, U2, U5, and U4/6, along with numerous protein factors (Will and Luhrmann 2011). During splicing, these components assemble onto the intron in a specific order (Matera and Wang 2014; Wilkinson et al. 2020). U1 and U2 are the first two that bind to the pre-mRNA and play crucial roles in initial recognition of the introns. Following that, a preassembled U4/U6.U5 tri-snRNP is recruited, and the immature spliceosome undergoes confirmational rearrangements to become enzymatically active. In higher eukaryotes, pre-mRNA splicing is elaborately regulated by various cis-elements and regulator proteins, including serine/arginine-rich (SR) proteins and heterogeneous nuclear ribonucleoprotein particle (hnRNP) proteins (Kornblihtt et al. 2013; Zhang et al. 2020). While the spliceosome has been studied extensively in animals and yeast, we know very little about it in plants. Moreover, plants have a higher number of splicing factors than humans, and their genes frequently undergoes AS, which further complicates the regulation of AS in plants (Reddy and Shad Ali 2011; Barta et al. 2012).

Human splicing factor 30 (SPF30) is a U2-related spliceosomal protein with a molecular weight of about 30 kilo-Dalton (Neubauer et al. 1998; Talbot et al. 1998). It plays a crucial role in spliceosomal assembly by recruiting the preassembled U4/U6.5 tri-snRNP to the prespliceosome made mainly of U1 and U2 (Meister et al. 2001; Rappsilber et al. 2001; Little and Jurica 2008). Specifically, SPF30 can simultaneously interact with U2 snRNP auxiliary factor (U2AF) and a tri-snRNP member, human pre-mRNA processing factor 3 (hPrp3), bringing these two parts together (Little and Jurica 2008). In HeLa nuclear extracts, deficiency in SPF30 prevented the formation of the mature spliceosome (Meister et al. 2001). Unlike its putative paralogue survival of motor neuron 1 (SMN1), which aids snRNP biogenesis and is linked to the development spinal muscular atrophy (Buhler et al. 1999), SPF30 is primarily known as a splicing factor in both human and yeast (Rappsilber et al. 2001; Bernard et al. 2010). Although putative orthologues of human *SPF30* have been found in plants (Barta et al. 2012), little is known about their functions and involvement in pre-mRNA splicing. In a previous study, we identified the *SPF30* genes in many plant species and found that they are conserved throughout the plant kingdom, suggesting that they might play a fundamental role in plant physiology (Zhang et al. 2019). Intriguingly, we also noticed that the *SPF30* genes frequently undergo AS and have evolutionarily conserved AS sites within their second intron (Zhang et al. 2019). Although having multiple AS events is common among splicing-related genes (Reddy and Shad Ali 2011), the biological meaning of the existence of various splice isoforms and their functional implications remain poorly understood.

Therefore, in this study, we aimed to functionally characterize the *SPF30* gene as well as its AS events in Arabidopsis. To explore the roles of individual *SPF30* isoforms, we systematically examined their properties at both the RNA and protein levels. Our findings suggest that *SPF30* is required for floral transition and *FLOWERING LOCUS C* (*FLC*) expression. While *SPF30.1* encodes a functional splicing factor, other isoforms likely play distinct regulatory roles at the RNA level. Based on these results, we propose a mechanism illustrating how *SPF30* itself is elaborately regulated via AS.

## Results

### *SPF30* has multiple transcripts isoforms

**Figure 1.**
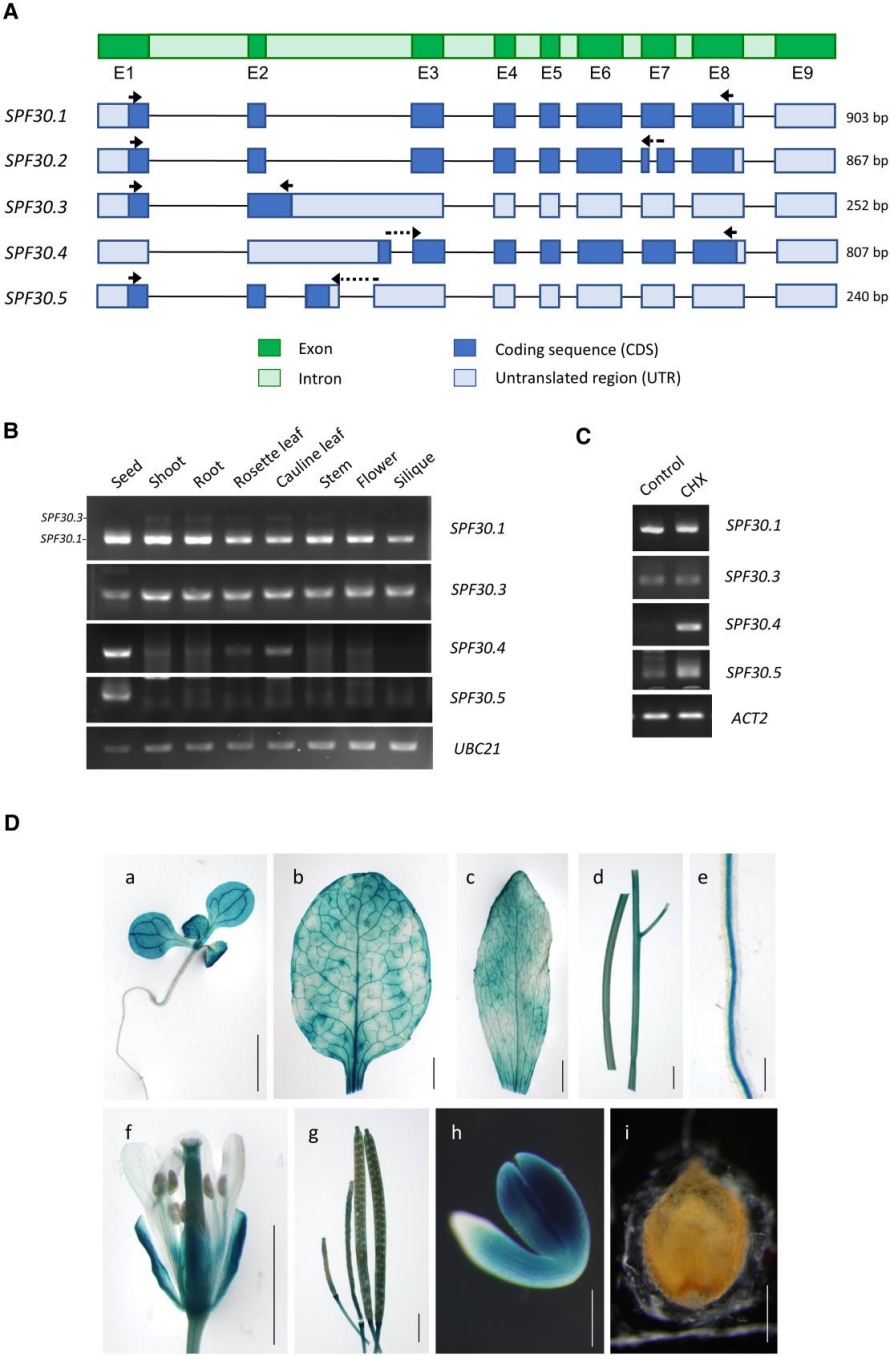
Expression pattern of *SPF30* transcript isoforms in Arabidopsis tissues. **A)** Schematic view of the *SPF30* genomic structure (top) and its five alternative isoforms (below). The length of each coding sequence (CDS) is indicated on the right. Arrows indicate the positions of primers used for detection of each isoform in B and C. **B)** RT-PCR analysis of *SPF30* transcript isoforms in different Arabidopsis tissues. All isoforms except *SPF30.2* were detected, and the bands were verified by Sanger sequencing. The upper band detected by the primers for *SPF30.1* was *SPF30.3*. **C)** RT-PCR analysis of *SPF30* transcript isoforms in 12-d-old seedlings treated with 100 *μ*g/ml cycloheximide (CHX) or water (control) for 3 h at room temperature. **D)** Expression pattern of *SPF30pro::GUS* in transgenic Arabidopsis. a: 10-d-old seedling; b: 4-wk-old rosette leaf; c: 6-wk-old cauline leaf; d: stems; e: root; f: flower; g: siliques; h: mature embryo; i: seed coat of mature embryo. Scale bar = 2 mm (a, b, c, d, f, and g) and 0.2 mm (e, h, and i).

Arabidopsis *SPF30* (AT2G02570) exhibits multiple AS isoforms, with some identified in our previous third-generation RNA-sequencing study (Supplementary Fig. S1) (Zhu et al. 2017). We selected five representative ones, *SPF30.1* to *SPF30.5*, which exhibit variations in their coding sequences (CDSs; Fig. 1A and Supplementary Data Set 1).

**Figure 2.**
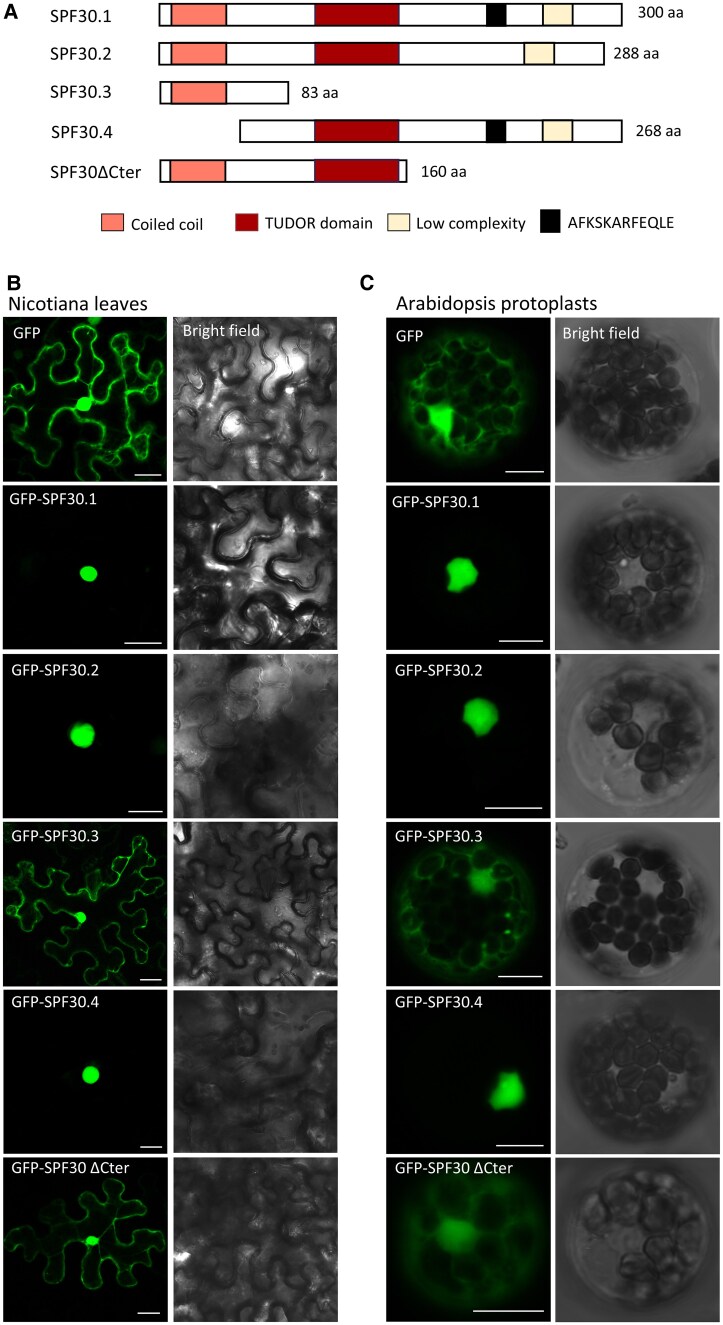
Subcellular localization of GFP-SPF30.1 and its protein variants. **A)** Schematic view of the deduced SPF30 protein isoforms and a truncated mutant lacking the C terminus (SPF30ΔCter). The number of amino acids is indicated to the right of each structure. Domains and motifs were predicted by the SMART program (http://smart.embl-heidelberg.de). A black square indicates a 12-amino acid fragment missing in SPF30.2 due to alternative splicing. **B** and **C)** Subcellular localization of SPF30 protein isoforms and SPF30ΔCter in plant cells. GFP-tagged SPF30 protein variants were transiently expressed in Nicotiana leaf epidermal cells (B) or in Arabidopsis protoplasts (C), and cells were microscopically visualized 48 h post transformation. Bars = 25 *μ*m (B) and 10 *μ*m (C).

*SPF30.1* encodes the full-length protein of 300 amino acids, containing a central TUDOR domain, a coiled coil in its N terminus, and a low-complexity region at its C-terminal end (Fig. 2A). *SPF30.2* has a 36-nt cryptic intron located in its seventh exon, resulting in a predicted protein lacking 12 amino acids. In *SPF30.3*, the second intron is fully retained, which creates a premature termination codon (PTC) and the predicted protein is severely truncated. *SPF30.4* and *SPF30.5* also undergo AS in their second intron and have premature termination like *SPF30.3* (Supplementary Figure S1). Interestingly, *SPF30.4* also contains a downstream AUG that hypothetically encodes an in-frame protein product. Recent studies have shown that alternative translation start sites, such as unannotated AUGs, are prevalent in plants (Li and Liu 2020; Wu et al. 2024). Notably, *SPF30.4* is the only *SPF30* isoform with a long downstream CDS, which results from an AS pattern unique to this isoform. Since the regular CDS in *SPF30.4* is identical to that in *SPF30.3*, this downstream CDS was used as the representative CDS of *SPF30.4* in our analysis.

The distinct compositions of these mRNA isoforms suggest that they may have different molecular properties which we aimed to investigate in this study. Additionally, the CDSs of these alternative isoforms could potentially produce truncated proteins lacking specific domains. Here we were also curious to explore the effects of these missing domains on the biological functions of the full-length SPF30.1 protein.

### The expression pattern of the *SPF30* gene and its transcript isoforms in Arabidopsis tissues and under CHX treatment

We analyzed the expression of each *SPF30* isoform in different Arabidopsis tissues by reverse transcription-PCR (RT-PCR; Fig. 1B) and reverse transcription-quantitative PCR (RT-qPCR; Supplementary Fig. S2B). The RT-PCR primers designed for *SPF30.1* could potentially detect all isoforms (Fig. 1A), but the predominant gel band corresponded to *SPF30.1*, with a faint upper band corresponding to *SPF30.3,* suggesting that *SPF30.1* is the primary product of the gene (Fig. 1B), while other isoforms exist in much lower amounts. *SPF30.1* was detected in all tested tissues, indicating that the gene was constitutively expressed. Interestingly, *SPF30.3* was less detected in seeds than in other tissues, while *SPF30.4* and *SPF30.5* were mainly distributed in seeds, with *SPF30.5* barely detectable in other tissues (Fig. 1B and Supplementary Fig. S2B). The *SPF30* bands amplified in Fig. 1B were verified by Sanger sequencing, which confirms the natural existence of these isoforms. However, we were unable to detect *SPF30.2* even with extended PCR cycles (Supplementary Fig. S3). This suggests that this isoform may not be expressed or may exist at very low levels under normal conditions.

We tested the sensitivity of individual *SPF30* transcript isoforms to NMD by treating Arabidopsis seedlings with cycloheximide (CHX), a commonly used NMD inhibitor (Palusa and Reddy 2010). Upon CHX treatment, the abundance of NMD-sensitive transcripts is expected to increase. Our RT-PCR (Fig. 1C) and RT-qPCR (Supplementary Fig. S2C) results show that the mRNA levels of *SPF30.4* and *SPF30.5* increased significantly upon CHX treatment, suggesting that these two isoforms are NMD targets. Notably, these isoforms were still detectable without NMD inhibition, indicating that they are not completely degraded. By contrast, *SPF30.3*, despite carrying a PTC, showed stable levels after the treatment, suggesting that it is immune to NMD. Moreover, *SPF30.2* remained undetected even after CHX treatment (Supplementary Fig. S3), suggesting that its absence is not due to NMD degradation.

To investigate the spatio-temporal expression of the *SPF30* gene, we generated transgenic Arabidopsis lines expressing a *SPF30pro::GUS* construct. Different tissues harvested from these lines were analyzed by histochemical staining. GUS expression was detected in various plant parts, including seedlings, leaves, stems, roots, flowers, siliques and embryos (Fig. 1D). Our results suggest that the *SPF30* promoter drives constitutive gene expression in different tissues and developmental stages.

### Subcellular localization of SPF30 protein variants

We then analyzed the subcellular localization of SPF30.1 and its protein variants. The analysis of various hypothetical proteins predicted by the CDSs of *SPF30* isoforms not only provides information on the potential cellular effects of these isoforms in case they are translated but also serve as effective deletion mutants for revealing the roles of specific SPF30 protein domains. An SPF30 variant lacking the entire C terminus (SPF30ΔCter; Fig. 2A and Supplementary Data Set 1) was also included for analysis.

As shown in Fig. 2, B and C, SPF30.1 localized exclusively to the cell nucleus in both Arabidopsis protoplasts and Nicotiana (*Nicotiana benthamiana*) leaves, which is consistent with its expected role as a splicing factor. SPF30.2 and SPF30.4 exhibited similar localization patterns as SPF30.1. In contrast, SPF30.3 and SPF30ΔCter showed diffused localization throughout the cell, resembling the pattern of the GFP control, suggesting that the C terminus is essential for the exclusive nuclear distribution of SPF30.

### SPF30.1 co-localized with putative spliceosomal proteins in subnuclear domains

**Figure 3.**
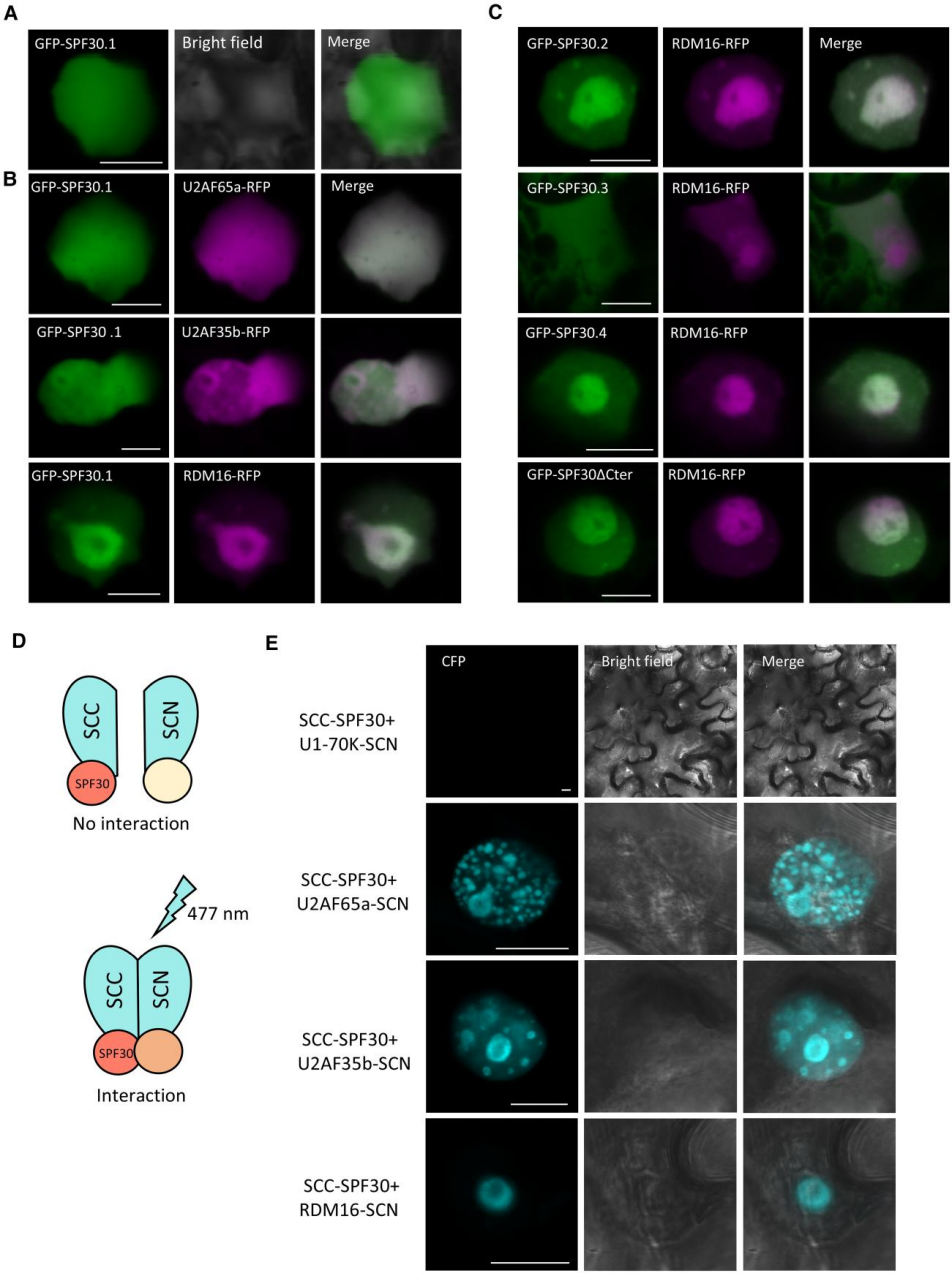
Co-localization and interaction of SPF30.1 with U2AF65a, U2AF35b and RDM16 in subnuclear domains. **A** and **B)** GFP-tagged SPF30.1 was expressed alone (A) or co-expressed with RFP-tagged U2AF65a, U2AF35b and RDM16 in Arabidopsis protoplasts (B). White in the merged images indicates co-localization. **C)** SPF30 protein variants were co-expressed with RDM16 in Arabidopsis protoplasts. **D)** Schematic illustration of the bimolecular fluorescence complementation (BIFC) assay. SCC: SCFP3A C terminus; SCN: SCFP3A N terminus. **E)** BIFC assay of SPF30.1 with U2AF65a, U2AF35b and RDM16 in Nicotiana leaf cells. Complementation of cyan fluorescence (477 nm) indicates interaction between the two proteins tagged with either SCC or SCN. Only the nucleus region is shown and bars = 5 *μ*m (A to C) and 10 *μ*m (E).

Next, we examined the potential association of SPF30.1 with putative spliceosomal components in Arabidopsis, including plant orthologues of the human SPF30 interacting partners U2AFs and hPrp3. When expressed alone, SPF30.1 was evenly distributed throughout the nucleus (Fig. 3A). When co-expressed with U2AF65a or U2AF35b, SPF30.1 localized to some subnuclear structures, with its signal overlapping that of the U2AFs (Fig. 3B). Co-expression with RNA-DIRECTED DNA METHYLATION 16 (RDM16), the Arabidopsis homolog of hPrp3 (Huang et al. 2013; Lv et al. 2021), resulted in the enrichment of these proteins in a circular structure in the nucleus (Fig. 3B), reminiscent of the nucleolus localization pattern (Pendle et al. 2005). We also co-expressed SPF30.1 with U1-70K, U1A, U2A, and SR protein SC35, but observed less distinct co-localization signals in subnuclear structures compared to those with U2AFs and RDM16 (Supplementary Fig. S4A). We also tested whether SPF30 protein variants could co-localize with RDM16. Except for SPF30.3, which was excluded from the nucleolus, other tested proteins all co-localized with RDM16 in the nucleolus-like structure (Fig. 3C), suggesting that the central TUDOR domain of SPF30 is involved in this co-localization.

We further tested the potential interaction between SPF30.1 and U2AFs and RDM16 in bimolecular fluorescence complementation (BIFC) assay (Fig. 3D). The reconstruction of the cyan fluorescence protein (CFP) indicates that SPF30.1 interacted with U2AF65a, U2AF35b, and RDM16 in the nucleus (Fig. 3E). When SPF30 was co-expressed with U2AF65a or U2AF35b, CFP was detected throughout the nucleoplasm and was enriched in discrete subnuclear structures, including nucleolus-like and speckled structures (Fig. 3E). When SPF30 was co-expressed with RDM16, CFP was mainly seen in the nucleolus-like structure, with a stronger signal at the periphery of the nucleolus than in the nucleolar cavity (Fig. 3E), similar to those in Fig. 3, B and C. We also observed interaction between SPF30 and U1A in the nucleus, but not with U1-70K nor U2A (Fig. 3E and Supplementary Fig. S4B). Together, our results suggest that SPF30.1 is associated with spliceosomal components in the subnuclear structures of Arabidopsis.

### *SPF30* mutation caused early flowering

**Figure 4.**
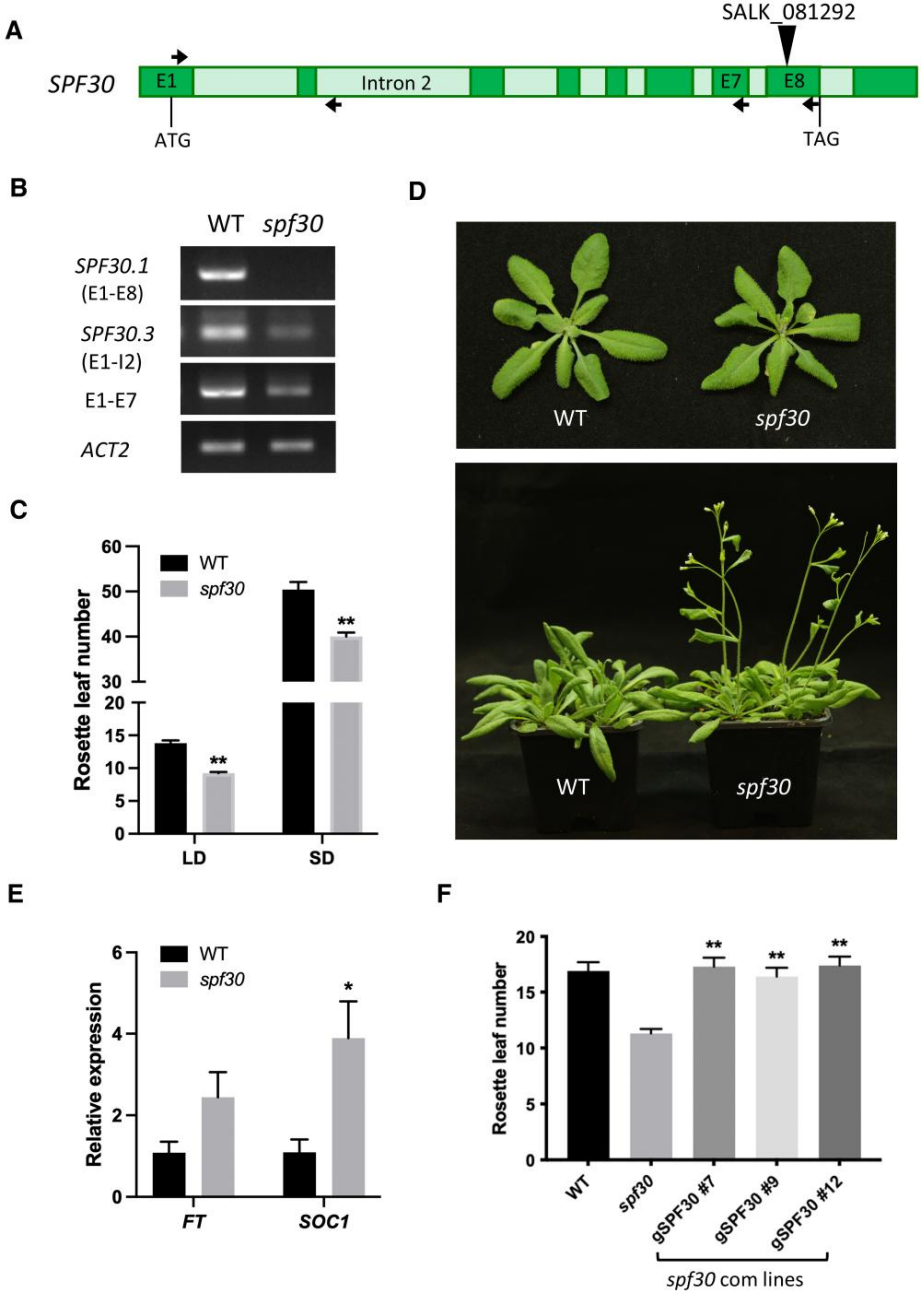
*SPF30* mutation resulted in early-flowering phenotype. **A)** Schematic view of the SALK_081292 (*spf30*) mutant line that carries a T-DNA insertion in the eighth exon of *SPF30*. Exons (E) and introns (I) are shown in dark and light bars, respectively. **B)** RT-PCR validation of the mutant line. WT: wild type. Primers used are indicated by arrows in A. **C)** Numbers of rosette leaf of *spf30* and WT at bolting under long-day (LD) and short-day (SD) conditions. Numbers are shown as means ± SE (*n* ≥ 25). Asterisks indicate significant differences (**, *P* < 0.01; Student's *t*-test). **D)** Phenotypes of *spf30* and WT grown 24 days (top photo) or 30 days (bottom photo) after sowing under LD conditions. **E)** RT-qPCR analysis of flowering genes *FT* and *SOC1* in 12-d-old *spf30* and WT seedlings. Error bars represent SE from three biological replicates. Asterisks indicate significant differences (*, *P* < 0.05; Student's *t*-test). **F)** Numbers of rosette leaf of WT, *spf30*, and *spf30* complemented with the genomic sequence (promoter included) of *SPF30* (*gSPF30*) at bolting. Numbers are shown as means ± SE (*n* ≥ 13). Asterisks indicate significant differences in comparison to *spf30* (**, *P* < 0.01; Student's *t*-test). # indicates independent transgenic lines.

To investigate the physiological role of *SPF30*, we obtained a T-DNA insertion line of *SPF30* (SALK_081292) from Arabidopsis Biological Resource Center (ABRC), which has a T-DNA insertion in the eighth exon of *SPF30* (Fig. 4A). We confirmed the presence of the insertion by genomic PCR analysis and designated the line as *spf30*. The full-length CDS of *SPF30.1* was not detected in the *spf30* cDNA (Fig. 4B), indicating that the production of intact *SPF30.1* was disrupted. The CDS of *SPF30.3* was still detectable but at a lower level (Fig. 4B and Supplementary Fig. S5). Similarly, weakened signals were observed with primers targeting the region before the insertion (Fig. 4B and Supplementary Fig. S5), indicating that a truncated *SPF30* upstream transcript was likely still being produced at low level. Nevertheless, the intact full-length transcripts of all *SPF30* isoforms are expected to be disrupted by the insertion.

The overall morphology of *spf30* was similar to that of wild-type (WT) plants (Fig. 4D). However, the *spf30* plants bolted earlier with significantly fewer rosette leaves compared to WT under both long-day and short-day conditions (Fig. 4, C and D). Meanwhile, RT-qPCR analysis showed that the expression of floral integrator genes *SUPPRESSOR OF OVEREXPRESSION OF CONSTANS 1* (*SOC1*) and *FLOWERING LOCUS T* (*FT*) both increased in the seedlings (Fig. 4E), which is consistent with the observed early-flowering phenotype. To verify if the altered floral transition resulted from *SPF30* loss-of-function, we complemented the mutant with constructs carrying the entire genomic sequence of *SPF30* (*gSPF30*), including its native promoter. As shown in Fig. 4F, introduction of *gSPF30* fully rescued the reduction in the rosette leaf number, indicating that the mutation was responsible for the accelerated floral transition.

### *SPF30* mutation caused reduced transcript levels and splicing efficiency of *FLC*

**Figure 5.**
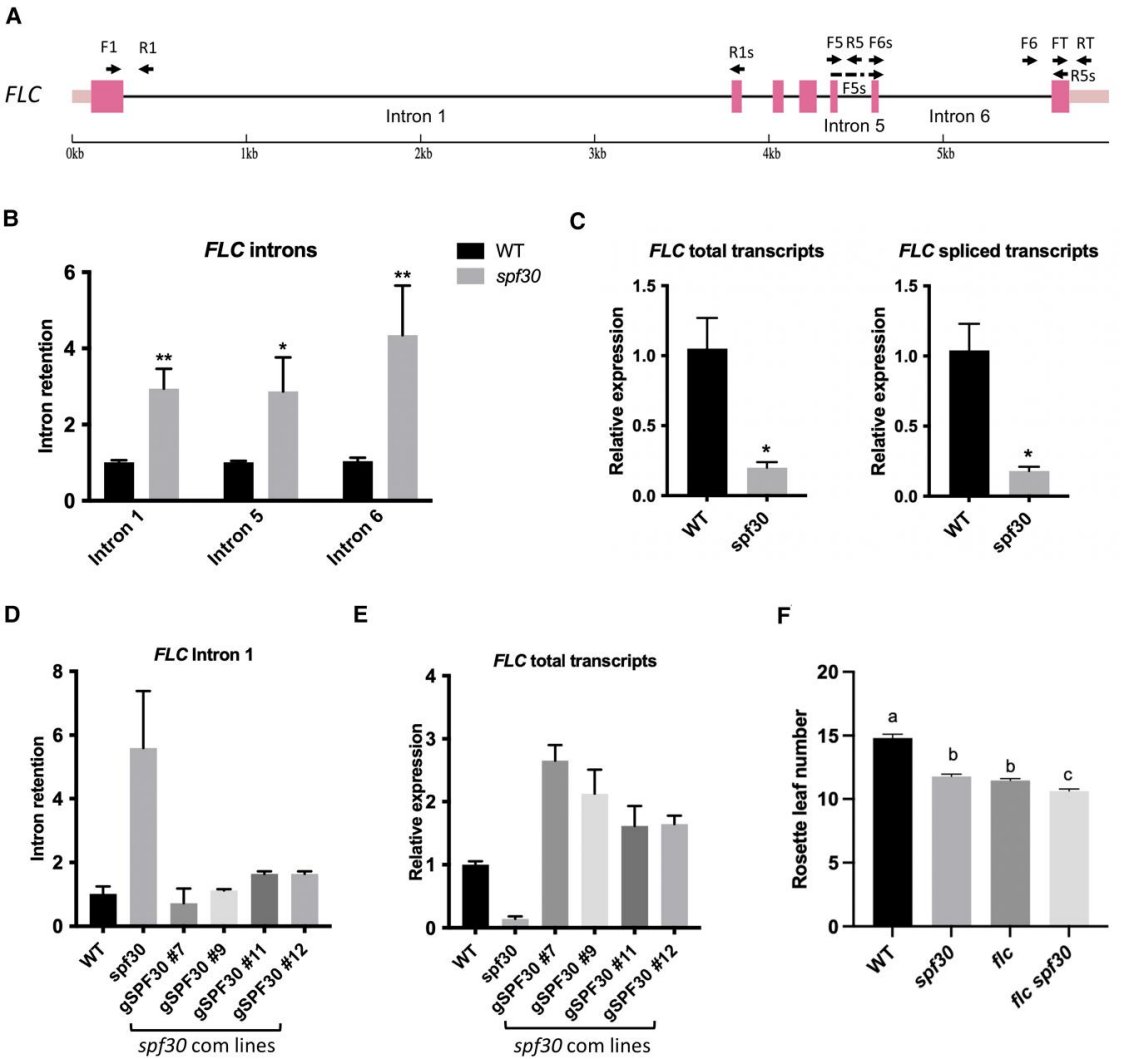
*SPF30* mutation caused reduced transcript levels and increased intron retention of *FLC*. **A)** Schematic view of *FLC* gene structure. Exons are represented by boxes and introns are indicated by lines. Dark boxes indicate coding regions (CDS) and light boxes indicate untranslated regions (UTR). Arrows indicate the primers used for RT-qPCR analysis. **B)** RT-qPCR analysis of *FLC* intron retention in 12-d-old *spf30* and wild-type (WT) seedlings. Intron retention was calculated as the level of unspliced transcripts normalized to the level of spliced transcripts for each detected intron. Specifically, primer pairs F1/R1 and F1/R1s were used for intron 1, F5/R5 and F5 s/R5s for intron 5, and F6/RT and F6s/RT for intron 6. Error bars represent SE from three biological replicates, and asterisks indicate significant differences (*, *P* < 0.05; **, *P* < 0.01; Student's *t*-test). **C)** RT-qPCR analysis of total and spliced *FLC* transcripts in *spf30* and WT. Total transcripts were detected with primer pair FT/RT; spliced transcripts were detected with F1/R1s. Error bars represent SE from three biological replicates, and asterisks indicate significant differences (*, *P* < 0.05; Student's *t*-test). **D** and **E)** RT-qPCR analysis of *FLC* intron 1 retention (D) and total transcript levels (E) in WT, *spf30* and *spf30* complemented with *gSPF30*. Error bars represent SE from two biological replicates. **F)** Rosette leaf number at bolting in WT, *spf30*, *flc* and *flc spf30* under long-day conditions. Numbers are shown as means ± SE (*n* ≥ 36). Different letters indicate significant differences (*P* < 0.05; Tukey's post hoc test).

To investigate the molecular mechanisms of the early-flowering phenotype of *spf30*, we analyzed the expression of several flowering time-related genes by RT-qPCR and found that the mRNA level of *FLC* was greatly reduced in *spf30* (Supplementary Fig. S6). *FLC* is a key repressor of floral induction, located at the convergence point of several flowering pathways (Michaels and Amasino 1999; Boss et al. 2004). Therefore, reduced *FLC* level is consistent with *spf30*'s early flowering. *FLC* has a distinctive long first intron (Simpson 2004) (Fig. 5A), and its introns are sensitive to regulation by splicing (Mahrez et al. 2016; Wang et al. 2020). We analyzed the splicing of the first, fifth, and sixth intron of *FLC*, and found that they were all heavily retained in *spf30* (Fig. 5B), suggesting that the splicing efficiency of *FLC* was reduced in *spf30*. Meanwhile, the levels of both total and spliced transcripts of *FLC* were reduced (Fig. 5C). Moreover, the level of *FLC* transcripts with retained introns significantly increased in CHX-treated seedlings (Supplementary Fig. S7), indicating that these improperly spliced transcripts were subjected to NMD degradation. Therefore, the abnormal splicing of *FLC* might have contributed to the decrease of *FLC* transcript levels.

To confirm that the changes in *FLC* expression were due to *SPF30* loss-of-function, we analyzed *gSPF30* complementation lines and found that *gSPF30* fully restored the splicing efficiency and transcript level of *FLC* (Fig. 5, D and E, Supplementary Fig. S8), indicating that *SPF30* was responsible for the abnormal expression of *FLC*. It has been observed that *FLC* clade genes were similarly regulated in some flowering time mutants mutated in splicing factor genes (Mahrez et al. 2016; Xiong et al. 2019). However, our examination of several *FLC*-like genes showed no decrease in transcript levels or the splicing efficiency of their first intron (Supplementary Fig. S9), suggesting the effects on *FLC* in *spf30* is relatively specific. We also measured the splicing of other flowering-related genes and found increased retention of a 5′UTR intron of *FLOWERING LOCUS K* (*FLK*) (Supplementary Fig. S10), which further indicates the role of *SPF30* in maintaining proper intron splicing. In addition, *FLK* functions upstream of *FLC* in floral regulation (Mockler et al. 2004), so the abnormal splicing of *FLK* could be another factor that influences *FLC* expression.

To further understand the functional relationship between *SPF30* and *FLC*, we crossed *spf30* with *flc* to generate *flc spf30* double mutant. As shown in Fig. 5F, the double mutant *flc spf30* flowered slightly earlier than either *spf30* or *flc* single mutants. However, the extent of this further acceleration in early flowering was relatively modest, suggesting that *SPF30* and *FLC* function in largely overlapping pathways during floral transition. This supports the idea that, while other factors may be involved, *SPF30* affects flowering time mostly through the *FLC* pathway.

### Analysis of the interaction between SPF30.1 and *FLC* RNA in vivo and in vitro

To investigate the molecular role of SPF30 in *FLC* splicing, we performed an RNA immunoprecipitation (RIP) assay using transgenic Arabidopsis plants stably expressing GFP-tagged SPF30.1. RT-qPCR analysis revealed that fragments of *FLC* RNA, including those surrounding its first intron, were significantly enriched by GFP-tagged SPF30.1 compared to the GFP control (Supplementary Fig. S11), indicating an in vivo association between SPF30 protein and *FLC* RNA. Given SPF30's possible association with spliceosomal proteins (Fig. 3), its association with *FLC* may occur directly or directly.

In humans, U2AFs are known to bind sequences near the 3′ end of the introns and play important roles in U2 snRNP recruitment during spliceosomal assembly (Shao et al. 2014). Here we further conducted a microscale thermophoresis (MST) analysis evaluating the binding affinity of U2AF65a, U2AF35b, and SPF30.1 to a synthesized 3′ fragment of the first intron of *FLC* (Supplementary Fig. S12). All three proteins exhibited relatively strong binding affinities toward *FLC*, suggesting direct interaction with *FLC* RNA in vitro. Notably, SPF30.1 displayed the highest binding affinity toward *FLC* among the three. In addition, when *FLC* was mixed with preincubated SPF30.1 and U2AF65a or U2AF35b, the observed binding affinity was higher than that of *FLC* mixed with U2AF65A or U2AF35B alone, suggesting that the presence of SPF30.1 might have a positive effect on their association with *FLC*. These findings further indicate the possibility of direct interaction between SPF30 and *FLC*.

### Complementation effects of the CDSs of individual *SPF30* isoforms

**Figure 6.**
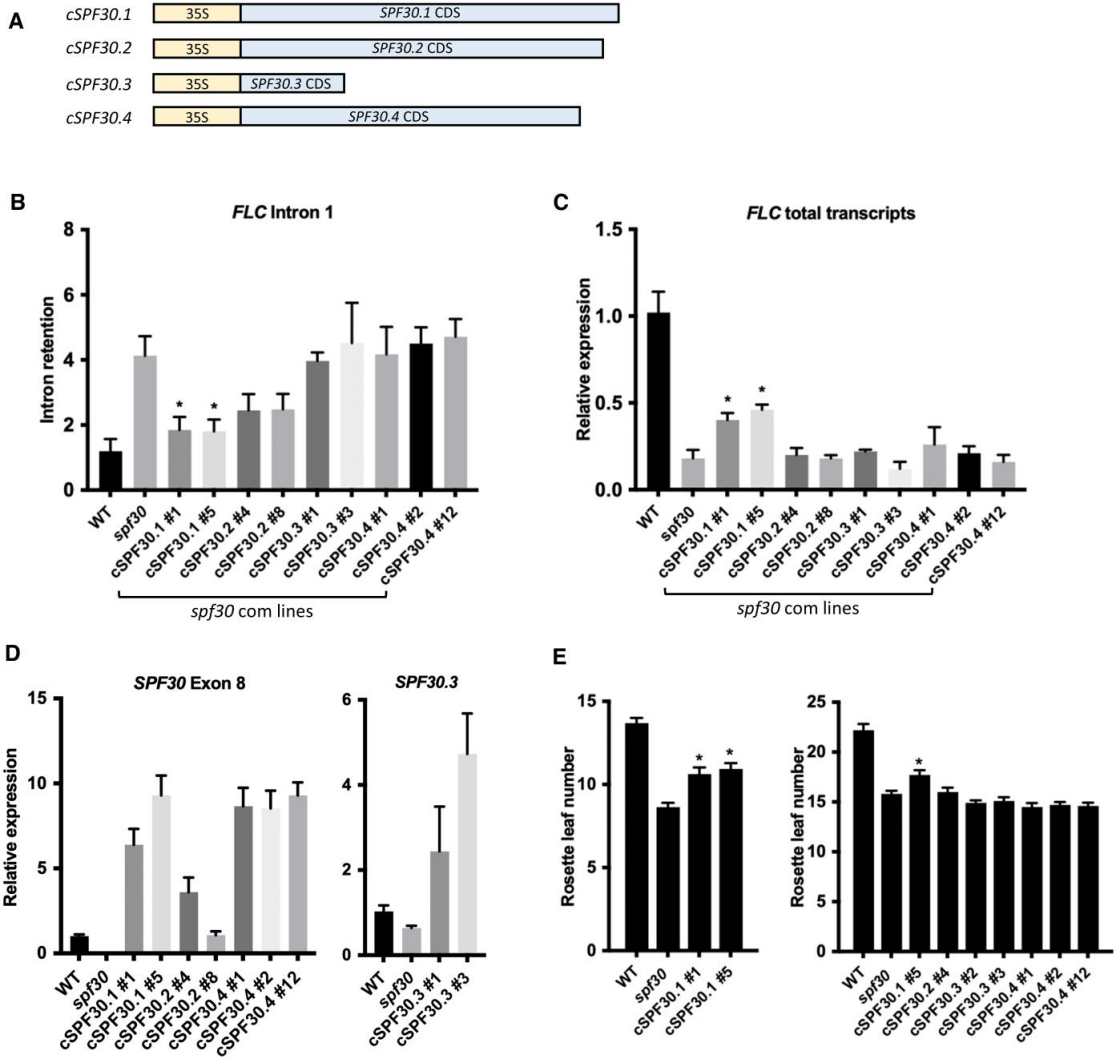
Complementation effects of the CDS of individual *SPF30* transcript isoforms. **A)** Schematic representation of the constructs used for *spf30* complementation. 35S stands for a double CaMV 35S promoter. c stands for coding sequence (CDS). **B** and **C)** RT-qPCR analysis of *FLC* intron 1 retention (B), and total transcript levels (C) in 12-d-old wild-type (WT), *spf30* and *spf30* complemented with individual *SPF30* isoforms. Intron retention was calculated as the ratio of unspliced to spliced *FLC* transcripts. Error bars represent SE from three biological replicates. Asterisks indicate significant differences in comparison to *spf30* (*, *P* < 0.05; Student's *t*-test). **D)** RT-qPCR analysis of *SPF30* expression in *spf30* complementation lines. Error bars represent SE from three biological replicates. For *cSPF30.1*, *cSPF30.2* and *cSPF30.4,* a primer pair spanning the T-DNA insertion site in exon 8 was used; for c*SPF30.3*, isoform-specific primers were used. **E)** Flowering time of *spf30* complemented with c*SPF30.1* (left) or other *SPF30* isoforms (right). Rosette leaf number at bolting is shown as means ± SE (*n* ≥ 16). Asterisks indicate significant differences in comparison to *spf30* (*, *P* < 0.05; Student's *t*-test).

Next, to understand the biological effects of SPF30.1 and its protein variants, we complemented the *spf30* mutant with the CDS of *SPF30.1* to *SPF30.4*, respectively (Fig. 6A), and analyzed their impact on intron splicing. As shown in Fig. 6B, the CDS of *SPF30.1* (*cSPF30.1*) restored splicing of the first intron of *FLC* to a large degree, suggesting that *cSPF30.1* encodes a functional splicing factor that can affect the pre-mRNA splicing of *FLC*. *cSPF30.2* exhibited a weaker yet discernible effect, while *cSPF30.3* and *cSPF30.4* showed limited to no effects (Fig. 6B). A similar pattern was observed in the splicing of *FLK* introns (Supplementary Fig. S13). Expression of the introduced *SPF30* isoforms was detected in all transgenic lines (Fig. 6D). *cSPF30.2* was generally expressed at lower levels than *cSPF30.1*, which may partially account for its reduced effects. The *cSPF30.3* and *cSPF30.4* lines we tested did not show clear rescue effects, despite having expression levels much higher than WT (Fig. 6D), which raises the possibility that these isoforms may not be effectively translated or may produce truncated proteins with limited biological activity, at least for *FLC* and *FLK*.

In terms of *FLC* transcript levels, we found that *cSPF30.1* only exhibited a partial restoration, weaker than its effects on splicing, with the impact of other isoforms being negligible (Fig. 6C). While minor isoforms had little effect on the early-flowering phenotype of *spf30*, *cSPF30.1* showed a modest restoration (Fig. 6E). These observations indicate that, although *cSPF30.1* encodes a functional protein, its efficacy is attenuated compared to *gSPF30* (Fig. 4F), which implies that the presence of certain sequences missing in this construct may be important for the overall function of this gene.

### *SPF30* undergoes extensive NMD-associated AS within its second intron

**Figure 7.**
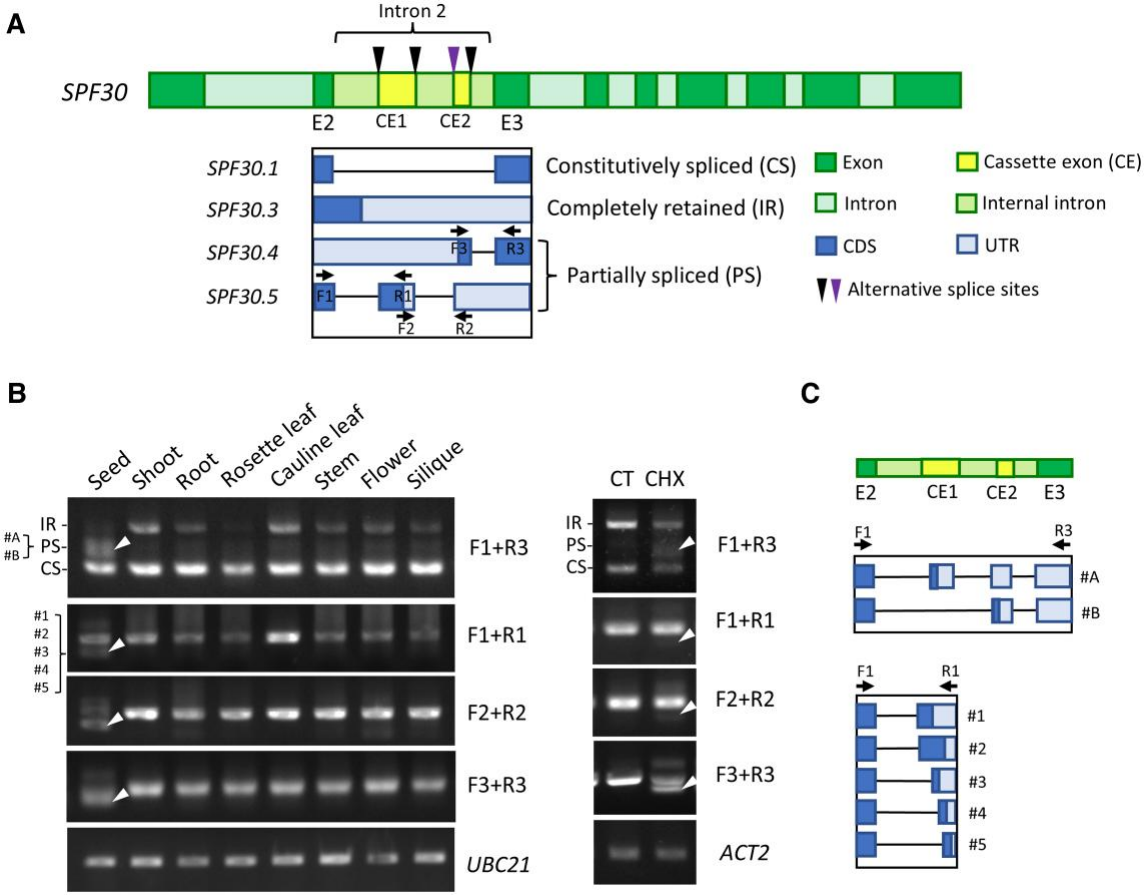
*SPF30* undergoes extensive nonsense-mediated mRNA decay (NMD)-associated alternative splicing (AS) within its second intron. **A)** Schematic view of the second intron of *SPF30* with multiple AS patterns. Arrows indicate primers used for RT-PCR analysis in B. The triangle above the left end of cassette exon 2 (CE2) indicates a previously identified splice site of *SPF30* conserved among dicots. CDS: coding sequence; UTR: untranslated region. **B)** RT-PCR analysis of AS events within the second intron of *SPF30* in various Arabidopsis tissues and in seedlings treated with cycloheximide (CHX). Seedlings treated with water served as control (CT). White triangles indicate bands corresponding to partially spliced intron 2. **C)** Identification of AS patterns within the second intron of *SPF30*. The bands corresponding to partially spliced intron 2 (indicated by white triangles) amplified with primer pairs F1&R1 and F1&R3 in B were analyzed by Sanger sequencing, and the structures of identified AS patterns are shown.

Most *SPF30* isoforms, except *SPF30.2*, resulted from the AS events in the second intron of *SPF30*, so next we analyzed this intron to better understand the generation of these AS events (Supplementary Fig. S1). These splicing events can be categorized into three types depending on the degree of intron 2 splicing: complete removal, as in the case of *SPF30.1*; fully retention, as in the case of *SPF30.3*, or partial splicing, as represented by *SPF30.4* and *SPF30.5* (Fig. 7A). The partially spliced patterns indicate the presence of four cryptic splice sites within the intron (indicated by triangles in Fig. 7A), potentially generating three small internal introns and two cassette exons. Notably, one of these splice sites (indicated by a purple triangle) corresponds to a previously identified conserved splice site of *SPF30* among dicots (Zhang et al. 2019).

We then analyzed the AS of this intron in wild-type Arabidopsis with primers that flank the entire second intron or individual internal introns (Fig. 7A). Our results showed that the second intron was mostly either fully spliced out or retained, except in seeds and under CHX treatment, where partially spliced forms exhibited increased abundance (indicated by white triangles in Fig. 7B). This suggests that the transcripts containing partially spliced second intron were NMD targets. Then we recovered the corresponding gel bands for Sanger sequencing, and, to our surprise, identified various unannotated AS events (Fig. 7C and Supplementary Data Set 2). First, we found that the length of the first internal intron was highly variable, with different splice sites found at the 3′ end of this intron, including the one in *SPF30.5* (marked as #2 in Fig. 7C). However, the most used site was the one located in the middle of all five sites (marked as #3). By using primers flanking the entire second intron, we identified two additional isoform patterns (named as #A and #B in Fig. 7), with the first cassette exon included in #A but skipped in #B. Both #A and #B, although structurally different from *SPF30.4* and *SPF30.5*, share some similar AS features with them. These results show that the conserved intron 2 of SPF30 was spliced with a high degree of flexibility to produce various NMD-sensitive transcripts including, but not limited to, *SPF30.4* and *SPF30.5*.

### The retained second intron in *SPF30.3* can be further spliced

**Figure 8.**
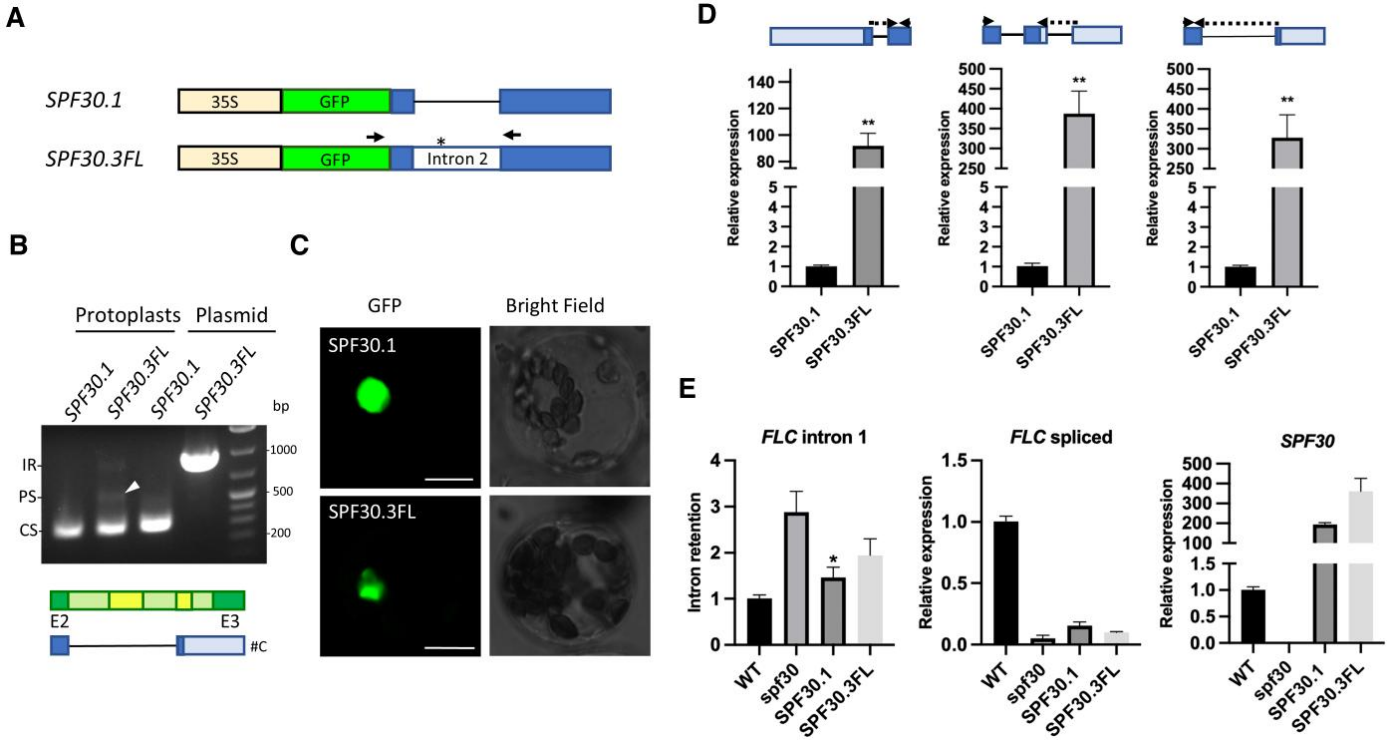
The second intron in the full-length *SPF30.3* transcript (*SPF30.3FL*) can be further spliced in Arabidopsis. **A)** Schematic view of *GFP*-tagged *SPF30.1* and *SPF30.3FL*. 35S indicates a double CaMV 35S promoter. The two boxes flanking intron 2 indicate coding regions (CDS). Arrows indicate primers used in B. Asterisk indicates the position of a stop codon. **B)** RT-PCR analysis of *GFP*-tagged *SPF30.1* and *SPF30.3FL* transcripts expressed in wild-type (WT) Arabidopsis protoplasts. Plasmids carrying *SPF30.1* and *SPF30.3FL* serve as controls. IR, PS and CS represents fully retained, partially spliced and fully spliced second intron, respectively. The band corresponding to partially spliced second intron is indicated by a white triangle and was analyzed by Sanger sequencing. In addition to the isoforms identified in Fig. 7C, an additional product (#C) was detected and illustrated bellow, with a partial *SPF30* genome structure shown for reference. Bar colors are described in Fig. 7A. **C)** Subcellular localization of GFP-tagged SPF30.1 and SPF30.3FL in WT Arabidopsis protoplasts. Bars = 10 *μ*m. **D)** RT-qPCR analysis of *GFP*-tagged *SPF30.1* and *SPF30.3FL* transcripts expressed in protoplast made of *spf30*. The characteristic alternative splicing (AS) patterns (primers indicated by arrows) were analyzed in comparison to the total amount of exogenously expressed *SPF30* transcripts. Error bars represent SE from three biological replicates. Asterisks indicate significant differences (**, *P* < 0.01; Student's *t*-test). **E)** RT-qPCR analysis of *FLC* intron 1 retention, spliced *FLC* transcript levels and exogenous *SPF30* expression levels in WT, *spf30* and *spf30* protoplasts transfected with *GFP*-tagged *SPF30.1* or *SPF30.3FL* transcripts. Error bars represent SE from three biological replicates. Asterisks indicate significant differences in comparison to *spf30* (*, *P* < 0.05; Student's *t*-test).

In contrast to *SPF30.4* and *SPF30.5*, *SPF30.3* is immune to CHX treatment despite carrying a PTC (Fig. 1C and Supplementary Fig. S2C), suggesting a role different from those NMD-sensitive isoforms. Moreover, we noticed that the increase in the amount of *SPF30.4* and *SPF30.5* somehow coincided with reduction in the amount of *SPF30.3* (Figs. 1B and 7B, Supplementary Fig. S2B), so we wondered whether *SPF30.4* and *SPF30.5* could be produced from *SPF30.3*. To test this, we expressed the full-length transcript of *SPF30.3* (*SPF30.3FL*; Supplementary Data Set 1), which carries the retained second intron, in Arabidopsis with an N-terminal GFP tag (Fig. 8A), and detected three bands corresponding to the fully retained, partially spliced and constitutively spliced intron 2, respectively (Fig. 8B), indicating that *SPF30.3* was processed into shorter isoforms in vivo. Particularly, Sanger sequencing revealed that the predominant form of the middle band (indicated by a white triangle) had a structure (labeled as #C in Fig. 8B; Supplementary Data Set 2) different from those in Fig. 7C. Meanwhile, GFP-tagged SPF30.3FL localized to the nucleus in Arabidopsis protoplasts (Fig. 8C), further indicating that the second intron in *SPF30*.3 *FL* was spliced out to generate *SPF30.1*. Meanwhile, expression of *SPF30.3FL* also led to a significant increase in AS patterns that are characteristic in *SPF30.4* and *SPF30.5*, respectively (Fig. 8D). Similar results were observed with *SPF30.3FL* expressed in Nicotiana leaves (Supplementary Fig. S14). In summary, our results show that artificially expressed *SPF30.3FL* can be further spliced into either *SPF30.1*, or *SPF30.4*/.5-like isoforms that are potential NMD targets in plant cells, which suggests the potential role of *SPF30.3* as an intermediate transcript for further procession.

### The second intron negatively affects the function of *SPF30*

To investigate the biological role of the second intron of *SPF30*, we analyzed the splicing and expression of *FLC* in *spf30* protoplasts transfected with GFP-tagged *SPF30.1* or *SPF30.3FL*. Interestingly, the complementary effects of *SPF30.3FL* was weaker than *SPF30.1*, despite its slightly higher expression (Fig. 8E), suggesting that intron 2 is not required for *SPF30* function and may instead exert a negative effect on its activity.

**Figure 9.**
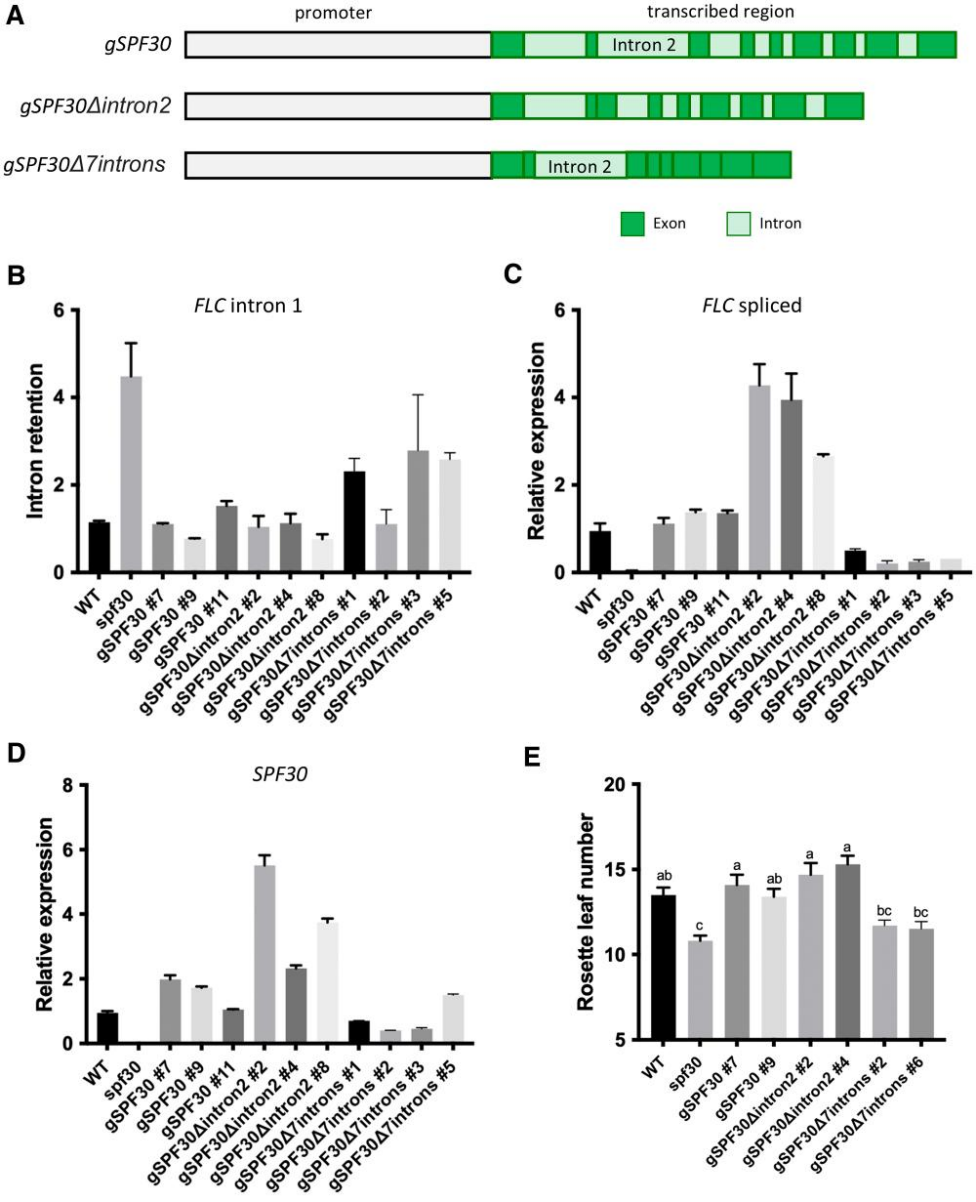
The second intron negatively impacts the function of *SPF30*. **A)** Schematic representation of *SPF30* genomic deletion constructs. *gSPF30Δintron2* lacks the second intron, while *gSPF30Δ7introns* retains only intron 2, with the rest seven introns removed. **B** to **D)** RT-qPCR analysis of *FLC* intron 1 retention (B), spliced *FLC* transcript levels (C), and exogenous *SPF30* expression (D) in 12-d-old wild-type (WT), *spf30* and *spf30* complemented with different *SPF30* genomic constructs. # indicates independent transgenic lines. Error bars represent SE from two technical replicates. **E)** Flowering time of *spf30* lines complemented with *SPF30* genomic constructs. Rosette leaf number at bolting is shown as means ± SE (*n* ≥ 16). Different letters indicate significant differences (*P* < 0.05; Tukey's post hoc test).

To further understand the roles of this intron in transgenic plants, we constructed two *SPF30* genomic constructs (Fig. 9A): one with only the second intron deleted (*gSPF30Δintron2*), and another with all introns except intron 2 deleted (*gSPF30Δ7introns*). Both constructs were introduced into *spf30*, and their effects were compared with the wild-type *SPF30* genome (*gSPF30*). Analysis of exogenous *SPF30* levels in these lines revealed that *gSPF30Δintron2* was generally expressed at higher levels than *gSPF30* (Fig. 9D), suggesting that the presence of intron 2 may suppress the transcript abundance of *SPF30*, possibly through AS-NMD. The elevated *SPF30* expression in *gSPF30Δintron2* lines roughly coincided with increased *FLC* levels (Fig. 9C) and a modest delay in flowering time (Fig. 9E). These observations further support a negative role for intron 2 in regulating *SPF30* function, likely at least in part through downregulating its expression level in these stable transgenic lines. Interestingly, the effects of *gSPF30Δintron2* on *FLC* splicing was similar to *gSPF30* (Fig. 9B), probably because the splicing efficiency was already maximized and could not be further enhanced.

In contrast*, gSPF30Δ7introns* showed reduced *SPF30* expression (Fig. 9D), exhibited weaker complementary effects on *FLC* splicing and expression (Fig. 9, B and C), and only partially restored the early-flowering phenotype (Fig. 9E). These results suggest that, while intron 2 plays a negative role, the remaining introns likely play a positive role in *SPF30* expression and function.

### The AS in the second intron of *SPF30* is regulated by light and temperature signals

**Figure 10.**
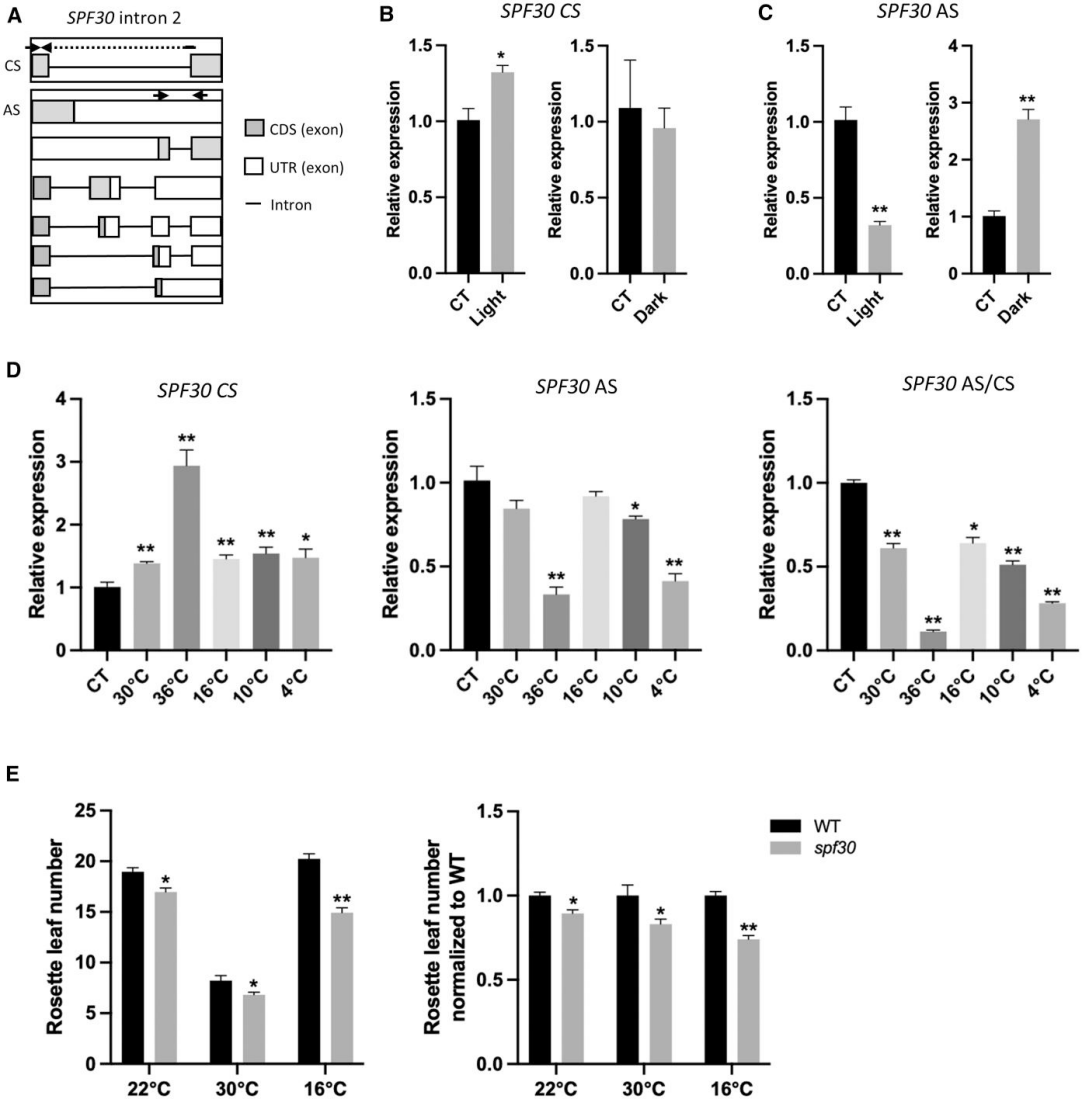
Alternative splicing (AS) in the second intron of *SPF30* is regulated by light and temperature. **A)** Schematic view of the second intron of *SPF30* with multiple AS events. Arrows indicate primers used for RT-qPCR analysis. CS refers to complete splicing of intron 2, whereas AS includes all isoforms containing retained intron 2 fragments. 2. **B** and **C)** RT-qPCR analysis of fully spliced *SPF30* (B) and total AS events (C) in 12-d-old Arabidopsis seedlings upon light and dark treatments. CT: control. Ambient temperature was maintained at approximately 23 °C. **D)** RT-qPCR analysis of fully spliced *SPF30*, the AS events of *SPF30* intron 2, and the ratio of AS to CS in the 12-d-old Arabidopsis seedlings exposed to different temperatures. CT: control (23 °C). **B** to **D)** Error bars represent SE from three or more biological replicates. Asterisks indicate significant differences (*, *P* < 0.05; **, *P* < 0.01; Student's *t*-test). **E)** Flowering time of WT and *spf30* grown under different temperature conditions. For temperature treatments, plants were transferred to 30 °C or 16 °C after 6 days of growth at 22 °C. Rosette leaf number is shown as means ± SE (*n* ≥ 38). Asterisks indicate significant differences (*, *P* < 0.05; **, *P* < 0.01; Student's *t*-test).

To understand the physiological implication of the characterized AS in *SPF30*, we analyzed its patterns under different environmental conditions. For measuring the levels of total AS events, we designed a primer pair to recognize all known AS events associated with intron 2 *in SPF30* and another primer pair specific to the fully spliced form (i.e. *SPF30.1*), as indicated by arrows in Fig. 10A. For light/dark treatments, the ambient temperature was maintained at approximately 23 °C. Under light treatments, AS levels reduced significantly, coinciding with an increase of the fully spliced *SPF30* (Fig. 10, B and C). Conversely, under dark treatment, an opposite pattern was observed. These findings suggest that AS in *SPF30* is regulated by light signals, with the complete removal of *SPF30* intron 2 being promoted by light but inhibited by dark. Given a previous report showing that *SPF30* is involved in circadian clock regulation(Romanowski et al. 2020), it is likely that *SPF30* responses to day–night cycles through light-mediated changes in its AS patterns.

We observed that AS events reduced under all five tested temperatures (i.e. 30 °C, 36 °C, 16 °C, 10 °C, and 4 °C) compared to the control condition (23 °C), with more pronounced reductions at extreme temperatures such as 36 °C and 4 °C (Fig. 10D). Meanwhile, the levels of fully spliced *SPF30* transcripts increased, indicating that the production of the primary *SPF30.1* is prioritized over alternative isoforms under these temperatures (Fig. 10D). Additionally, the difference in flowering time between WT and *spf30* was slightly more pronounced at 16 °C and 30 °C, compared to 22 °C (Fig. 10E), which is consistent with a higher expression of *SPF30*.1 under these circumstances.

Overall, our findings suggest that the AS patterns of *SPF30* are susceptible to light and temperature signals, suggesting that they might be involved in the regulation of *SPF30.1* levels in response to these environmental cues.

## Discussion

### SPF30 is associated with spliceosomal core proteins

In this study, we report the functional characterization of Arabidopsis SPF30 as a plant splicing factor. In human, SPF30 is known as a U2 snRNP-related spliceosomal protein that simultaneously interacts with U2AF35 and hPrp3 during spliceosomal assembly (Little and Jurica 2008). Plant homologs of human U2AFs (i.e. U2AF35 and U2AF65) and hPrp3 have been identified with functional involvement in pre-mRNA splicing, suggesting that they are parts of the plant splicing machinery (Wang and Brendel 2006; Huang et al. 2013; Park et al. 2019; Xiong et al. 2019; Lv et al. 2021). Here, we found that Arabidopsis SPF30 interacts with both U2AFs predominantly in the nuclear speckles (Fig. 3), supporting its putative role as a U2 snRNP-related spliceosomal protein in plants. Moreover, the observed co-localization and interaction between SPF30 and RDM16 in the nucleus further suggests that the associations between SPF30, U2AFs and tri-snRNP components might be conserved across plants and animals.

### *SPF30* is important for floral transition and *FLC* expression

Our analysis of the *spf30* loss-of-function mutant revealed that it has accelerated floral transition, consistent with a previous report that observed a similar phenotype in two *spf30* mutant lines (Romanowski et al. 2020), including the one used in our study. Here, we further reported the molecular mechanisms underlying this phenotype, which appears to involve the impaired expression and splicing of *FLC*. Many studies have reported that floral transition is sensitive to regulation by AS, and mutations in splicing factor genes often led to altered flowering time (Carvalho et al. 2013; Staiger and Brown 2013; Wang et al. 2020). Particularly, mutations in several U2 snRNP-related genes in Arabidopsis, including *U2AF65a*, *U2AF65b* and *SPF45*, also affect both flowering time and *FLC* expression (Xin et al. 2017; Park et al. 2019; Xiong et al. 2019). Our data on *SPF30* further indicate that there is a strong association between U2 snRNP and flowering time control. U2 snRNP is one of the earliest snRNPs to assembly onto pre-mRNA and is vital for intron recognition. Therefore, as a putative U2-related spliceosomal member, the absence of SPF30 may affect the normal function of U2 snRNP in recognizing specific introns. Here, we further provide evidence that SPF30 has a high binding affinity toward *FLC* RNA, implying that it might have a role in enhancing or bridging the association between U2AFs and *FLC* pre-mRNA, thereby facilitating the assembly of U2 snRNP. In the future, specific motifs in *FLC* targeted by SPF30 and U2AFs could be analyzed to determine how these motifs affect the efficiency of *FLC* splicing.

In addition to altered splicing, the transcript levels of *FLC* also greatly reduced in *spf30*, which can be partially attributed to impaired intron splicing (Supplementary Fig. S7). However, the effects of different *SPF30* constructs on *FLC* splicing and expression are not always aligned. For example, complementation with *SPF30.1* CDS largely restored the splicing defect but had only a modest effect on *FLC* transcript levels (Fig. 6). Moreover, deletion of intron 2 in *gSPF30* further elevated *FLC* levels without noticeable effects on its splicing (Fig. 9). These discrepancies suggests that SPF30 may influence *FLC* transcription, at least partially, through a mechanism independent of its splicing regulation, and it seems that introns other than intron 2 in *SPF30* are involved in this process (Fig. 9).

### *SPF30* itself is regulated via AS of its second intron

In this study, we also presented a detailed examination of *SPF30* splice isoforms, and revealed that its conserved second intron is extensively spliced to generate NMD-targeted isoforms, a regulatory mechanism found in many splicing factor genes for homeostatic control of protein levels (McGlincy and Smith 2008; Palusa and Reddy 2010). In a recent iso-sequencing analysis of Arabidopsis transcriptome, more than 40 isoforms of *SPF30* were annotated, the majority of which involve AS in the second intron and are predicted to be NMD targets (Zhang et al. 2022), consistent with our observation. These observations suggest this intron might have evolved for gene expression regulation through AS-NMD. Our functional analyses based on deletion of the entire second intron (Figs. 8E and 9) further indicate that it plays a negative role in the expression and function of *SPF30*, while, interestingly, the other introns seem to have opposite roles.

Moreover, we observed that the fully retained second intron in *SPF30.3* can be further spliced into various shorter isoforms. In plants, intron retention is the most common type of AS (Marquez et al. 2012; Zhang et al. 2022), and many transcripts with retained introns were found retained in the nucleus and resistant to NMD (Kalyna et al. 2012; Göhring et al. 2014; Jia et al. 2020). However, the biological roles of these intron-retained transcripts remain poorly studied. One hypothesis is that they serve as reservoirs for post-transcriptional splicing upon environmental or developmental signals (Göhring et al. 2014; Jia et al. 2020; Petrillo 2023). Our analysis of *SPF30.3* supports this view and further suggests that such post-transcriptional splicing can be combined with AS-NMD to form a more flexible regulation mechanism. Furthermore, overexpression of the CDSs of *SPF30.3* and *SPF30.4* had limited biological effects on *FLC* splicing (Fig. 7), suggesting that their primary roles may lie at the mRNA level. Alternatively, these isoforms may have dominant-negative effects upon translation, which again, would enable negative regulation. *SPF30.2*, on the other hand, might also function in the negative regulatory loop by encoding a less active protein; however, the endogenous *SPF30.2* was not detected so its existence remains uncertain.

**Figure 11.**
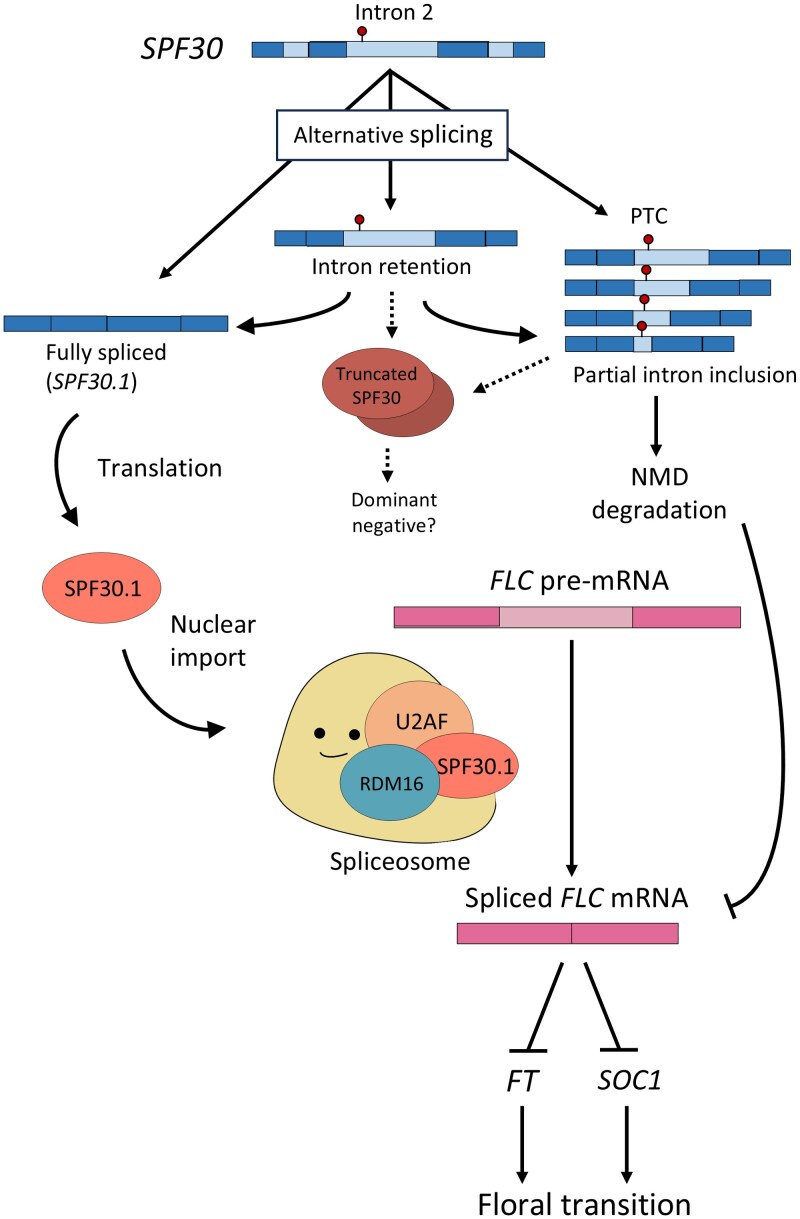
Working model of *SPF30*. *SPF30* is alternatively spliced into various transcript isoforms. The fully spliced *SPF30.1* encodes the full-length SPF30.1 protein, which is associated with spliceosomal components and is required in floral transition by affecting *FLC* expression and splicing. Alternative isoforms with partially retained intron 2 carry a premature termination codon (PTC) and are targeted for nonsense-mediated mRNA decay (NMD), enabling a negative regulation. Isoforms with intron 2 fully retained are immune to NMD and can serve as intermediate transcripts for further splicing to produce *SPF30.1* or NMD-sensitive transcripts. By generating these different isoforms, the effects of the functional SPF30 protein can be fine-tuned in the cells. Dotted lines indicate hypothetical pathways.

Here, we propose a working model to illustrate both the role of *SPF30* in floral transition and its own regulation by AS (Fig. 11). Briefly, the second intron of *SPF30* is spliced into three different patterns. Among them, *SPF30.1* encodes a functional splicing factor that is recruited into the spliceosome and regulates the splicing of downstream genes such as *FLC*. *SPF30.4, SPF30.5*, and other isoforms with partially retained intron 2 are subjected by NMD, enabling a AS-NMD-mediated negative feedback. *SPF30.3* serves as an intermediate transcript that can be post-transcriptionally spliced into either *SPF30.1* or NMD-sensitive *SPF30.4/.5*-like isoforms, likely depending on cellular demand. By generating various alternative isoforms with different regulatory roles, *SPF30* expression can be modulated in a flexible and economic way. Supporting this, we observed that the AS patterns of *SPF30* vary across tissues (Fig. 1B; Supplementary Fig. S2) and in response to environmental stimuli such as light and temperature (Fig. 10), suggesting that AS regulation likely acts in parallel with transcriptional regulation to fine-tune the production of SPF30.1 under varying physiological conditions.

In plants, splicing factor genes often undergo extensive AS, yet how they are regulated via AS remains poorly studied. Our model of *SPF30* provides an example of how these isoforms are orchestrated to regulate gene expression, offering a potential framework for studying other splicing factor genes with similar AS patterns, such as some SR genes that exhibit multiple AS events associated with one specific intron (Reddy and Shad Ali 2011). Our findings suggest that fine-tuning the levels of splicing factors by AS might constitute a delicate post-transcriptional regulation network in plants, allowing the cellular transcriptome to be rapidly and efficiently shaped to adapt to various developmental and environmental changes.

In summary, in this study we characterized SPF30 as a pre-mRNA splicing factor in Arabidopsis and revealed its importance for floral transition and *FLC* expression. Furthermore, using *SPF30* as an example, we show how a splicing factor gene itself can be regulated through the generation of various isoforms with distinct roles.

## Materials and methods

### Plant materials and growth conditions

Arabidopsis (*A. thaliana*) ecotype Columbia-0 (Col-0) was used as the wild-type (WT) plant in this study. Seeds of the T-DNA insertion line *spf30* (SALK_081292; AT2G02570) was obtained from the ABRC. Arabidopsis seeds were surface-sterilized with 20% bleach, and were stratified at 4 °C in the dark for 2 days before germination on Murashige and Skoog (MS) medium (Murashige and Skoog 1962) supplemented with 1.0% (w/v) agar and 1.0% (w/v) sucrose. Unless indicated otherwise, plants were grown at 22 to 24 °C under long-day conditions (16 h light/8 h dark photoperiod) with a light intensity of 100 to 150 *μ*mol m^−2^ s^−1^. For short-day conditions, plants were grown under an 8 h light/16 h dark photoperiod. The *flc* mutant was described previously (Xiong et al. 2019) and the *flc spf30* double mutant was generated by crossing *flc* with *spf30*.

To analyze the expression of *SPF30* in different tissues, shoots and roots were collected from 12-d-old seedlings grown on MS medium. Rosette leaves were collected from 4-wk-old plants grown in soil, and cauline leaves, stems, siliques, and inflorescences were collected from 6-wk-old plants. For inhibition of the NMD pathway, 12-d-old seedlings grown on MS medium were treated with 100 *μ*g/ml cycloheximide (CHX) for 3 h at room temperature. Seedlings treated with water served as controls. For flowering time measurement, the number of rosette leaves at bolting was counted for at least 13 plants per group under the indicated photoperiods.

### Light and temperature treatments

To analyze the AS of *SPF30* under environmental signals, 12-d-old WT Arabidopsis seedlings grown on MS medium were subjected to various conditions. For the dark treatment, plates were wrapped by aluminum foil for 6 h, while control plants were kept under normal light conditions. For the light treatment, plates were first kept in the dark for 4 h and then exposed to light for 2 h, while control plants were kept in the dark for the entire 6 h. During light/dark treatment, plants were kept at an ambient temperature of approximately 23 °C. For temperature treatments, aluminum foil-wrapped plates were placed in incubators set at 36 °C, 30 °C, 23 °C (control), 16 °C, 10 °C, and 4 °C for 6 h.

### RNA extraction and PCR analysis

Plant tissues were harvested and were ground to fine powder in liquid nitrogen. Total RNA was extracted from approximately 100 mg sample or 10^5^ protoplast cells using Plant RNA Purification Reagent (Invitrogen, USA) according to the manufacturer's instructions. Genomic DNA was removed using RNase-free DNase I (Transgen, China). The integrity of extracted RNA was verified via electrophoresis on a 1% agarose gel and concentration was measured by Nanodrop spectrophotometer (Thermo scientific, USA). RNA was transcribed to cDNA with oligo-dT primers in a 20-*μ*l reaction volume using First Strand cDNA Synthesis Kit (Thermo scientific, USA).

For reverse transcription-PCR (RT-PCR) analysis, the resulting cDNA was diluted by 5-fold and 1 *μ*l was used as template for PCR amplification in a 10-*μ*l reaction volume using 2×Taq PCR StarMix (GenStar, China). For identification of isoforms, DNA fragments were purified from DNA gel by the E.Z.N.A. Gel Extraction Kit (Omega, Norcross, GA, USA) and were cloned using the TA/Blunt-Zero Cloning Kit (Vazyme, Nanjing, China) for subsequent Sanger sequencing performed by BGI (Shenzhen, China).

For reverse transcription-quantitative PCR (RT-qPCR) analysis, each 10 *μ*l reaction mixture contained 5 *μ*l of TransStart qPCR SuperMix (Transgen, China), 0.5 *μ*l of cDNA template and 0.5 *μ*M of each primer. The PCR reactions were run on the CFX96/384 Real-Time Systems (Bio-Rad, USA) with an initial denaturation at 95 °C for 1 min, followed by 40 cycles at 95 °C for 10 s, 56 °C for 10 s and 72 °C for 8 s. Relative expression levels between different groups were calculated using the 2^−ΔΔCt^ method. Unless otherwise indicated, data are presented as mean ± standard error (SE) from two or more biological replicates. Arabidopsis *ACTIN2* (*ACT*2) or *TUBULIN2* (*TUB2*) was used as the internal reference gene. The retention of introns was measured as the level of unspliced transcripts normalized to the level of spliced transcripts for each detected intron. All primers used for PCR analysis are listed in Supplementary Table S1.

### Plasmid construction

CDSs of *SPF30.1*, *SPF30.3*, *SPF30.4*, SPF30ΔCter and the sequence of *SPF30.3FL* were amplified from Arabidopsis cDNA. The CDS of *SPF30.2* was constructed using overlap extension PCR method using the CDS of *SPF30.1*. For expression in the plant cells, the amplified sequences were firstly cloned into entry vector pENTR/D-TOPO (Invitrogen, USA) and then re-cloned into binary vectors by LR recombination reaction. To generate GFP-SPF30 constructs, sequences of *SPF30* variants were transferred into binary vector pMDC43 (with a N-terminal GFP tag). To produce overexpression lines, sequences were transferred into binary vector pMDC32 under the control of a double CaMV 35S promoter. To produce promoter-*GUS* constructs, the 2,000-bp region upstream of the end of the first exon of *SPF30* was amplified from Arabidopsis genomic DNA and put into pENTR/D-TOPO, then re-cloned into binary vector pMDC163 (with a *GUS* reporter gene). The full genomic sequence of *SPF30* (*gSPF30*), starting from roughly 2,000 bp upstream of the 5′UTR of *SPF30* and ends shortly after the 3′UTR of *SPF30*, and its two deletion variants *gSPF30Δintron2* and *gSPF30Δ7intron2* were cloned from Arabidopsis genomic DNA into binary vector pEGFC-H by a company (Edgene, Wuhan, China).

To generate RFP-tagged constructs, the respective CDSs were amplified from Arabidopsis cDNA with restriction sites (Supplementary Table S1), digested with corresponding enzymes, and inserted into pSAT4A-mCherry-N1 (with a C-terminal mCherry tag), driven by a double CaMV 35S promoter. For bimolecular fluorescence complementation (BIFC) assay, the CDS of *SPF30.1* was inserted into the pSCYCE(R) vector with a N-terminal SCFP3A_C155_ tag, whereas other CDSs were inserted into the pSCYNE vector with a C-terminal SCFP3A_N173_ tag (Waadt et al. 2008). For prokaryotic expression, CDSs of *SPF30.1*, *U2AF65a* and *U2AF35b* were cloned into pET-30a plasmid.

### Construction of transgenic lines

Binary vectors carrying corresponding recombinant plasmids were transformed to *Agrobacterium tumefaciens* strain GV3101 by freeze–thaw method (Jyothishwaran et al. 2007). For promoter analysis, *Agrobacterium* strain carrying the *SPF30pro::GUS* construct was used to stably transform WT Arabidopsis by floral dipping method (Clough and Bent 1998). The primary transformants (T_0_) seeds were screened on MS medium containing 50 *μ*g/ml hygromycin. T_1_ seedlings with true leaves were planted in soil for self-fertilization, and seeds from five to ten independent lines were individually harvested. In the T_2_ generation, lines that displayed a Mendelian 3:1 segregation ratio on MS medium with hygromycin (an indicator of a single-copy insertion) were thus selected. In the T_3_ generation, lines that are fully hygromycin-resistant were used for subsequent analysis. For construction of complementation lines, *Agrobacterium* strain GV3101 carrying pMDC32 derivatives were used to stably transform *spf30* by floral dipping similarly. The transformants were identified through antibiotic selection and PCR confirmation, and two to four independent lines were used for phenotype analysis and RT-qPCR analysis.

### Histochemical GUS staining

For histochemical staining, 10-d-old seedlings grown on MS medium were collected. Rosette leaves were collected from 4-wk-old plants and cauline leaves, stems, siliques, and flowers were collected from 6-wk-old plants grown in soil. Embryos were derived from mature seeds. Harvested plant tissues were immersed and vacuum-infiltrated in GUS staining solution [50 mm sodium phosphate (pH 7.5), 2 mm K_3_[Fe(CN)_6_], 2 mm K_4_[Fe(CN)_6_], 0.1% (v/v) Triton X-100, 1 mg/ml X-Gluc] for 1 h, followed by 2 to 3 h of incubation at 37 °C. After that, tissues were washed and kept in 70% ethanol to removal chlorophyll. GUS-stained samples were photographed using a light stereomicroscope (Olympus SZX16).

### Subcellular localization assay in Arabidopsis protoplast

pMDC43 plasmids carrying *GFP*-*SPF30* construct were extracted from *E. coli* DH5α and concentrated to 1 *μ*g/*μ*l. Protoplasts were prepared according to the “tape-Arabidopsis sandwich” approach (Wu et al. 2009) with a concentration of 2 to 5 × 10^5^ cells/ml in MMG solution (0.4 m mannitol, 15 mm MgCl_2_ and 4 mm MES buffer; pH: 5.7). For transformation, 10 *μ*g of DNA, 100 *μ*l of prepared protoplasts and 110 *μ*l of polyethylene glycol (PEG) solution [30% PEG4000 (Sigma- Aldrich, USA), 0.2 m mannitol and 0.1 m CaCl_2_] were gently mixed and incubated for 15 min. For co-expression of two different proteins, 5 *μ*g of each plasmid was used. After incubation, 440 *μ*l of W5 solution (154 mm NaCl, 125 mm CaCl_2_, 5 mm KCl, 5 mm glucose and 2 mm MES buffer; pH: 5.7) was added slowly to stop the reaction. Finally, protoplasts were pelleted by centrifugation (200 × *g*), washed twice with W5 and incubated in 6-well plates at room temperature at low light conditions. After 24 to 36 h, transformed protoplasts were photographed by confocal microscope (Leica TCS SP8 imaging system) or harvested for RNA extraction. For fluorescence acquisition, GFP signals were detected with a 488 nm laser (excitation) and 490 to 530 nm emission filter, and RFP signals with a 552 nm laser and 590 to 630 nm emission.

### Subcellular localization and BIFC in Nicotiana leaves

For expression in Nicotiana (*N. benthamiana*) leaves, *Agrobacterium* GV3101 strains carrying corresponding constructs were grown overnight at 28 °C in LB medium. Bacterial cultures were centrifuged and resuspended in 10 mm MgSO_4_ supplemented with 200 *μ*M acetosyringone to reach an optical density at 600 nm (OD_600_) of 0.6. Cultures were incubated at room temperature for 2 h. For BIFC, two different agrobacterium cultures were evenly mixed before incubation. After incubation, bacterial cultures were infiltrated into fully expanded leaves of 6-wk-old Nicotiana plants. Infiltrated plants were kept in the dark at high humidity overnight. The following morning, plants were brought to a normal light/dark cycle. After two days, Nicotiana leaves were examined by a confocal microscope (Leica TCS SP8 imaging system) or were harvested for RNA extraction. For fluorescence acquisition, GFP signals were detected with 488 nm excitation and 490 to 530 nm emission, and CFP signals with 405 nm excitation and 460 to 520 nm emission.

### RNA immunoprecipitation

8-d-old transgenic Arabidopsis seedlings overexpressing GFP-tagged SPF30.1 or GFP grown under long-day conditions were ground into powder and fixed with 1% formaldehyde for 15 min, followed by quenching with 125 mm glycine. The extract was lysed in RIP lysis buffer [50 mm Tris-HCl (pH 7.5), 150 mm NaCl, 4 mm MgCl_2_, 0.25% Igepal CA-630, 1% SDS, 0.25% sodium deoxycholate, 5 mm DTT] supplemented with RNase Inhibitor and an EDTA-free Protease Inhibitor Cocktail (Roche). The extract was incubated with Anti-GFP mAb-Magnetic Agarose beads (MBL) for 3 h at 4 °C, followed by extensive washing with RIP lysis buffer for eight times. Before adding the Anti-GFP beads, 10% of the extract was taken as input. Co-precipitated RNAs and RNAs in the input were extracted with Tri Reagent (Sigma-Aldrich) following the manufacturer's instructions.

### MST assay

Recombinant SPF30.1, U2AF65a and U2AF35b proteins were expressed in *E. coli* strain Transetta (DE3) and purified using His-tag Protein Purification Kit P2226 (Beyotime, China). A 60-nt 3′ fragment of the first intron of *FLC* was synthesized with a 3′ CY5 label by Sangon Biotech Company (China). For quantification of binding affinity, fluorescently labeled *FLC* RNA fragment at a concentration of 4 nm was mixed with serially diluted ligands. Each sample was loaded into premium-coated capillaries (NanoTemper, Germany), and the measurements were performed using the Monolith NT.115 instrument (NanoTemper, Germany). The binding curves and dissociation constant (*K_d_*) were determined by the MO.Analysis v2.3 software. Values represent mean ± SD of two independent measurements.

### Accession numbers

The main genes involved in this study and their accession numbers are as follows: *SPF30* (AT2G02570), *U2AF65a* (AT4G36690), *U2AF35b* (AT5G42820), *RDM16* (AT1G28060), *U1-70K* (AT3G50670), *U1A* (AT2G47580), *U2A* (AT1G09760), *SC35* (AT5G64200), *FLC* (AT5G10140), and *FLK* (AT3G04610).

## Supplementary Material

kiaf335_Supplementary_Data

## Data Availability

The data supporting the findings of this study are available in the article and in its supplementary material.

## Supplementary data

The following materials are available in the online version of this article.

**Supplementary Figure S1.** Summary of SPF30 transcript isoforms.

**Supplementary Figure S2.** RT-qPCR analysis of SPF30 transcript isoforms in different Arabidopsis tissues and under cycloheximide (CHX) treatment.

**Supplementary Figure S3.** SPF30.2 was not detected in Arabidopsis by RT-PCR.

**Supplementary Figure S4.** Co-expression of SPF30.1 with spliceosomal proteins.

**Supplementary Figure S5.** RT-qPCR analysis of the spf30 mutant.

**Supplementary Figure S6.** Expression of flowering time-related genes in spf30.

**Supplementary Figure S7.** FLC transcripts with retained introns were subjected to nonsense-mediated mRNA decay (NMD) degradation.

**Supplementary Figure S8.** Retention of FLC intron 5 and intron 6 was rescued by introduction of the genomic sequence of SPF30 (gSPF30).

**Supplementary Figure S9.** RT-qPCR analysis of FLC-like genes in wild-type (WT) and spf30 seedlings.

**Supplementary Figure S10.** RT-qPCR analysis of intron retention in FLK, SOC1 and CO in spf30 and wild-type (WT) seedlings.

**Supplementary Figure S11.** RNA immunoprecipitation (RIP) analysis of SPF30 and FLC RNA.

**Supplementary Figure S12.** Microscale thermophoresis (MST) analysis of the binding affinity between FLC and SPF30.1, U2AF65a, and U2AF35b in vitro.

**Supplementary Figure S13.** RT-qPCR analysis of intron 1 retention of FLK in wild-type (WT), spf30 and spf30 complemented with the coding sequence (CDS) of individual SPF30 isoforms.

**Supplementary Figure S14.** The second intron in the full-length SPF30.3 transcript (*SPF30.3FL*) can be further spliced in Nicotiana.

**Supplementary Table S1.** Primers used in this study.

**Supplementary Data Set 1.** Sequences of SPF30 splice isoforms.

**Supplementary Data Set 2.** Sequences of splice isoforms identified in SPF30 intron 2 by Sanger sequencing.

## Dive Curated Terms

The following phenotypic, genotypic, and functional terms are of significance to the work described in this paper:

U2AF35B Gramene: AT5G42820
U2AF35B Araport: AT5G42820

U1-70k Gramene: AT3G50670
U1-70k Araport: AT3G50670

U1a Gramene: AT2G47580
U1a Araport: AT2G47580

rosette leaf AmiGo: PO:0000014

MES CHEBI: CHEBI:39010

FLC Gramene: AT5G10140
FLC Araport: AT5G10140

SPF30 Gramene: AT2G02570
SPF30 Araport: AT2G02570
