# Supplementary material for: The role of Arabidopsis Splicing Factor 30 in floral transition and the implications of its alternative splicing
Source: Plant Physiol. 2025 Jul 31;198(4):kiaf335. doi: 10.1093/plphys/kiaf335 (PMC12393150; doi:10.1093/plphys/kiaf335)
Supplement: kiaf335_Supplementary_Data [file kiaf335_supplementary_data.zip › 25-07-23 Supplementary data.pdf]

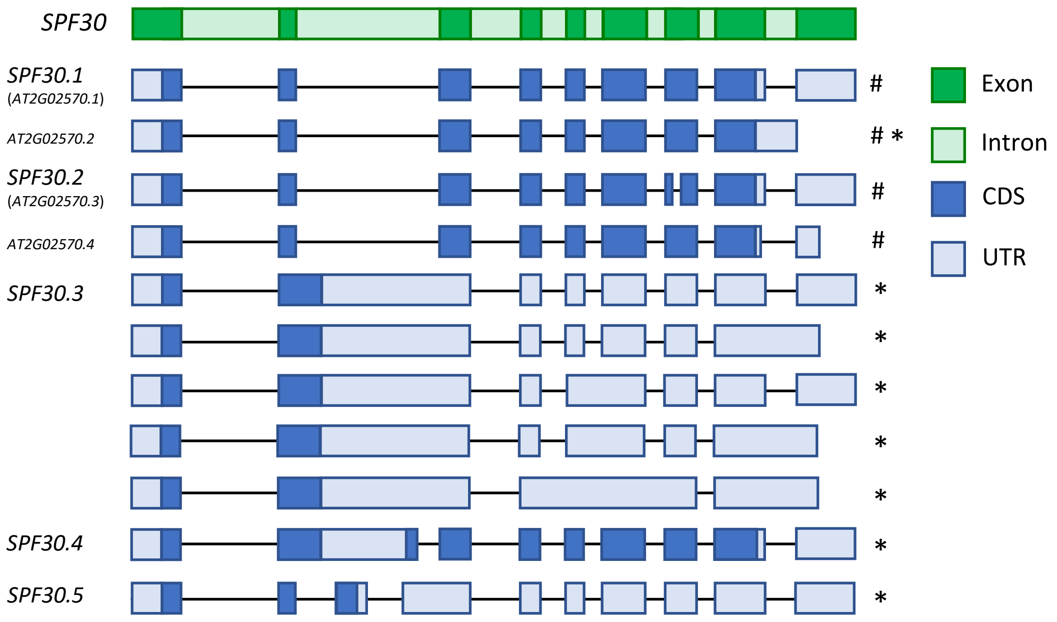

Supplementary Figure S1 Summary of *SPF30* transcript isoforms. The gene model of *SPF30* is presented at the top and structures of various transcripts isoforms are listed below. # indicates those that are annotated by the TAIR10 database. * indicates those that were identified in our iso RNA-sequencing (Zhu et al., 2017). CDS: coding sequence. UTR: untranslated region.

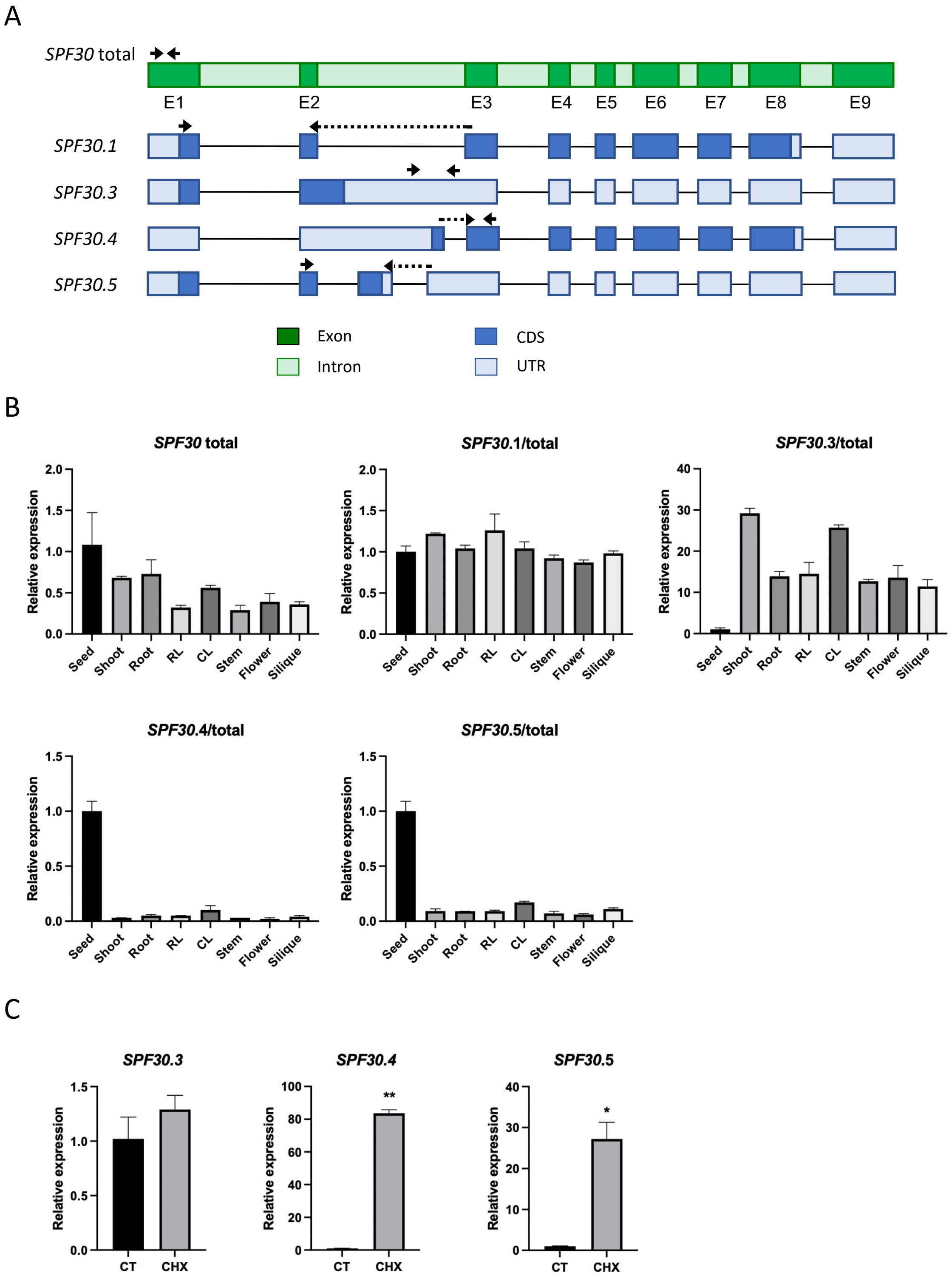

Supplementary Figure S2 RT-qPCR analysis of *SPF30* transcript isoforms in different Arabidopsis tissues and under cycloheximide (CHX) treatment. A, Schematic view of *SPF30* splice isoforms. Arrows indicate the positions of primers used in B and C. CDS: coding sequence; UTR: untranslated region. B, RT-qPCR analysis of *SPF30* transcript isoforms in different Arabidopsis tissues. RL: rosette leaf; CL: cauline leaf. C, RT-qPCR analysis of *SPF30* transcript isoforms in seedlings treated with 100 µg/ml CHX for 3 h at room temperature. Seedlings treated with water served as control (CT). B and C, Error bars represent SE from two biological replicates, and asterisks indicate significant differences (*, $p < 0.05$; **, $p < 0.01$; Student's t-test).

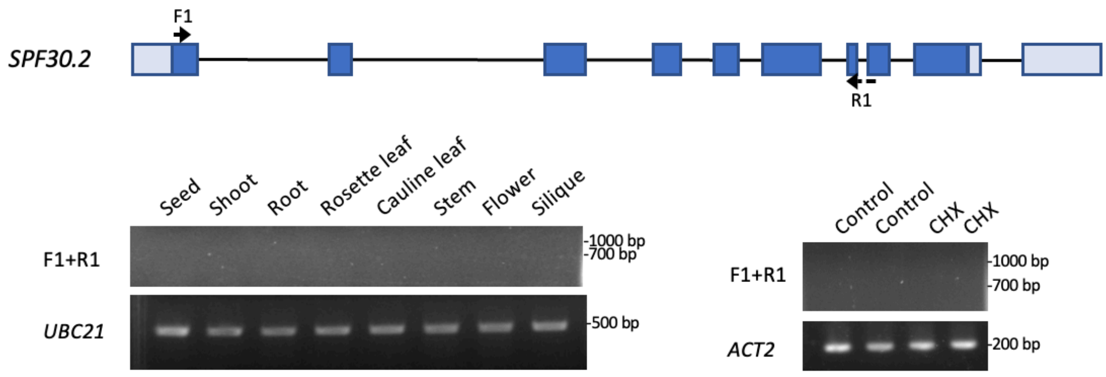

Supplementary Figure S3 *SPF30.2* was not detected in Arabidopsis by RT-PCR. Schematic view of the *SPF30.2* mRNA is shown on top. Dark blue boxes indicate coding regions (CDS) and light blue boxes indicate untranslated regions (UTR). When using the primer pair F1 and R1, no bands were detected after PCR was run for 40 cycles. For cycloheximide (CHX) treatment, seedlings were treated with 100 µg/ml CHX or water (as control) for 3 h at room temperature.

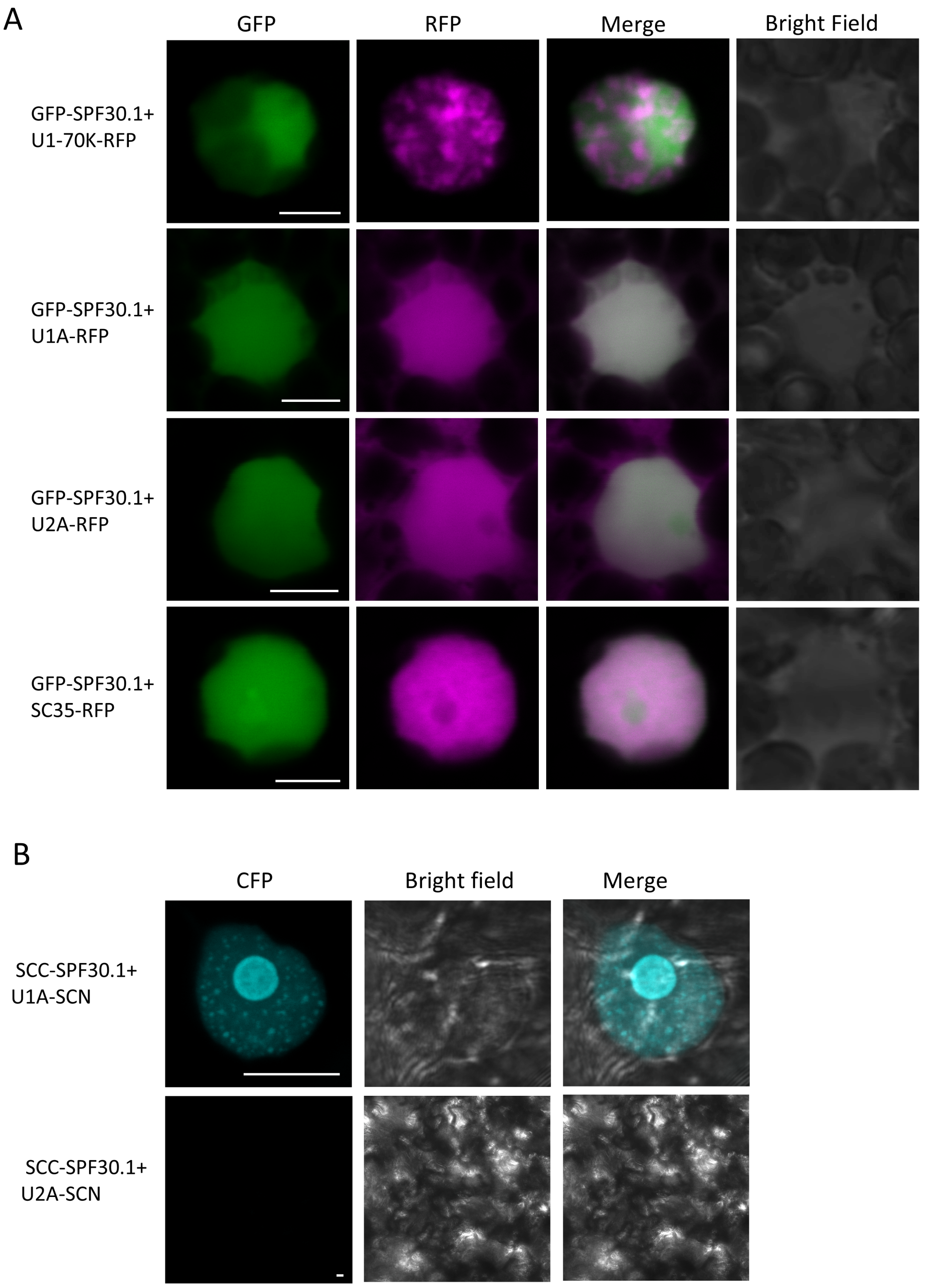

Supplementary Figure S4 Co-expression of SPF30.1 with spliceosomal proteins. A, GFP-tagged SPF30.1 was co-expressed with RFP-tagged U1-70K, U1A, U2A and SC35 in Arabidopsis protoplasts. Bars = 5 μm. B, bimolecular fluorescence complementation (BIFC) assay of SPF30.1 with U1A and U2A in Nicotiana leaf cells. Bars = 10 μm.

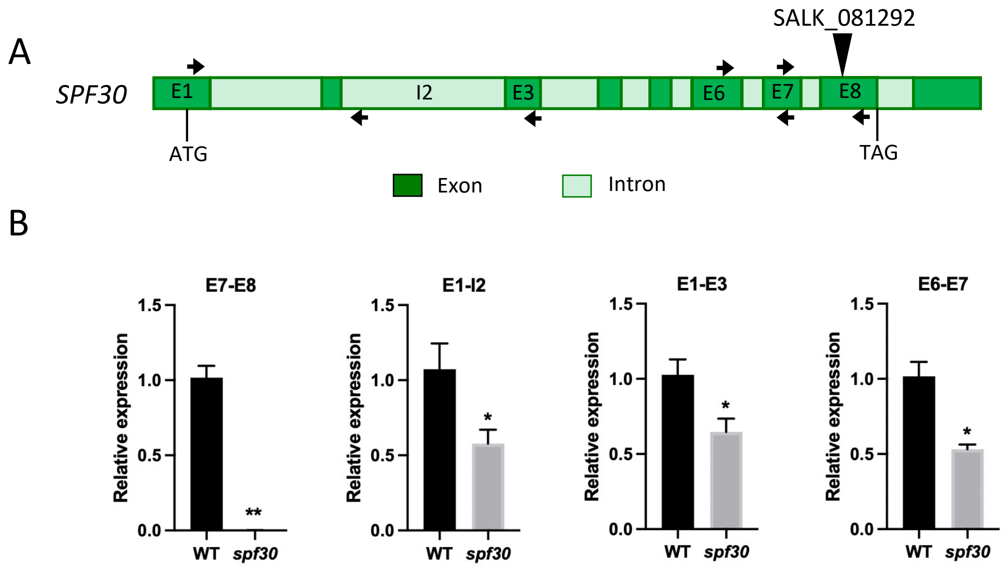

Supplementary Figure S5 RT-qPCR analysis of the *spf30* mutant. A, Schematic view of the SALK_081292 (*spf30*) mutant line. B, RT-qPCR analysis of the 12-day-old *spf30* and wild-type (WT) seedlings. Primers used were indicated by arrows in A. Error bars represent SE from three biological replicates. Asterisks indicate significant differences (*, $p < 0.05$; **, $p < 0.01$; Student's t-test).

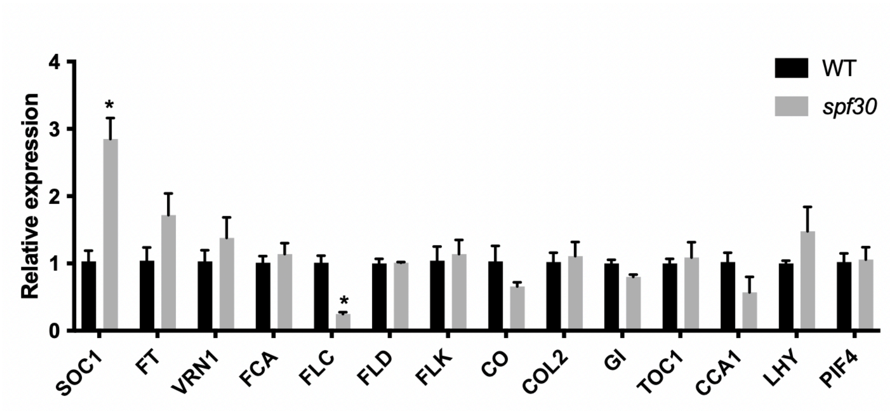

Supplementary Figure S6 Expression of flowering time-related genes in *spf30*. Expression of representative flowering time-related genes was analyzed by RT-qPCR in 12-day-old *spf30* and wild-type (WT) seedlings grown on MS medium. Error bars represent SE from two or three biological replicates. Asterisks indicate significant differences (*, $p < 0.05$; Student's t-test).

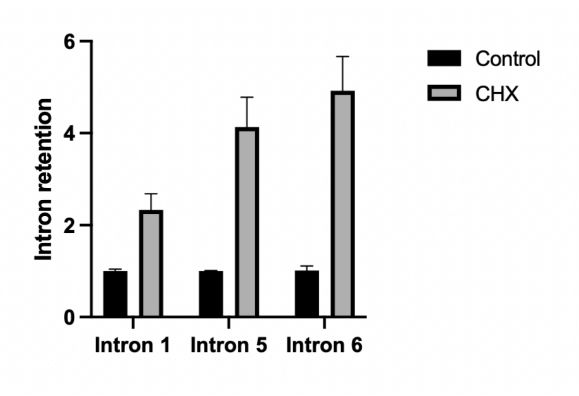

Supplementary Figure S7 *FLC* transcripts with retained introns are subjected to nonsense-mediated mRNA decay (NMD). The retention levels of *FLC* introns were analyzed by RT-qPCR in 12-day-old Arabidopsis seedlings treated with cycloheximide (CHX). Seedlings treated with water served as controls. Intron retention was calculated as the ratio of unspliced to spliced *FLC* transcripts. Error bars represent SE from two biological replicates.

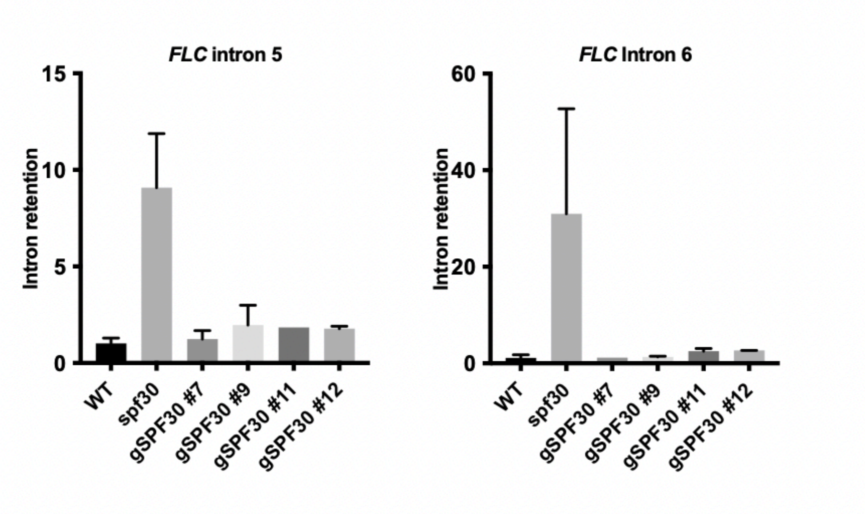

Supplementary Figure S8 Retention of *FLC* intron 5 and intron 6 was rescued by introduction of the genomic sequence of *SPF30* (g*SPF30*). Intron retention of *FLC* intron 5 and intron 6 was analyzed in wild-type (WT), *spf30* and *spf30* complemented with g*SPF30*. Error bars represent SE from two biological replicates. # indicates independent transgenic lines.

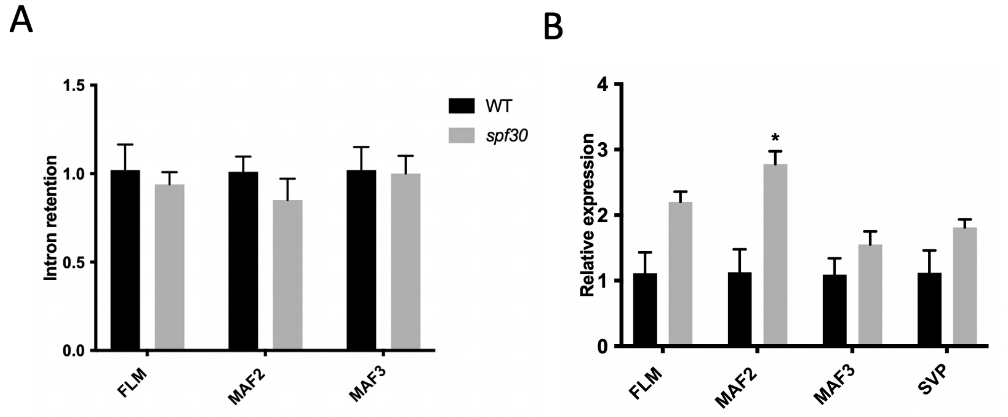

Supplementary Figure S9 RT-qPCR analysis of *FLC*-like genes in wild-type (WT) and *spf30* seedlings. A, Retention of the first intron of *FLC*-like genes. (B) Transcript levels of *FLC*-like genes. Error bars represent SE from two biological replicates. Asterisks indicate significant differences (*, p < 0.05; Student's t-test).

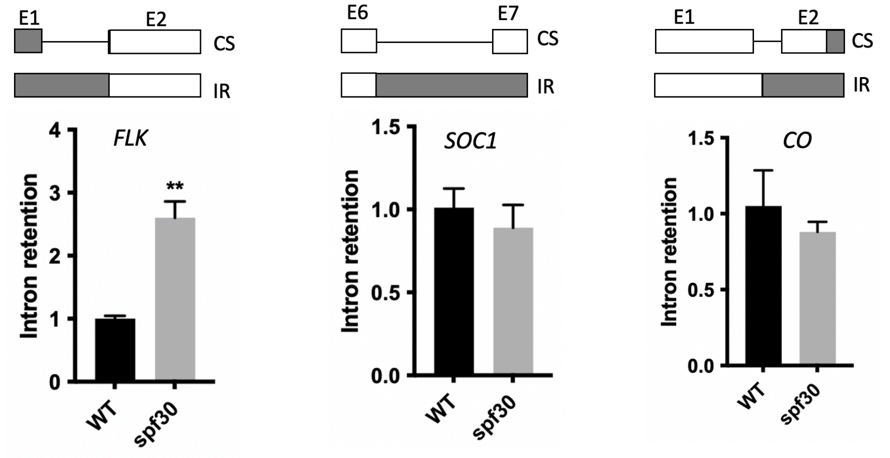

Supplementary Figure S10 RT-qPCR analysis of intron retention in *FLK*, *SOC1* and *CO* in *spf30* and wild-type (WT) seedlings. Exons are represented by boxes and introns are indicated by lines. White boxes indicate coding regions (CDS); gray boxes indicate untranslated regions (UTR). Intron retention (IR) was calculated as the ratio of unspliced (IR) to spliced (CS: constitutive splicing) transcripts. Error bars represent SE from three biological replicates. Asterisks indicate significant differences (**, p < 0.01; Student's t-test).

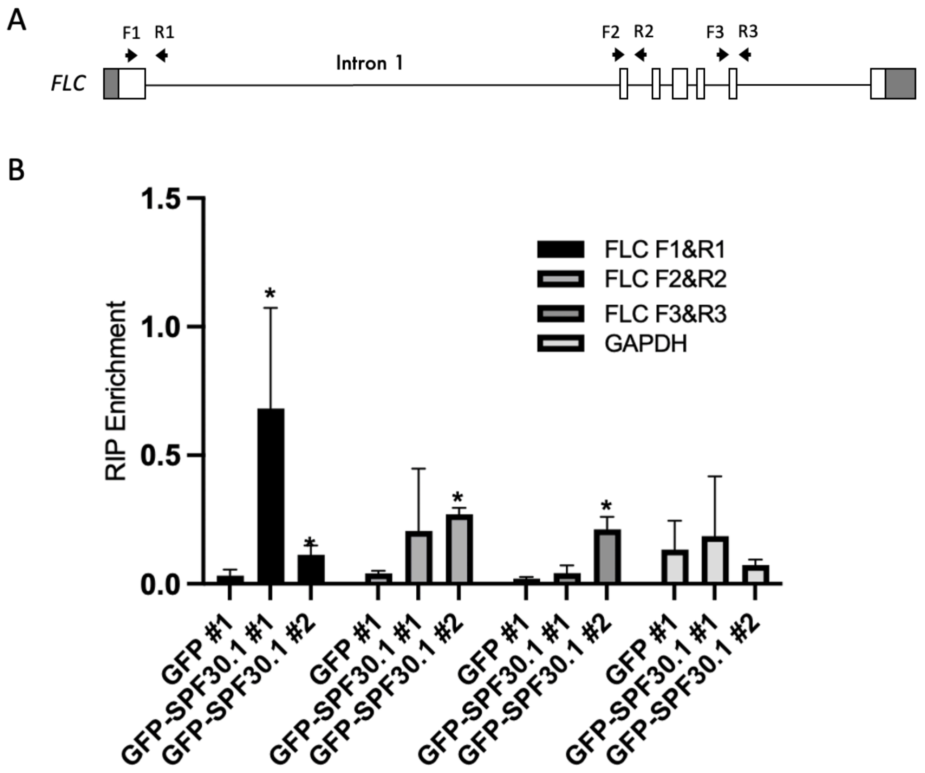

Supplementary Figure S11 RNA immunoprecipitation (RIP) analysis of SPF30 and *FLC* RNA. A, Schematic view of *FLC* gene structure. Exons are represented by boxes and introns are indicated by lines. White boxes indicate coding regions (CDS) and gray boxes indicate untranslated regions (UTR). Arrows indicate the primers used for RT-qPCR analysis in RIP analysis. B, Arabidopsis transgenic lines stably expressing GFP or GFP-tagged SPF30 proteins were used for RIP analysis. The immunoprecipitated *FLC* was analysed by RT-qPCR, with *GAPDH* serving as control. Enrichment was measured as the level of targeted fragments in the immunoprecipitated RNA relative to the input. The data represents the mean ± SD from three technical controls. Asterisks indicate significant differences in comparison to GFP (*, $p < 0.05$; Student's t-test). # indicates independent transgenic lines.

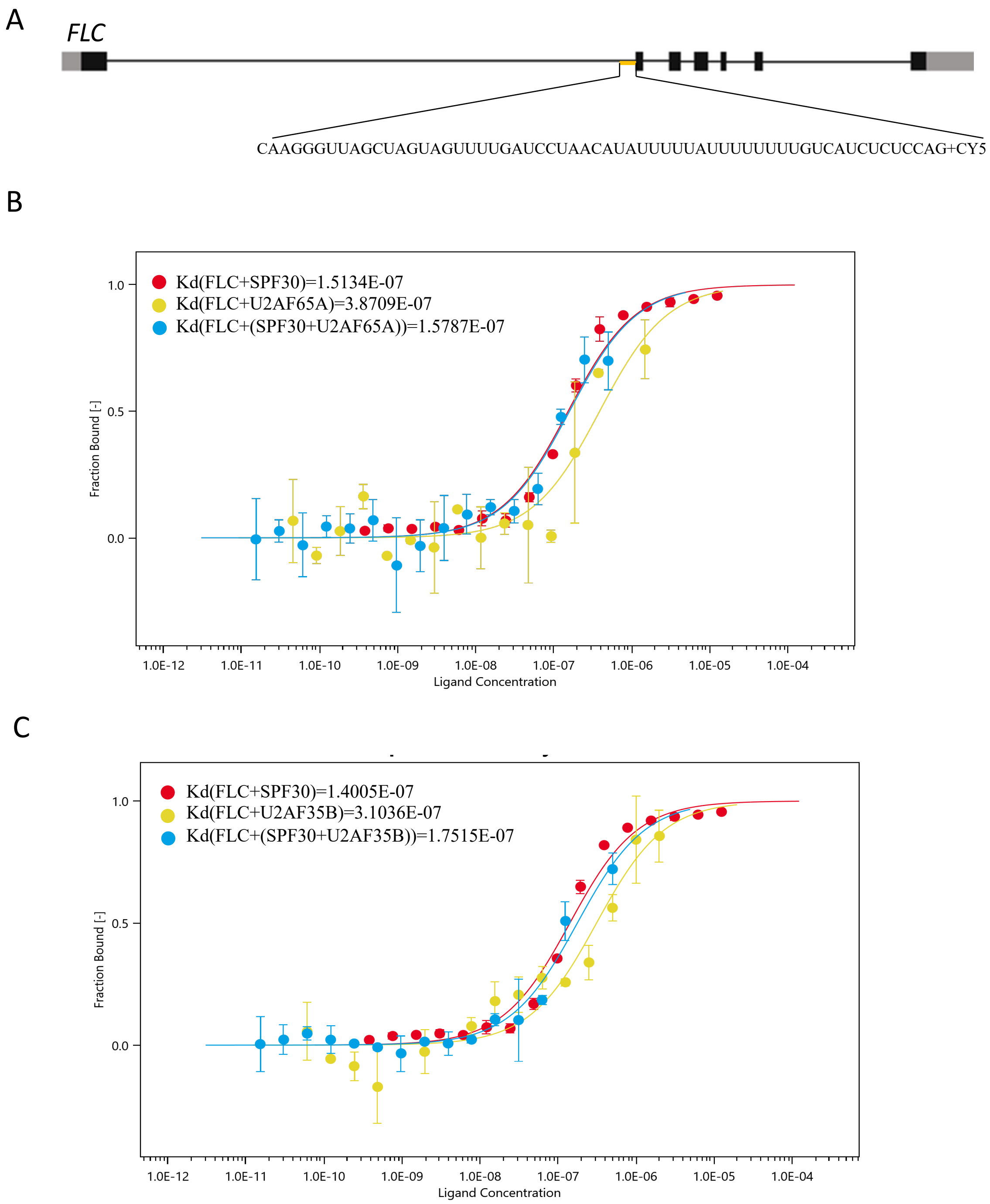

Supplementary Figure S12 Microscale thermophoresis (MST) analysis of the binding affinity between *FLC* and SPF30.1, U2AF65a, and U2AF35b in vitro. A, Schematic view of the fluorescently labelled *FLC* RNA fragment synthesized as target. Exons are represented by boxes and introns are indicated by lines. Black boxes indicate coding regions (CDS) and gray boxes indicate untranslated regions (UTR). B and C, Binding curves and dissociation constant (K_d) of *FLC* with SPF30.1, U2AF65a, U2AF35b, or with SPF30 pre-incubated with U2AF65a or U2AF35b with equimolar amounts. Lower K_d values indicate higher affinity between the molecules tested. The data represents the mean ± SD from two independent experiments.

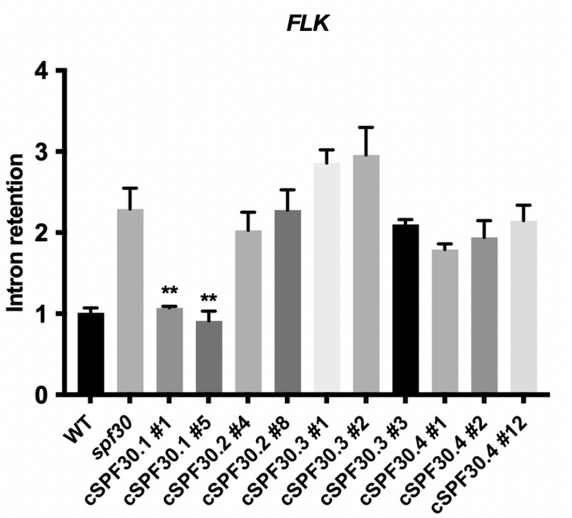

Supplementary Figure S13 RT-qPCR analysis of intron 1 retention of *FLK* in wild-type (WT), *spf30* and *spf30* complemented with the coding sequence (CDS) of individual *SPF30* isoforms. Error bars represent SE from three biological replicates. Asterisks indicate significant differences in comparison to *spf30* (**, p<0.01; Student's t-test).

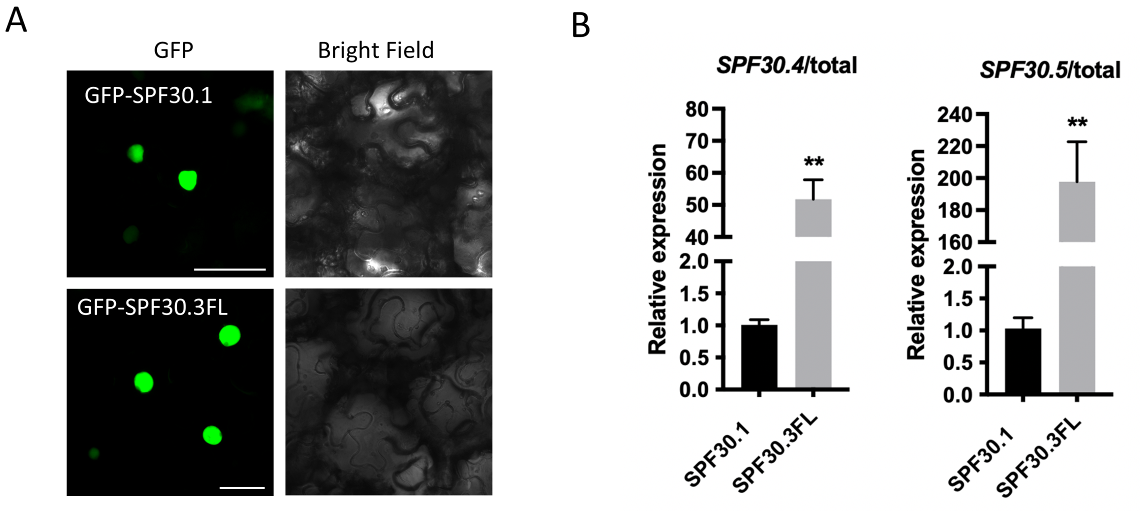

Supplementary Figure S14 The second intron in the full-length *SPF30.3* transcript (*SPF30.3FL*) can be further spliced in Nicotiana. A, Subcellular localization of GFP-tagged SPF30.1 and SPF30.3FL in Nicotiana leaf cells. Bars = 50 µm. B, RT-qPCR analysis of GFP-tagged *SPF30.1* and *SPF30.3FL* transcripts expressed in Nicotiana leaf cells. The characteristic alternative splicing (AS) patterns of *SPF30.4* or *SPF30.5* were analyzed in comparison to the total amount of heterologously expressed total *SPF30* transcripts. Error bars represent SE from three biological replicates. Asterisks indicate significant differences (**, P<0.01; Student's t-test).

Supplementary Table S1 Primers used in this study

Primer used for *SPF30* (AT2G02570) and other splicing-related genes

Primer name	Primer sequences	Description
SPF30.1-F	**CACC**ATGGTAGGAGGAGTAGAAGAA	Detection of *SPF30.1*;<br>Cloning of *SPF30.1*/*.2* CDS and *SPF30.3FL;* Mutant validation
SPF30.1-R	TCATTCATCAGTGCCCTCAGCAT	
SPF30.2-F	**CACC**ATGGTAGGAGGAGTAGAAGAA	Detection of *SPF30.2*
SPF30.2-R	TTCTGCACAACATGTATCTT	
SPF30.3-F	**CACC**ATGGTAGGAGGAGTAGAAGAA	Detection of *SPF30.3;*<br>Cloning of *SPF30.3* CDS
SPF30.3-R	TCAATGAATTTCACTCAAACTAAGGG	
SPF30.4-F	GTTTGATCCCTGAA GTGAT	Detection of *SPF30.4*
SPF30.4-R	TCATTCATCAGTGCCCTCAGCAT	
SPF30.4-Clone-F	**CACC**ATGATGGCCAGCCTGCTTCTATCTCCTGATTT<br>CTCGTGTTTGATCCCTGAAGTGATTGCATTGACAGAG	Cloning of *SPF30.4* CDS
SPF30.4-Clone-R	TCATTCATCAGTGCCCTCAGCAT	
SPF30.5-F	**CACC**ATGGTAGGAGGAGTAGAAGAA	Detection of *SPF30.5*
SPF30.5-R	TGACCCATATAAGCACACCTTTC	
*SPF30*ΔCter-F	**CACC**ATGGTAGGAGGAGTAGAAGAA	Cloning of *SPF30*ΔCter
*SPF30*ΔCter-R	TCA TTCGACAATAGCATTTTGCTC	
SPF30.1-BIFC-F	GGACTAGTATGGTAGGAGGAGTAGAAGAA	Cloning of *SPF30.1* CDS with SpeI and KpnI sites
SPF30.1-BIFC-R	GGGGTACCTCATTCATCAGTGCCCTCAGCAT	
SPF30-promoter-F	**CACC**CTTTGTAGACCCGTGAGCCAG	Cloning of *SPF30* promoter
SPF30-promoter-R	CTGTTCGAGTTGTTCTTTGTAGGTAG	
SPF30-AS-F1	GTTAGGCAGCTTTTGTCAGAGG	Detection of AS events in *SPF30* intron 2
SPF30-AS-R1	TGACCCATATAAGCACACCTTTC	
SPF30-AS-F2	GTTTTGCATACGGTGCTCTT	
SPF30-AS-R2	GGGATCAAACACGAGAAATCAGG	
SPF30-AS-F3	AGCCTGCTTCTATCTCCTGA	
SPF30-AS-R3	CCAAGCACCCTCTAAATCGG	
*SPF30*FL-GFP-F	CGCGCCAAGCTATCAAACAA	Detection of *SPF30FL* expressed with *GFP*
*SPF30*FL-R	CCAAGCACCCTCTAAATCGG	
SPF30.1-qCPR-F	ATGGTAGGAGGAGTAGAAGAATTGAG	Detection of *SPF30.1* in qPCR
SPF30.1-qPCR-R	CAATGCAATCACCTCTTTAAGCTCC	
SPF30.3-qCPR-F	AACTCCGGCAGTGGAGTTTG	Detection of *SPF30.3* in qPCR
SPF30.3-qPCR-R	GGTGCAAAAACAAAGTTTCAGAG	
SPF30.4-qCPR-F	GTTTGATCCCTGAA GTGAT	Detection of *SPF30.4* in qPCR
SPF30.4-qPCR-R	CCAAGCACCCTCTAAATCGG	
SPF30.5-qCPR-F	GTTAGGCAGCTTTTGTCAGAGG	Detection of *SPF30.5* in qPCR
SPF30.5-qPCR-R	TGACCCATATAAGCACACCTTTC	
SPF30-total-5'-F	GCGACAAAGAACCATCGGAGA	Detection of *SPF30* total transcripts in qPCR in Fig. S2
SPF30-total-5'-R	CCCAAACTCAAAACTTCGATCTCA	
SPF30-total-3'-F	TGGCAACAGTTTCAGACGAC	Detection of *SPF30* total transcripts in qPCR in Fig. 6, Fig. 8, Fig. 9 and Fig. S14
SPF30-total-3'-R	AGTGCCCTCAGCATTACCAG	
SPF30-totalAS-F	AGCCTGCTTCTATCTCCTGA	

SPF30-totalAS-R	CCAAGCACCCTCTAAATCGG	
SPF30-E1-F	ATGGTAGGAGGAGTAGAAGAATTGAG	Analysis of the spf30 mutant
SPF30-I2-R	TCAATGAATTTCACTCAAACTAAGGG	
SPF30-E3-R	TCTAAATCGGGTGAGCCAGGA	
SPF30 E6-F	TGAGAAAGCTGCGAGTTCTGAT	
SPF30-E7-R	CTTTTTAGTTTTGGCTTTAGTCG	
SPF30-E7-F	TGGCAACAGTTTCAGACGAC	
SPF30-E8-R	AGTGCCCTCAGCATTACCAG	
SPF30-pET-F	gacaaggccatggctgatatcATGGTAGGAGGAGTAGAAGAATTGAGT	One step cloning of *SPF30* into pET30-a based on homologous recombination
SPF30-pET-R	gtggtggtggtggtgctcgagTTCATCAGTGCCCTCAGCATT	
U2AF65a-RFP-F	CCCAAGCTTATGGACTACAAAGACGATGACGACAAA TCTGAATTCGAAGATCACGA	Cloning of *U2AF65a* (AT4G36690) CDS fused to a Flag tag with HindIII and BamHI sites
U2AF65a-RFP-R	CGGGATCCAGGCTCCATAATCACCCTGTTC	
U2AF65a-BIFC-F	GGACTAGTATGTCTGAATTCGAAGATCACGA	Cloning of *U2AF65a* with SpeI and KpnI sites
U2AF65a-BIFC-R	GGGGTACCGGCTCCATAATCACCCTGTTC	
U2AF65a-pET-F	gacaaggccatggctgatatcATGTCTGAATTCGAAGATCACGAG	One step cloning of *U2AF65a* into pET30-a based on homologous recombination
U2AF65a-pET-R	gtggtggtggtggtgctcgagGGCTCCATAATCACCCTGTTCA	
U2AF35b-RFP-F	CCCAAGCTTATGGCAGAGCATTTAGCTTC	Cloning of *U2AF35b* (AT5G42820) with HindIII and KpnI sites
U2AF35b-RFP-R	GGGGTACCAAACTCCCTCATCACGTTCTC	
U2AF35b-pET-F	gacaaggccatggctgatatcATGGCAGAGCATTTAGCTTCAAT	One step cloning of U2AF35b into pET30-a based on homologous recombination
U2AF35b-pET-R	gtggtggtggtggtgctcgagAACTCCCTCATCACGTTCTCGG	
U2AF35b-BIFC-F	GGACTAGTATGGCAGAGCATTTAGCTTC	Cloning of *U2AF35b* with SpeI and KpnI sites
U2AF35b-BIFC-R	GGGGTACCAACTCCCTCATCACGTTCTC	
RDM16-RFP-F	CG GAATTCATGGATAAGGAGAGATATTCCAGG	Cloning of *RDM16* (AT1G28060) with EcoRI and BamHI sites
RDM16-RFP-R	CG GGATCCAGTCGTCTGAGTAATTGACAGCG	
RDM16-BIFC-F	GGACTAGTATGGATAAGGAGAGATATTCCAGG	Cloning of *RDM16* with SpeI and KpnI sites
RDM16-BIFC-R	GGGGTACCGTCGTCTGAGTAATTGACAGCG	
U1-70K-RFP-F	GAAGATCTATGGGAGACTCCGGCGAT	Cloning of *U1-70K* (AT3G50670) CDS with BglII and KpnI sites
U1-70K-RFP-R	GGGGTACCAACGAACATACTCTCGCGAT	
U1-70K-BIFC-F	GGACTAGTATGGGAGACTCCGGCGAT	Cloning of *U1-70K* with SpeI and KpnI sites
U1-70K-BIFC-R	GGGGTACCACGAACATACTCTCGCGAT	
U1A-RFP-F	CGGAATTCATGGACTACAAAGACGATGACGACAAA GAGATGCAAGAGGCTAAT	Cloning of *U1A* (AT2G47580) CDS fused to a Flag tag with EcoRI and BamHI sites
U1A-RFP-R	CGGGATCCATTTCTTGGCATACGTGATGAG	
U1A-BIFC-F	GGACTAGTATGGAGATGCAAGAGGCTAAT	Cloning of *U1A* with SpeI and KpnI sites
U1A-BIFC-R	GGGGTACCTTTCTTGGCATACGTGATGAG	
U2A-RFP-F	GAAGATCTATGGTGAAGCTCACGGCTGATTTG	Cloning of *U2A* (AT1G09760) with BglII and KpnI sites
U2A-RFP-R	GGGGTACCATTCCTCCATGGGAGCAGAGTC	
U2A-BIFC-F	GGACTAGTATGGTGAAGCTCACGGCTGATTTG	Cloning of *U2A* with SpeI and KpnI sites
U2A-BIFC-R	GGGGTACCTTCCTCCATGGGAGCAGAGTC	
SC35-RFP-F	CGGAATTCATGTCGCACTTCGGAAGGT	Cloning of *SC35* (AT5G64200) with EcoRI and BamHI sites
SC35-RFP-R	CGGGATCCATTCCGCAGCATAAGGAGAT	

Note: The 5' CACC overhangs used for cloning into the pENTR/D-TOPO vector are in bold. Restriction enzyme digestion sites are underlined. The homologous sequences used for one-step cloning are shown in lower case.

Primer used for amplification of flowering-related genes and internal reference genes

Gene ID	Primer name	Primer sequences
AT1G65480	*FT*-qPCR-F	ACAACTGGAACAACCTTTGGCAATG
	FT-qPCR-R	ACTGTTTGCCTGCCAAGCTGTC
AT3G18990	*VRN1*-qPCR-F	CCAACGCCAACCCCAAAAAT
	VRN1-qPCR-R	GGTCGCAGAACCACTCTGAA
AT4G16280	*FCA*-qPCR-F	CGTGCAACTGCTCCTCAAAC
	FCA-qPCR-R	TGGGAACGAGGGGGAAATTG
AT3G10390	*FLD*-qPCR-F	CTCTCTTGATCGCGTTGGTT
	FLD-qPCR-R	GGACATTTATTCCTTGAGGTTCA
AT3G02380	*COL2*-qPCR-F	CATGGCCCCAAGTGAGACTG
	COL2-qPCR-R	CCACGAAGCAACCTCTCGAT
AT1G22770	*GI*-qPCR-F	GCCCAAGTAGTGAGAATGACT
	GI-qPCR-R	CACCACTACACCATCGGAAA
AT5G61380	*TOC1*-qPCR-F	ATCTTCGCAGAATCCCTGTGATA
	TOC1-qPCR-R	GCACCTAGCTTCAAGCACTTTACA
AT2G46830	*CCA1*-qPCR-F	CCTTTTACAAACACCGGCTCTT
	CCA1-qPCR-R	AATCGGGAGGCCAAAATGA
AT1G01060	*LHY*-qPCR-F	TCAGGGAGTGACGCAGAAAC
	LHY-qPCR-R	GCAAAGAGAGCCTGAAACGC
AT2G43010	*PIF4*-qPCR-F	AAACCGAAGGAGTCGAGCAG
	PIF4-qPCR-R	AGGTAACTGTACCGGGCTCT
AT5G10140	*FLC*-Intron1-IR-F1	TTCTCCAAACGTCGCAACGGTCTC
	FLC-Intron1-IR-R1	CTCAGAAAAGTAAAAGAGCACAAAACAG
	FLC-Intron1-CS-F1	TTCTCCAAACGTCGCAACGGTCTC
	FLC-Intron1-CS-R1s	CATGCTGTTTCCCATATCGATCAAG
	FLC-Intron5-IR-F5	AACTCATGTTGAAGCTTGTTGAG
	FLC-Intron5-IR-R5	AATATGTGTGCAAGCTCGTTAAG
	FLC-Intron5-CS-F5s	GAATCTTAAAGAAAAGGAGAAAATGCTG
	FLC-Intron5-CS-R5s	CAGCTTCTGCTCCCACATGA
	FLC-Intron6-IR-F6	GCTTCCAAACTTAAAAGCTTAAACA
	FLC-Intron6-IR-RT	TCACACACAAAGTCTCTTGG
	FLC-Intron6-CS-F6s	ATGCTGAAAGAAGAGAACCAGG
	FLC-Intron6-CS-RT	TCACACACAAAGTCTCTTGG
	FLC-total-FT	CAGAAGCTGAGATGGAGATG
	FLC-total-RT	TCACACACAAAGTCTCTTGG
AT5G10140	*FLC*-RIP-F1	GCCGACAAGTCACCTTCTC
	FLC-RIP-R1	CCCAGGTAAGGAAAAGGCG
	FLC-RIP-F2	TGTCATCTCTCCAGCCTGGTCAAG
	FLC-RIP-R2	AATGCATGTGGAGCCACCACCTCAT
	FLC-RIP-F3	CTTGATCGGTAATTGCTGAACA
	FLC-RIP-R3	CATTGGACACACAACACGCAG
AT3G04610	*FLK*-total-F	TTCGTTGCACACAATGGAGA
	FLK-total-R	TGGTTGGTGAACAAGGGTCT
	FLK-IR-F	CGGGACTAATGTGTGTTATCTGG
	FLK-IR-R	TGGTTGGTGAACAAGGGTCT
	FLK-CS-F	CCACTGATACCACCGCTCTAG
	FLK-CS-R	TGGTTGGTGAACAAGGGTCT
AT5G65050	*MAF2*-total-F	CAAGCTTGAAGAATCAAATGTCG

	MAF2-total-R	TCGAGCTGTTCCTCCAGAGA
	MAF2-IR-F	GGCTCCGGAAAACTCTACAA
	MAF2-IR-R	CCTCATACAGAAAAAGGGGGAGA
	MAF2-CS-F	GGCTCCGGAAAACTCTACAA
	MAF2-CS-R	GAGTGGCAGATAATTCCGAG
AT1G77080	*FLM*-total-F	GCAACAAAATACCGGAGACT
	FLM-total-R	CCGTACATTCAGACACAAAC
	FLM-IR-F	GATCAAGCGAATCGAGAACA
	FLM-IR-R	GCACAAAAATCGAGTGAGAC
	FLM-CS-F	GATCAAGCGAATCGAGAACA
	FLM-CS-R	AGGCTCTAAGTTCATCAGCA
	MAF3-total-F	ATGGCTCCGGCAACAAAGTA
	MAF3-total-R	ATGGTGAAAGCTCAGCCGTT
AT5G65060	*MAF3*-IR-F	CAAACGACGCAAAGGTCTCA
	MAF3-IR-R	ACGCATAGGCTCTATTCACA
	MAF3-CS-F	CAAACGACGCAAAGGTCTCA
	MAF3-CS-R	GCATGATGTATTTCATAACGATCAA
AT2G22540	SVP-total-F	AACGCTGCTGTGTACGAGGAAG
	SVP-total-R	TCTCTAACCACCATACGGTAAGCC
AT2G45660	*SOC1*-qPCR-F	AGCTCTCTGAAAAGTGGGGA
	SOC1-qPCR-R	GGGCTACTCTCTTCATCACCT
	SOC1-IR-F	TAAGCCTCTTGTGCTTGCTTT
	SOC1-IR-R	TTGACCAAACTTCGCTTTCA
	SOC1-CS-F	AGCAGCTCAAGCAAAAGGAG
	SOC1-CS-R	TTGACCAAACTTCGCTTTCA
AT5G15840	*CO*-qPCR-F	CCATTAACCATAACGCATAC
	CO-qPCR-R	TACTGTCCCTTTGGGCGTTC
	CO-IR-F	ACCAGGGGGATTGAGAATGT
	CO-IR-R	TACTGTCCCTTTGGGCGTTC
	CO-CS-F	CCATTAACCATAACGCATAC
	CO-CS-R	TACTGTCCCTTTGGGCGTTC
AT3G18780	*ACT2*-F	TGTGCCAATCTACGAGGGT
	ACT2-R	GCTGGTCTTTGAGGTTTCC
AT5G62690	*TUB2*-qPCR-F	ATCCGTGAAGAGTACCCAGAT
	TUB2-qPCR-R	AAGAACCATGCACTCATCAGC
AT5G25760	*UBC21*-F	GGCATCAAGAGCGCGACTG
	UBC21-R	TTCTTAGGCATAGCGGCGA
AT3G26650	*GAPDH*-F	TGGTTGATCTCGTTGTGCAGGTCTC
	GAPDH-R	GTCAGCCAAGTCAACAACTCTCTG

Note: IR stands for intron retention. CS stands for constitutively spliced and corresponds to transcripts without the introns.

Supplementary Dataset S1 Sequences of *SPF30* splice isoforms

***SPF30.1* CDS (903 bp)**
ATGGTAGGAGGAGTAGAAGAATTGAGTATTGAACAGTTAGCTTCGAGTATCTCTACCTACAAAGAACAACTCG
AACAGGTTAGGCAGCTTTTGTCAGAGGATCCTAGGAACTCGGAATATGCAGACATGGAAAAGGAGCTTAAAG
AGGTGATTGCATTGACAGAGGAAGTTCTTGCAACTGCAAAGCAAAATGAGATTTCTCTATCAGATGCCGGAGT
TAGTGCTGAAGCAACTCCTGGCTCACCCGATTTAGAGGGTGCTTGGGAAAAGACGGGTTTAAGGAATGACCCA
ATCCATGAGGGTAAGTTCCCTGTTGGAACTAAAGTTCAAGCTGTCTTTAGTGACGATGGCGAGTGGTATGATG
CGACCATTGAGGCACATACTGCAAATGGCTATTTTGTTGCTTATGATGAGTGGGGAAACAAGGAAGAGGTGG
ATCCAGATAATGTGAGGCCAATCGAGCAAAATGCTATTGTCGAAGCTGAGAGATTAGCTCAAGCTACCAAAA
ATGCTCTCAAAAGAAAGATTGAGAAAGCTGCGAGTTCTGATTATCAGACAAAAACTCTACCAGCAAAGCTCA
AAATCGATCCTAATGATCCCGAGGATGTAAAAATAGCTAAGCGTAAGAAGATACATGCTTTCAAATCCAAGG
CAAGGTTTGAGCAACTCGAGGTTGTGCAGAACAAGAAACAGAATGATTGGCAACAGTTTCAGACGACTAAAG
CCAAAACTAAAAAGGTAGGGTTCTTCACAGGGAGGAAGAAAGAGAGTATATTTAAATCACCTGAGGATCCAT
TTGGAAAAGTGGGTGTGACTGGAAGTGGGAAAGGTTTGACAGATTTCCAAAAGCGAGAGAAGCATCTTCATC
TCAAGTCTGGTAATGCTGAGGGCACTGATGAATGA

***SPF30.2* CDS (867 bp)**
ATGGTAGGAGGAGTAGAAGAATTGAGTATTGAACAGTTAGCTTCGAGTATCTCTACCTACAAAGAACAACTCG
AACAGGTTAGGCAGCTTTTGTCAGAGGATCCTAGGAACTCGGAATATGCAGACATGGAAAAGGAGCTTAAAG
AGGTGATTGCATTGACAGAGGAAGTTCTTGCAACTGCAAAGCAAAATGAGATTTCTCTATCAGATGCCGGAGT
TAGTGCTGAAGCAACTCCTGGCTCACCCGATTTAGAGGGTGCTTGGGAAAAGACGGGTTTAAGGAATGACCCA
ATCCATGAGGGTAAGTTCCCTGTTGGAACTAAAGTTCAAGCTGTCTTTAGTGACGATGGCGAGTGGTATGATG
CGACCATTGAGGCACATACTGCAAATGGCTATTTTGTTGCTTATGATGAGTGGGGAAACAAGGAAGAGGTGG
ATCCAGATAATGTGAGGCCAATCGAGCAAAATGCTATTGTCGAAGCTGAGAGATTAGCTCAAGCTACCAAAA
ATGCTCTCAAAAGAAAGATTGAGAAAGCTGCGAGTTCTGATTATCAGACAAAAACTCTACCAGCAAAGCTCA
AAATCGATCCTAATGATCCCGAGGATGTAAAAATAGCTAAGCGTAAGAAGATACATGTTGTGCAGAACAAGA
AACAGAATGATTGGCAACAGTTTCAGACGACTAAAGCCAAAACTAAAAAGGTAGGGTTCTTCACAGGGAGGA
AGAAAGAGAGTATATTTAAATCACCTGAGGATCCATTTGGAAAAGTGGGTGTGACTGGAAGTGGGAAAGGTT
TGACAGATTTCCAAAAGCGAGAGAAGCATCTTCATCTCAAGTCTGGTAATGCTGAGGGCACTGATGAATGA

***SPF30.3* CDS (252 bp)**
ATGGTAGGAGGAGTAGAAGAATTGAGTATTGAACAGTTAGCTTCGAGTATCTCTACCTACAAAGAACAACTCG
AACAGGTTAGGCAGCTTTTGTCAGAGGATCCTAGGAACTCGGAATATGCAGACATGGAAAAGGAGCTTAAAG
AGGTGATCAATCCCTATTTTAATCACTTATTTTGTGTGATCAAATCTTTTCATCTTTGTTGTGGATTCGTTTTATT
AGTCTCCCTTAGTTTGAGTGAAATTCATTGA

SPF30.4 CDS (807bp)
ATGATGGCCAGCCTGCTTCTATCTCCTGATTTCTCGTGTTTGATCCCTGAAGTGATTGCATTGACAGAGGAAGT
TCTTGCAACTGCAAAGCAAAATGAGATTTCTCTATCAGATGCCGGAGTTAGTGCTGAAGCAACTCCTGGCTCA
CCCGATTTAGAGGGTGCTTGGGAAAAGACGGGTTTAAGGAATGACCCAATCCATGAGGGTAAGTTCCCTGTTG
GAACTAAAGTTCAAGCTGTCTTTAGTGACGATGGCGAGTGGTATGATGCGACCATTGAGGCACATACTGCAAA
TGGCTATTTTGTTGCTTATGATGAGTGGGGAAACAAGGAAGAGGTGGATCCAGATAATGTGAGGCCAATCGA
GCAAAATGCTATTGTCGAAGCTGAGAGATTAGCTCAAGCTACCAAAAATGCTCTCAAAAGAAAGATTGAGAA
AGCTGCGAGTTCTGATTATCAGACAAAAACTCTACCAGCAAAGCTCAAAATCGATCCTAATGATCCCGAGGAT
GTAAAAATAGCTAAGCGTAAGAAGATACATGCTTTCAAATCCAAGGCAAGGTTTGAGCAACTCGAGGTTGTG
CAGAACAAGAAACAGAATGATTGGCAACAGTTTCAGACGACTAAAGCCAAAACTAAAAAGGTAGGGTTCTTC
ACAGGGAGGAAGAAAGAGAGTATATTTAAATCACCTGAGGATCCATTTGGAAAAGTGGGTGTGACTGGAAGT
GGGAAAGGTTTGACAGATTTCCAAAAGCGAGAGAAGCATCTTCATCTCAAGTCTGGTAATGCTGAGGGCACTG
ATGAATGA

SPF30.5 CDS (240 bp)
ATGGTAGGAGGAGTAGAAGAATTGAGTATTGAACAGTTAGCTTCGAGTATCTCTACCTACAAAGAACAACTCG
AACAGGTTAGGCAGCTTTTGTCAGAGGATCCTAGGAACTCGGAATATGCAGACATGGAAAAGGAGCTTAAAG
AGGTTTTGCATACGGTGCTCTTTATGTTTCTATTCCCTGCCTCTTTTGAAGGAATCTGCGTGTTGTTTGAAATAG
CAACATCATTTAACAGCTAG

***SPF30*ΔCter CDS (480 bp)**

ATGGTAGGAGGAGTAGAAGAATTGAGTATTGAACAGTTAGCTTCGAGTATCTCTACCTACAAAGAACAACTCG
AACAGGTTAGGCAGCTTTTGTCAGAGGATCCTAGGAACTCGGAATATGCAGACATGGAAAAGGAGCTTAAAG
AGGTGATTGCATTGACAGAGGAAGTTCTTGCAACTGCAAAGCAAAATGAGATTTCTCTATCAGATGCCGGAGT
TAGTGCTGAAGCAACTCCTGGCTCACCCGATTTAGAGGGTGCTTGGGAAAAGACGGGTTTAAGGAATGACCCA
ATCCATGAGGGTAAGTTCCCTGTTGGAACTAAAGTTCAAGCTGTCTTTAGTGACGATGGCGAGTGGTATGATG
CGACCATTGAGGCACATACTGCAAATGGCTATTTTGTTGCTTATGATGAGTGGGGAAACAAGGAAGAGGTGG
ATCCAGATAATGTGAGGCCAATCGAGCAAAATGCTATTGTCGAA

SPF30.3FL **(1493 bp)**

ATGGTAGGAGGAGTAGAAGAATTGAGTATTGAACAGTTAGCTTCGAGTATCTCTACCTACAAAGAACAACTCG
AACAGGTTAGGCAGCTTTTGTCAGAGGATCCTAGGAACTCGGAATATGCAGACATGGAAAAGGAGCTTAAAG
AGGTGATCAATCCCTATTTTAATCACTTATTTTGTGTGATCAAATCTTTTCATCTTTGTTGTGGATTCGTTTTATT
AGTCTCCCTTAGTTTGAGTGAAATTCATTGATCCTTATGTCTTTAGCATTTGCGGCATACACAACATGATTAGG
TTACTTAGCATGTAGGTTTTGCATACGGTGCTCTTTATGTTTCTATTCCCTGCCTCTTTTGAAGGAATCTGCGTG
TTGTTTGAAATAGCAACATCATTTAACAGCTAGCAGATAAAGTGGAGTTTTCTTTCAGTTCTGGGGTTTGAAAG
GTGAGTATGTTTTTGAGTGGCATGTAACTCCGGCAGTGGAGTTTGGGTCAGCCAAAAGCAGTTGTTGGCGTAT
ACATAAAAGTAGGAGAGTTAGCAAAGAATTCATCATTTCTATCTTGGTTATTTGTGTTGTGCAGGTGTGCTTAT
ATGGGTCATGATGGCCAGCCTGCTTCTATCTCCTGATTTCTCGTGTTTGATCCCTGAAGTTCTACTTCTGAAAAT
CTTTACTGATATATTGTGTATAATTGCTAGAAATCCTCAATTCTCTGAAACTTTGTTTTTGCACCATATAGGTGA
TTGCATTGACAGAGGAAGTTCTTGCAACTGCAAAGCAAAATGAGATTTCTCTATCAGATGCCGGAGTTAGTGC
TGAAGCAACTCCTGGCTCACCCGATTTAGAGGGTGCTTGGGAAAAGACGGGTTTAAGGAATGACCCAATCCAT
GAGGGTAAGTTCCCTGTTGGAACTAAAGTTCAAGCTGTCTTTAGTGACGATGGCGAGTGGTATGATGCGACCA
TTGAGGCACATACTGCAAATGGCTATTTTGTTGCTTATGATGAGTGGGGAAACAAGGAAGAGGTGGATCCAGA
TAATGTGAGGCCAATCGAGCAAAATGCTATTGTCGAAGCTGAGAGATTAGCTCAAGCTACCAAAAATGCTCTC
AAAAGAAAGATTGAGAAAGCTGCGAGTTCTGATTATCAGACAAAAACTCTACCAGCAAAGCTCAAAATCGAT
CCTAATGATCCCGAGGATGTAAAAATAGCTAAGCGTAAGAAGATACATGCTTTCAAATCCAAGGCAAGGTTTG
AGCAACTCGAGGTTGTGCAGAACAAGAAACAGAATGATTGGCAACAGTTTCAGACGACTAAAGCCAAAACTA
AAAAGGTAGGGTTCTTCACAGGGAGGAAGAAAGAGAGTATATTTAAATCACCTGAGGATCCATTTGGAAAAG
TGGGTGTGACTGGAAGTGGGAAAGGTTTGACAGATTTCCAAAAGCGAGAGAAGCATCTTCATCTCAAGTCTGG
TAATGCTGAGGGCACTGATGAATGA

The coding sequence (CDS) of *SPF30.3* is shaded, with alternating dark blue and light blue showing different exons. The unshaded region is the second intron of *SPF30*. The two cassette exons within intron 2 (Figure 7A) are underlined.

Supplementary Dataset S2 Sequences of splice isoforms identified in *SPF30* intron 2 by Sanger sequencing

Sequences identified with primers F1/R1

#1
GCCGCCCTCCTGCAGTTCGAGATTATGGTAGGAGGAGTAGAAGAATTGAGTATTGAACAGTTAGCTTCGAGTA
TCTCTACCTACAAAGAACAACTCGAACAGGTTAGGCAGCTTTTGTCAGAGGATCCTAGGAACTCGGAATATGC
AGACATGGAAAAGGAGCTTAAAGAGCATGTAGGTTTTGCATACGGTGCTCTTTATGTTTCTATTCCCTGCCTCT
TTTGAAGGAATCTGCGTGTTGTTTGAAATAGCAACATCATTTAACAGCTAGCAGATAAAGTGGAGTTTTCTTTC
AGTTCTGGGGTTTGAAAGGTGTGCTTATATGGGTCAAATCTCTAGAGGATCCCCGGGTACCGAGCTCGAATTC
GTAATCATGGTCATAGCTGTTTCCTGTGTGAAATTGTTATCCGCTCACAATTCCACACAACATACGAGCCGGAA
GCATAAAGTGTAAAGCCTGGGGTGCCTAATGAGTGAGCTAACTCACATTAATTGCGTTGCGCTCACTGCCCGC
TTTCCAGTCGGGAAACCTGTCGTGCCAGCTGCATTAATGAATCGGCCAACGCGCGGGGAGAGGCGGTTTGCGT
ATTGGGCGCTCTTCCGCTTCCTCGCTCACTGACTCGCTGCGCTCGGTCGTTCGGCTGCGGCGAGCGGTATCAGC
TCACTCAAAGGCGGTAATACGGTTATCCACAGAATCAGGGGATAACGCAGGAAAGAACATGTGAGCAAAAGG
CCAGCAAAAGGCCAGGAACCGTAAAAAGGCCGCGTTGCTGGCGTTTTTCCATAGGCTCCGCCCCCCTGACGAG
CATCACAAAAATCGACGCTCAAGTCAGAGGTGGCGAAACCCGACAGGACTATAAAGATACCAGGCGTTTCCC
CCTGGAAGCTCCCTCGTGCGCTCTCCTGTTCCGACCCTGCCGCTTACCGGATACCTGTCCGCCTTTCTCCCTTCG
GGAAGCGTGCGCTTTCTCATAGCTCACGCTGTAGTATCTCAGTTCGGTGTAGTCGTTCGCTCAGCTGGGCTGTG
TGCACGACCCCCGTTCAGCCCGACGCTGCGCTTATCGTACTATCGTCTGAGTCACCCGTTAGACCGACTTATCG
CCACTGCAGCAGCCACTGTACAGATAGCAAGCGAGTTGTAGCGTGCTACAAGGTCTGATGGGGCTAACTCCGC
TACCTAGAGAAAAGATTGGATCTGCCCTCTGCTGAGCAGTTCCTCGAAAGATGTTTATCTTTGATCGGCACAC
ACCCCGTAACGGTTTTGTGTGGACATTCGCAAAGATCAGAGCTCTGTACTATCGGGTCGTACTCTACTGAGAA
CAACTGGAAGGTGTGGCGTCTGATACTGA

Sequence of the retained cassette exon 1 (141 bp) is underlined.

#2
AAAGTCGTATAGGGCGATTGATTTAGCGGCCGCGAATTCGCCCTTGTTAGGCAGCTTTTGTCAGAGGATCCTA
GGAACTCGGAATATGCAGACATGGAAAAGGAGCTTAAAGAGGTTTTGCATACGGTGCTCTTTATGTTTCTATT
CCCTGCCTCTTTTGAAGGAATCTGCGTGTTGTTTGAAATAGCAACATCATTTAACAGCTAGCAGATAAAGTGG
AGTTTTCTTTCAGTTCTGGGGTTTGAAAGGTGTGCTTATATGGGTCAAAGGGCGAATTCGTTTAAACCTGCAGG
ACTAGTACCTTTAGTGAGGGTTAATTCTGAGCTTGGCGTAATCATGGTCATAGCTGTTTCCTGTGTGAAATTGT
TATCCGCTCACAATTCCACACAACATACGAGCCGGAAGCATAAAGTGTAAAGCCTGGGGTGCCTAATGAGTG
AGCTAACTCACATTAATTGCGTTGCGCTCACTGCCCGCTTTCCAGTCGGGAAACCTGTCGTGCCAGCTGCATTA
ATGAATCGGCCAACGCGCGGGGAGAGGCGGTTTGCGTATTGGGCGCTCTTCCGCTTCCTCGCTCACTGACTCG
CTGCGCTCGGTCGTTCGGCTGCGGCGAGCGGTATCAGCTCACTCAAAGGCGGTAATACGGTTATCCACAGAAT
CAGGGGATAACGCAGGAAAGAACATGTGAGCAAAAGGCCAGCAAAAGGCCAGGAACCGTAAAAAGGCCGCG
TTGCCTGGCGTTTTTCCATAGGCTCCGCCCCCCTGACGAGCATCACAAAAATCGACGCTCAAGTTCAGAGGTG
GCGAAACCCGACAGGACTATAAAGTTACCAGGCGTTTCCCCCTGGAGCTCCCTCGTGCGCTCTCCTGTTCCGA
CCCTGCCGCTTACCGGATACCTGTCCGCTTTCCTTCCGGGAAAAGGGGGGGCTTTCTCATAGTCCGTAAGGATT
CACTCCTGGGGGGGGGGGGTCCCCCCACGCGGGGGGGGGGGGGCGAACCCCGTCAGCCGACGCTGCGCTATC
GTACTATTATTCTTGAGTCAACCGTAGAACGCTTCGCATGCAAGCATGTACGGATACTACAGAGGATGAGCCG
TGTAAGATCTCTTTCATGGGTGCTATCGTACTAGAACAATGGATCGGCTCTGACACTGAAGTGGACTGGCACC
ATCGGTGTGCATCAGTACTCGTCGACATGACGTATCTTCA

Sequence of the retained cassette exon 1 (134 bp) is underlined.

#3
GGGACGTAGCTGCAGTCGACGATTATGGTAGGAGGAGTAGAAGAATTGAGTATTGAACAGTTAGCTTCGAGT
ATCTCTTCCTACAAAGAACAACTCGAACAGGTTAGGCAGCTTTTGTCAGAGGATCCTAGGAACTCGGAATATG
CAGACATGGAAAAGGAGCTTAAAGAGGAATCTGCGTGTTGTTTGAAATAGCAACATCATTTAACAGCTAGCA
GATAAAGTGGAGTTTTCTTTCAGTTCTGGGGTTTGAAAGGTGTGCTTATATGGGTCAATCTCTAGAGGATCCCC
GGGTACCGAGCTCGAATTCGTAATCATGGTCATAGCTGTTTCCTGTGTGAAATTGTTATCCGCTCACAATTCCA
CACAACATACGAGCCGGAAGCATAAAGTGTAAAGCCTGGGGTGCCTAATGAGTGAGCTAACTCACATTAATT
GCGTTGCGCTCACTGCCCGCTTTCCAGTCGGGAAACCTGTCGTGCCAGCTGCATTAATGAATCGGCCAACGCG
CGGGGAGAGGCGGTTTGCGTATTGGGCGCTCTTCCGCTTCCTCGCTCACTGACTCGCTGCGCTCGGTCGTTCGG
CTGCGGCGAGCGGTATCAGCTCACTCAAAGGCGGTAATACGGTTATCCACAGAATCAGGGGATAACGCAGGA
AAGAACATGTGAGCAAAAGGCCAGCAAAAGGCCAGGAACCGTAAAAAGGCCGCGTTGCTGGCGTTTTTCCAT
AGGCTCCGCCCCCCTGACGAGCATCACAAAAATCGACGCTCAAGTCAGAGGTGGCGAAACCCGACAGGACTA
TAAAGATACCAGGCGTTTCCCCCTGGAAGCTCCCTCGTGCGCTCTCCTGTTCCGACCCTGCCGCTTACCGGATA
CCTGTCCGCCTTTCTCCCTTCGGGAAGCGTGGCGCTTTCTCATAGCTCACGCTGTAGGTATCTCAGTTCGGTGT
AGTCGTTCGCTCAAGCTGGGCTGTGTGCACGAACCCCCCGTTCAGCCCGACCGCTGCGCTTATCGGTACTATCGT

CTGAGTCACCCGTAGACCGACTATCGCACTGCAGCAGCACTGTACAGATAGCAGGCGAGGTATGTAGCGGTG
CTAAGAGTCTGATGGTGGCTACTACGCTCACTAGAAGAAAGAATGTATCTGCCTCGCTGACGTACTCGAAAAT
TGGACCTGGATCCGCACAACACCGTGTGACGGGTGTGTGCGCGCGAAATCCAAAGAATCGAGCACTGATCCG
GTGCGTGGGAACCAAAAGTGTGTGCGTTTACAAGAGTCATGCACACTCATATGAGT

Sequence of the retained cassette exon 1 (85 bp) is underlined.

#4
CCGTGTGGCTGCAGTCGACGATTATGGTAGGAGGAGTAGAAGAATTGAGTATTGAACAGTTAGCTTCGAGTAT
CTCTACCTACAAAGAACAACTCGAACAGGTTAGGCAGCTTTTGTCAGAGGATCCTAGGAACTCGGAATATGCA
GACATGGAAAAGGAGCTTAAAGAGCAACATCATTTAACAGCTAGCAGATAAAGTGGAGTTTTCTTTCAGTTCT
GGGGTTTGAAAGGTGTGCTTATATGGGTAAAATCTCTAGAGGATCCCCGGGTACCGAGCTCGAATTCGTAATC
ATGGTCATAGCTGTTTCCTGTGTGAAATTGTTATCCGCTCACAATTCCACACAACATACGAGCCGGAAGCATA
AAGTGTAAAGCCTGGGGTGCCTAATGAGTGAGCTAACTCACATTAATTGCGTTGCGCTCACTGCCCGCTTTCC
AGTCGGGAAACCTGTCGTGCCAGCTGCATTAATGAATCGGCCAACGCGCGGGGAGAGGCGGTTTGCGTATTG
GGCGCTCTTCCGCTTCCTCGCTCACTGACTCGCTGCGCTCGGTCGTTCGGCTGCGGCGAGCGGTATCAGCTCAC
TCAAAGGCGGTAATACGGTTATCCACAGAATCAGGGGATAACGCAGGAAAGAACATGTGAGCAAAAGGCCA
GCAAAAGGCCAGGAACCGTAAAAAGGCCGCGTTGCTGGCGTTTTTCCATAGGCTCCGCCCCCCTGACGAGCAT
CACAAAAATCGACGCTCAAGTCAGAGGTGGCGAAACCCGACAGGACTATAAAGATACCAGGCGTTTCCCCCT
GGAAGCTCCCTCGTGCGCTCTCCTGTTCCGACCCTGCCGCTTACCGGATACCTGTCCGCCTTTCTCCCTTCGGG
AAGCGTGGCGCTTTCTCATAGCTCACGCTGTAGTATCTCAGTTCGGTGTAGTCGTTCGCTCAAGCTGGGCTGTG
TGCACGAACCCCCCGTTCAGCCCGACCGCTGCGCCTTATCCGGTACTATCGTCTGAGTCAACCCGGTAAGACACG
ACTTATCGCACTGCAGCAGCACTGTACAGATAGCAGAGCGAGTATGTAGCGGTGCTACGAGTCTGATGGTGCT
ACTACGGCTACCTAGAAAACGAATTGATCTGCCCCTGCTGAGCAGTACTCGAAAAATGTAGCTTGATCGCAAC
AACACCGCTGGAACGTGTTTTTGTGCAACGCGATTCGCGAAAGATCGAACTGACTTAGGGTTGACTTGGCAAC
CCTAGAGGTGGCCGGAATACAAAGAACTTCCAGCCCTCTTAAAGAGTATC

Sequence of the retained cassette exon 1 (61 bp) is underlined.

#5
AAAATAGTATAGGGCGATTGATTTAGCGGCCGCGAATTCGCCCTTGTTAGGCAGCTTTTGTCAGAGGATCCTA
GGAACTCGGAATATGCAGACATGGAAAAGGAGCTTAAAGAGCTAGCAGATAAAGTGGAGTTTTCTTTCAGTT
CTGGGGTTTGAAAGGTGTGCTTATATGGGTCAAAGGGCGAATTCGTTTAAACCTGCAGGACTAGTACCTTTAG
TGAGGGTTAATTCTGAGCTTGGCGTAATCATGGTCATAGCTGTTTCCTGTGTGAAATTGTTATCCGCTCACAAT
TCCACACAACATACGAGCCGGAAGCATAAAGTGTAAAGCCTGGGGTGCCTAATGAGTGAGCTAACTCACATT
AATTGCGTTGCGCTCACTGCCCGCTTTCCAGTCGGGAAACCTGTCGTGCCAGCTGCATTAATGAATCGGCCAA
CGCGCGGGGAGAGGCGGTTTGCGTATTGGGCGCTCTTCCGCTTCCTCGCTCACTGACTCGCTGCGCTCGGTCGT
TCGGCTGCGGCGAGCGGTATCAGCTCACTCAAAGGCGGTAATACGGTTATCCACAGAATCAGGGGATAACGC
AGGAAAGAACATGTGAGCAAAAGGCCAGCAAAAGGCCAGGAACCGTAAAAAGGCCGCGTTGCTGGCGTTTTT
CCATAGGCTCCGCCCCCCTGACGAGCATCACAAAAATCGACGCTCAAGTCAGAGGTGGCGAAACCCGACAGG
ACTATAAAGATACCAGGCGTTTCCCCCCTGGAAGCTCCCTCGTGCGCTCTCCTGTTCCGACCCTGCCGCTTACC
GGATACCTGTCCGCCTTTCTCCCTTCGGGGAAGCGTGGCGCTTTCTCATAGCTCACGCTGAGGGTATCTCAGTT
CGGGTGTAGGTCGTTCGCTCCAGCTGGGGCTGTGTGCACGAACCCCCCGTTCAGCCCGACCGCTGCGCCTTAT
CCGGTAACTATCGTCTTGAGTCCACCCGGTAAGACACGACTTATCGCACTGGCAGCAGCACTGTTACAGATAG
CAAGCGAGATGTAGCGTGCTACAATTCTTTTAGATGGTGGCTACTACGCTACTAAAGAACAGAATTGGTTCTG
CTTGCTGAGCAGTACTCGTACAGATAGTAGTTGATCGTAACCCGACACACGGGTTGTGTGGTCGATCGCGAAG
ATCAGGACTGACTTACGTGACTGGCACCACCGATGCGAAACTGTTAGCCGAGTATCGCTACATGATTCTGCAC
TGTA

Sequence of the retained cassette exon 1 (45 bp) is underlined.

Sequence identified with primers F1/R3

#A
TCGCTCACGACAGGAGGGCGCGGATTCAATACTGTTTCTTATGTGAGGCGTATGTAGGCACATCAAGAGCTGA
GCCGCTTCAATCTGGTGGTATCGTACATGGCTGTTGCATGCGATAGTCGGTTTACGGTGGATCAAGCGATAGT
ACGATAAGCCAGCGGTCGGCTGACGGGGGTCGTGCACACAGCCCAGCTGGAGCGACGACTACACGACTGAGA
AACTACAGCGTGAGCTATGAGAAAGCGCACGCTTCCGAAGGAGAAAGCGGACAGGTATCCGGTAAGCGGCAG
GGTCGGAACAGAGAGCGCACGAGGGAGCTTCCAGGGGAAACGCCTGGTATCTTTATAGTCCTGTCGGGTTTCG
CCACCTCTGACTTGAGCGTCGATTTTTGTGATGCTCGTCAGGGGGGCGGAGCGTATGGAAAAACGCCAGCAAC
GCGGCTTTTTACGGTTCCTGGCCTTTTGCTGGCCTTTTGCTCACATGTTCTTTCCTGCGTTATCCCCTGATTCTGT
GGATAACCGTATTACCGCCTTTGAGTGAGCTGATACCGCTCGCCGCAGCCGAACGACCGAGCGCAGCGAGTCA
GTGAGCGAGGAAGCGGAAGAGCGCCCAATACGCAAACCGCCTCTCCCCGCGCGTTGGCCGATTCATTAATGC
AGCTGGCACGACAGGTTTCCCGACTGGAAAGCGGGCAGTGAGCGCAACGCAATTAATGTGAGTTAGCTCACT

CATTAGGCACCCCAGGCTTTACACTTTATGCTTCCGGCTCGTATGTTGTGTGGAATTGTGAGCGGATAACAATT
TCACACAGGAAACAGCTATGACCATGATTACGCCAAGCTCAGAATTAACCCTCACTAAAGGTACTAGTCCTGC
AGGTTTAAACGAATTCGCCCTTGTTAGGCAGCTTTTGTCAGAGGATCCTAGGAACTCGGAATATGCAGACATG
GAAAAGGAGCTTAAAGAGGAATCTGCGTGTTGTTTGAAATAGCAACATCATTTAACAGCTAGCAGATAAAGT
GGAGTTTTCTTTCAGTTCTGGGGTTTGAAAG**GTGTGCTTATATGGGTCATGATGGCCAGCCTGCTTCTATC**
TCCTGATTTCTCGTGTTTGATCCCTGAAGTGATTGCATTGACAGAGGAAGTTCTTGCAACTGCAAAGCAAAA
TGAGATTTCTCTATCAGATGCCGGAGTTAGTGCTGAAGCAACTCCCGGCTCACCCGATTTAGAGGGTGCTTGG
AAGGGCGAATTCGCGGCCGCTAAAGCAATCGCCCTATAGAAATCGTACCG

Sequence of the retained cassette exon 1 (85 bp) is underlined; sequence of the retained cassette exon 2 is in bold.

#B

CACAAAAAAAAAACCCGGCTCACACGCGTTGTTGTTGGCGGATCAAGATACCAACTCTTTCGAAGTACGATCA
GCAGAGGCAGATACCAATCTGTTCTTTAGGTAGCGTAGTAGCACCACTCCAAGAACTCGTAGCACGCTACATC
TCGCCTGCTATCCTGTTACCAGTGGCTGCTGCCAGTGGCGATAGTCGTGTCTTACCGGGTGGACTCAGACGATA
GTACCGATAAGCGCAGCGGTCGGCTGAACGGGGGTTCGTGCACACAGCCCAGCTTGAGCGAACGACTACACC
GAACTGAGATACCTACAGCGTGAGCTATGAGAAAGCGCCACGCTTCCCGAAGGGAGAAAGGCGGACAGGTAT
CCGGTAAGCGGCAGGGTCGGAACAGGAGAGCGCACGAGGGAGCTTCCAGGGGGAAACGCCTGGTATCTTTAT
AGTCCTGTCGGGTTTCGCCACCTCTGACTTGAGCGTCGATTTTTGTGATGCTCGTCAGGGGGGCGGAGCCTATG
GAAAAACGCCAGCAACGCGGCCTTTTTACGGTTCCTGGCCTTTTGCTGGCCTTTTGCTCACATGTTCTTTCCTGC
GTTATCCCCTGATTCTGTGGATAACCGTATTACCGCCTTTGAGTGAGCTGATACCGCTCGCCGCAGCCGAACGA
CCGAGCGCAGCGAGTCAGTGAGCGAGGAAGCGGAAGAGCGCCCAATACGCAAACCGCCTCTCCCCGCGCGTT
GGCCGATTCATTAATGCAGCTGGCACGACAGGTTTCCCGACTGGAAAGCGGGCAGTGAGCGCAACGCAATTA
ATGTGAGTTAGCTCACTCATTAGGCACCCCAGGCTTTACACTTTATGCTTCCGGCTCGTATGTTGTGTGGAATT
GTGAGCGGATAACAATTTCACACAGGAAACAGCTATGACCATGATTACGCCAAGCTCAGAATTAACCCTCACT
AAAGGTACTAGTCCTGCAGGTTTAAACGAATTCGCCCTTGTTAGGCAGCTTTTGTCAGAGGATCCTAGGAACT
CGGAATATGCAGACATGGAAAAGGAGCTTAAAGAG**GTGTGCTTATATGGGTCATGATGGCCAGCCTGCTT**
CTATCTCCTGATTTCTCGTGTTTGATCCCTGAAGTGATTGCATTGACAGAGGAAGTTCTTGCAACTGCAAAG
CAAAATGAGATTTCTCTATCAGATGCCGGAGTTAGTGCTGAAGCAACTCCTGGCTCACCCGATTTAGAGGGTG
CTTGGAAGGGCGAATTCGCGGCCGCTAAATCAATCGCCCTATAGTAATCATTGGGTT

Cassette exon 1 is skipped here; sequence of the retained cassette exon 2 is in bold.

Sequence identified with primers *SPF30.3FL*-GPF-F/R

#C
AGGGTAATTACTATAGGGCGATTGATTTAGCGGCCGCGAATTCGCCCTTCGCGCCAAGCTATCAAACAAGTTT
GTACAAAAAAGCAGGCTCCGCGGCCGCCCCCTTCACCATGGTAGGAGGAGTAGAAGAATTGAGTATTGAACA
GTTAGCTTCGAGTATCTCTACCTACAAAGAACAACTCGAACAGGTTAGGCAGCTTTTGTCAGAGGATCCTAGG
AACTCGGAATATGCAGACATGGAAAAGGAGCTTAAAGAGGTGTGCTTATATGGGTCATGATGGCCAGCCTGC
TTCTATCTCCTGATTTCTCGTGTTTGATCCCTGAAGTTCTACTTCTGAAAATCTTTACTGATATATTGTGTATAA
TTGCTAGAAATCCTCAATTCTCTGAAACTTTGTTTTTGCACCATATAGGTGATTGCATTGACAGAGGAAGTTCT
TGCAACTGCAAAGCAAAATGAGATTTCTCTATCAGATGCCGGAGTTAGTGCTGAAGCAACTCCTGGCTCACCC
GATTTAGAGGGTGCTTGGAAGGGCGAATTCGTTTAAACCTGCAGGACTAGTACCTTTAGTGAGGGTTAATTCT
GAGCTTGGCGTAATCATGGTCATAGCTGTTTCCTGTGTGAAATTGTTATCCGCTCACAATTCCACACAACATAC
GAGCCGGAAGCATAAAGTGTAAAGCCTGGGGTGCCTAATGAGTGAGCTAACTCACATTAATTGCGTTGCGCTC
ACTGCCCGCTTTCCAGTCGGGAAACCTGTCGTGCCAGCTGCATTAATGAATCGGCCAACGCGCGGGGAGAGGC
GGTTTGCGTATTGGGCGCTCTTCCGCTTCCTCGCTCACTGACTCGCTGCGCTCGGTCGTTCGGCTGCGGCGAGC
GGTATCAGCTCACTCAAAGGCGGTAATACGGTTATCCACAGAATCACGGGATAACGCATGAAAGAACATGTG
AGCAAAAGGCCAGCAAAAGGCCAGGAACCGTAAAAAGCCGCGTTTGCTGGCGTTTTCCATAGGCTCCGCCCC
CCTGACGAGCATCACCAAAATCGACGCTCAAGTCAAAGGTGGCGAAACCCGACAGGACTTATAAGAATACCA
GGCGTTTCCCCTGGAGCTCCCTCGTGCGCCTTCTGTTCCGACCTGGCGCTTACCGGATACCTGTCGCCTTCTCCC
CTCGGGAAGAGGGGCGCTTTTCATGCTCCGCTGTAGAATACAGA

The retained sequence in *SPF30* intron 2 is underlined.
