# Supplementary figures and images for: The role of Arabidopsis Splicing Factor 30 in floral transition and the implications of its alternative splicing
Source: Plant Physiol. 2025 Jul 31;198(4):kiaf335. doi: 10.1093/plphys/kiaf335 (PMC12393150; doi:10.1093/plphys/kiaf335)

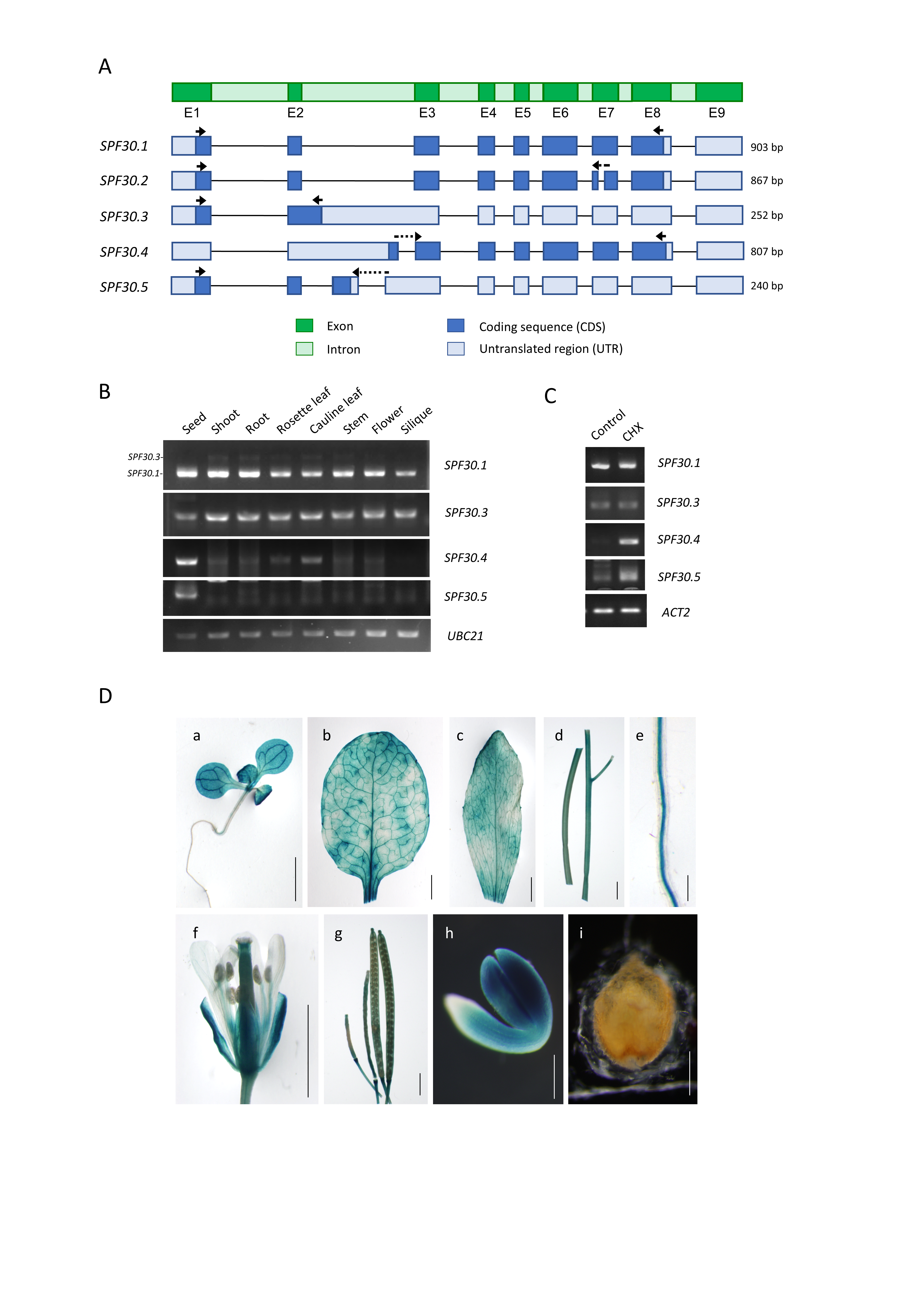

Supplement: kiaf335_Supplementary_Data [file kiaf335_supplementary_data.zip › SPF30 Figure 1.TIF]

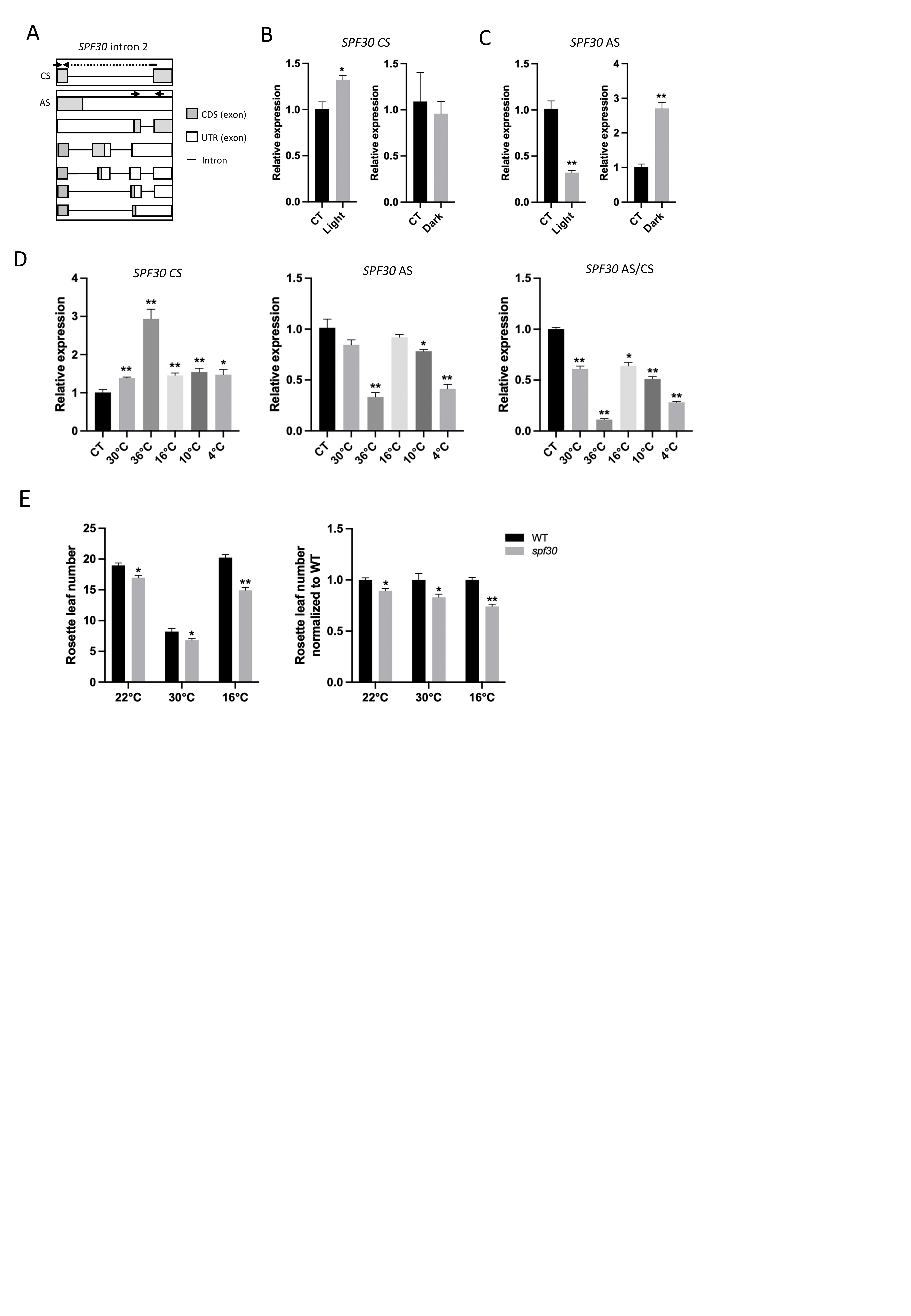

Supplement: kiaf335_Supplementary_Data [file kiaf335_supplementary_data.zip › SPF30 Figure 10.TIF]

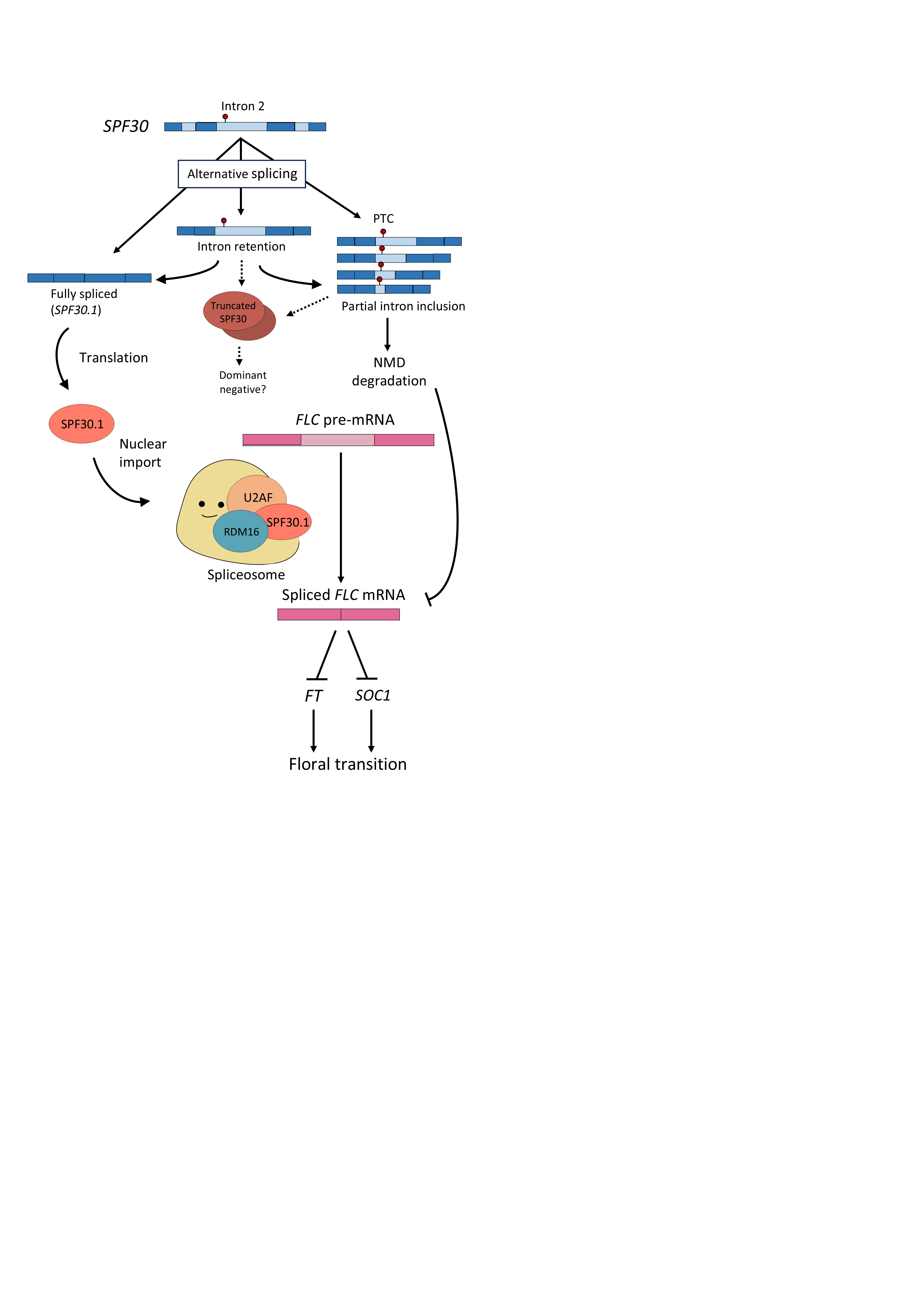

Supplement: kiaf335_Supplementary_Data [file kiaf335_supplementary_data.zip › SPF30 Figure 11.TIF]

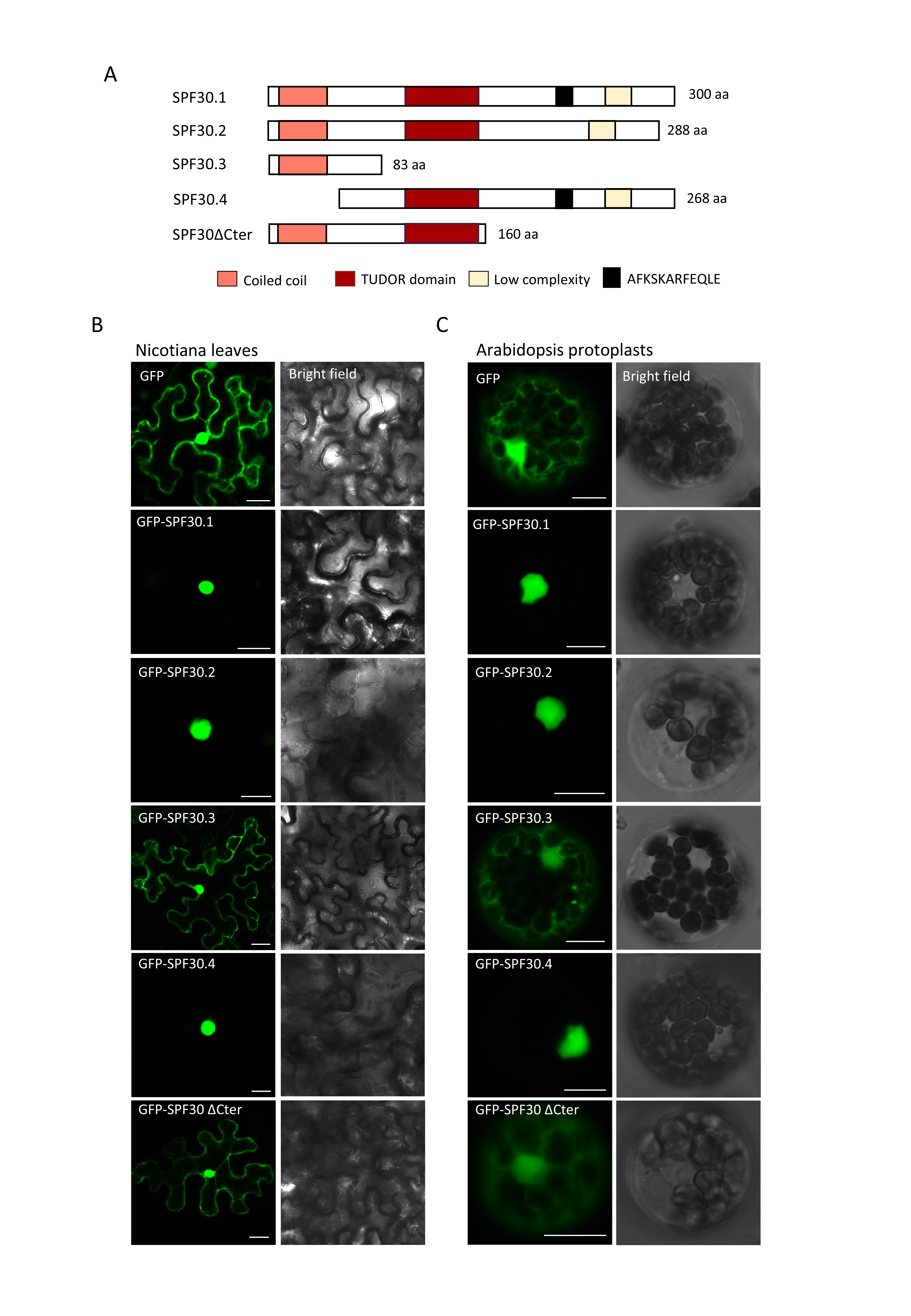

Supplement: kiaf335_Supplementary_Data [file kiaf335_supplementary_data.zip › SPF30 Figure 2.tif]

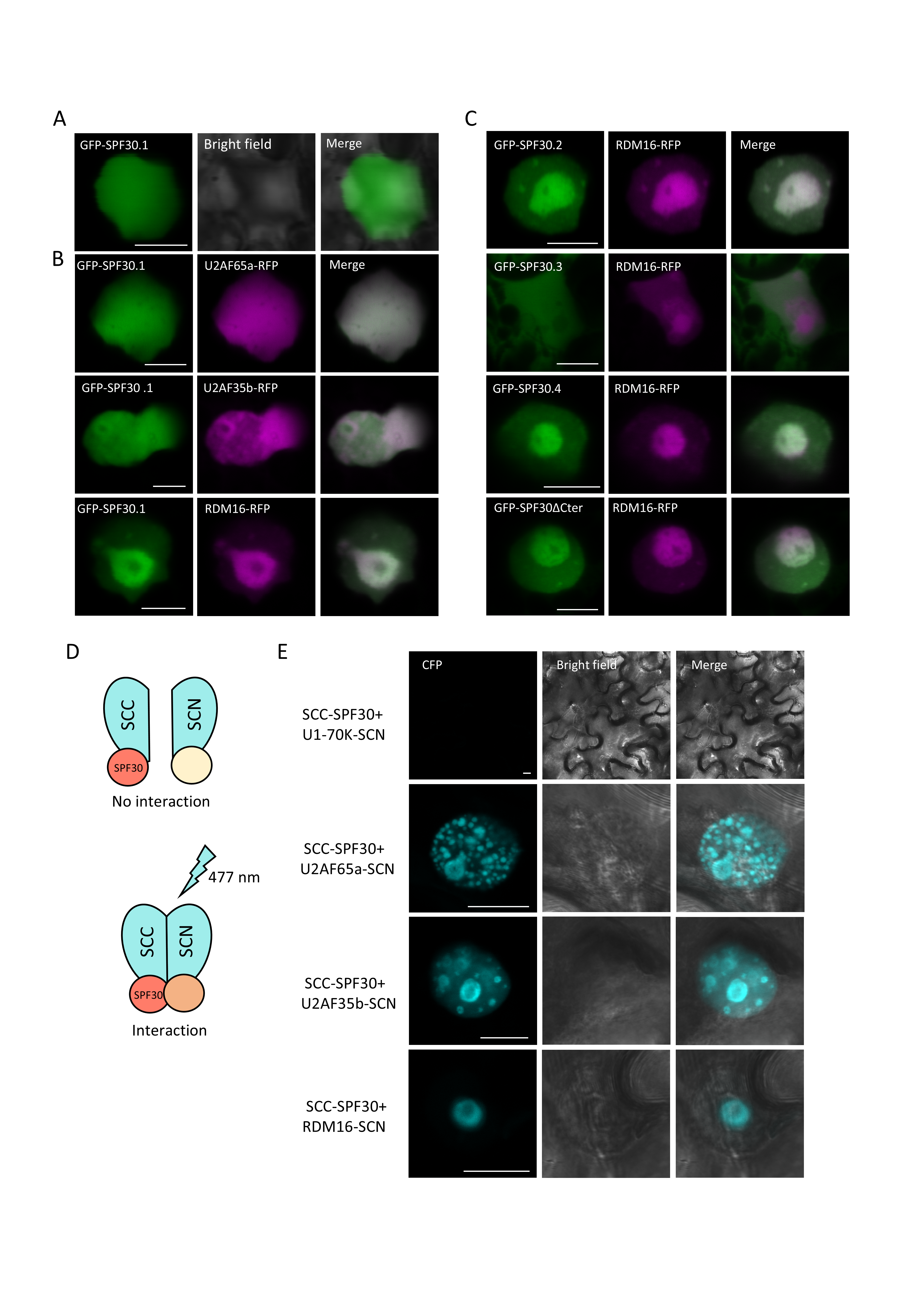

Supplement: kiaf335_Supplementary_Data [file kiaf335_supplementary_data.zip › SPF30 Figure 3.TIF]

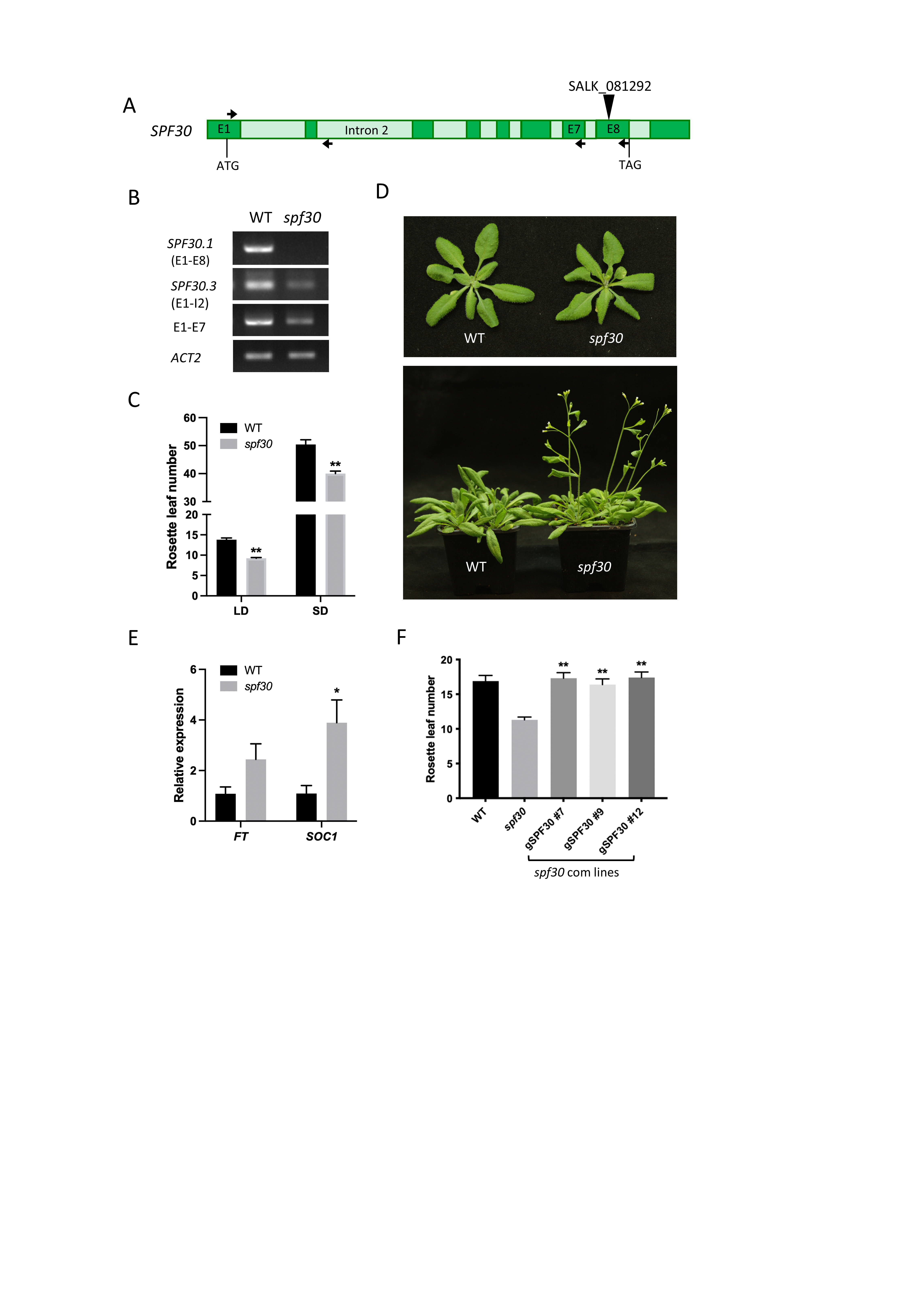

Supplement: kiaf335_Supplementary_Data [file kiaf335_supplementary_data.zip › SPF30 Figure 4.TIF]

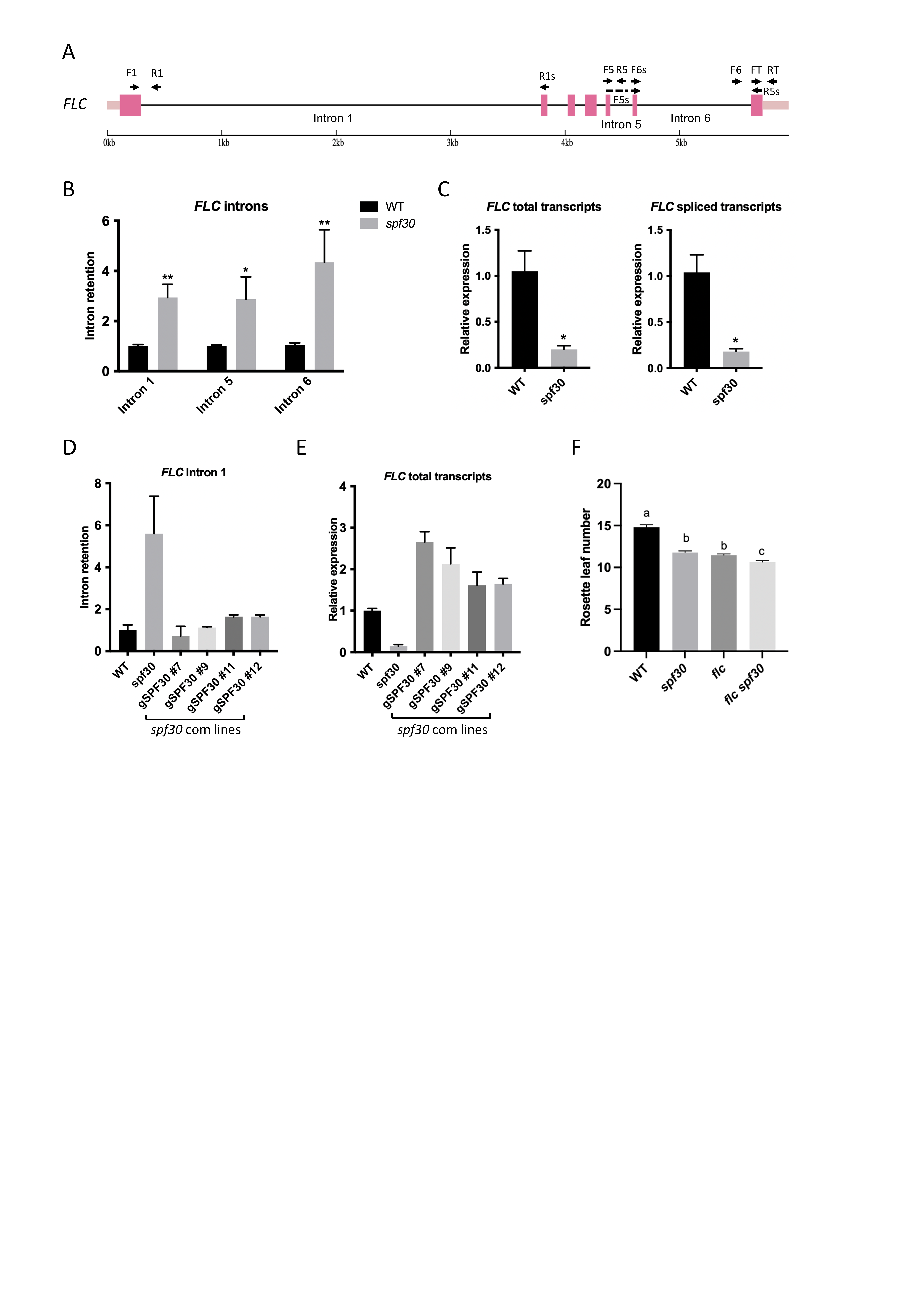

Supplement: kiaf335_Supplementary_Data [file kiaf335_supplementary_data.zip › SPF30 Figure 5.tif]

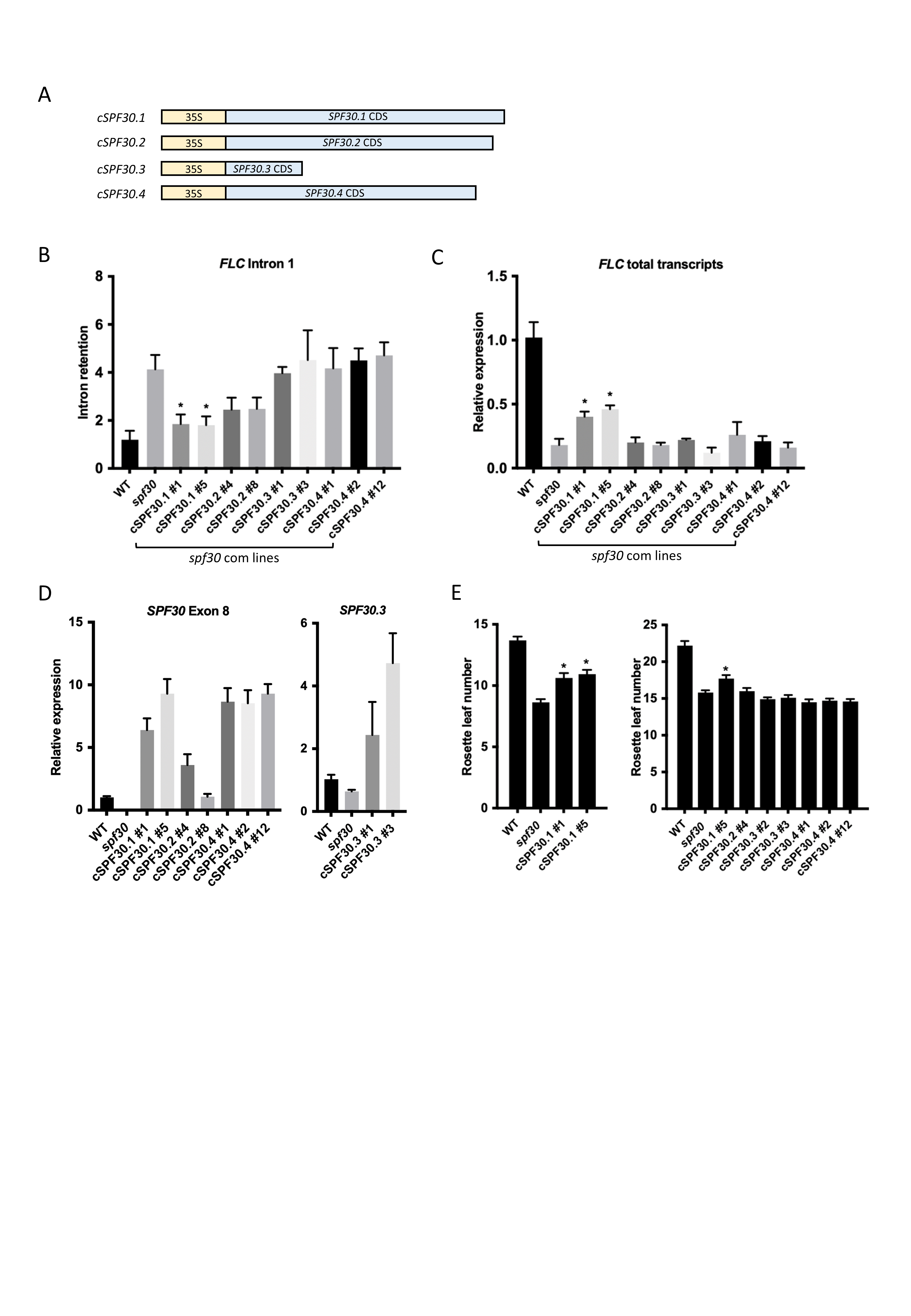

Supplement: kiaf335_Supplementary_Data [file kiaf335_supplementary_data.zip › SPF30 Figure 6.TIF]

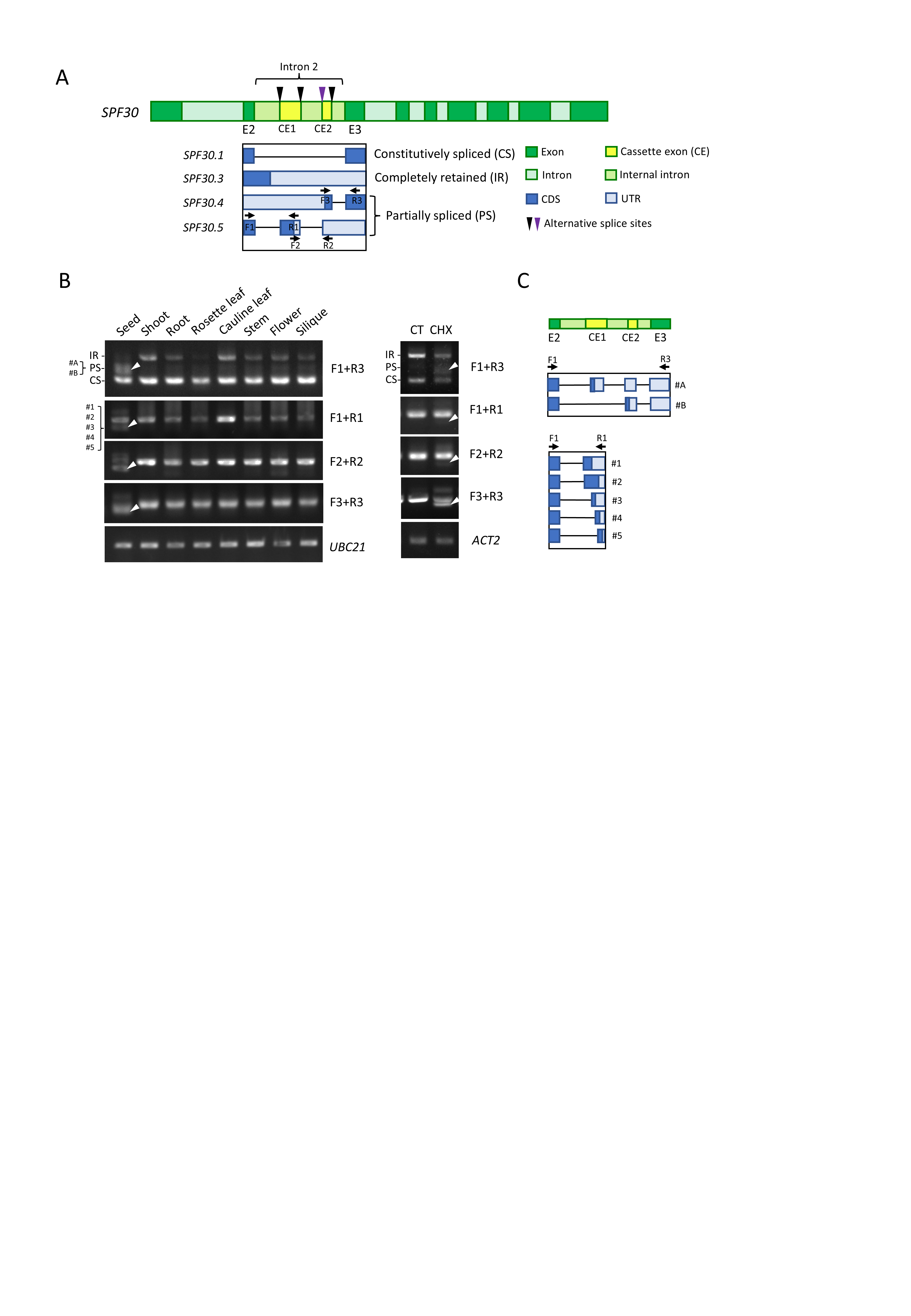

Supplement: kiaf335_Supplementary_Data [file kiaf335_supplementary_data.zip › SPF30 Figure 7.TIF]

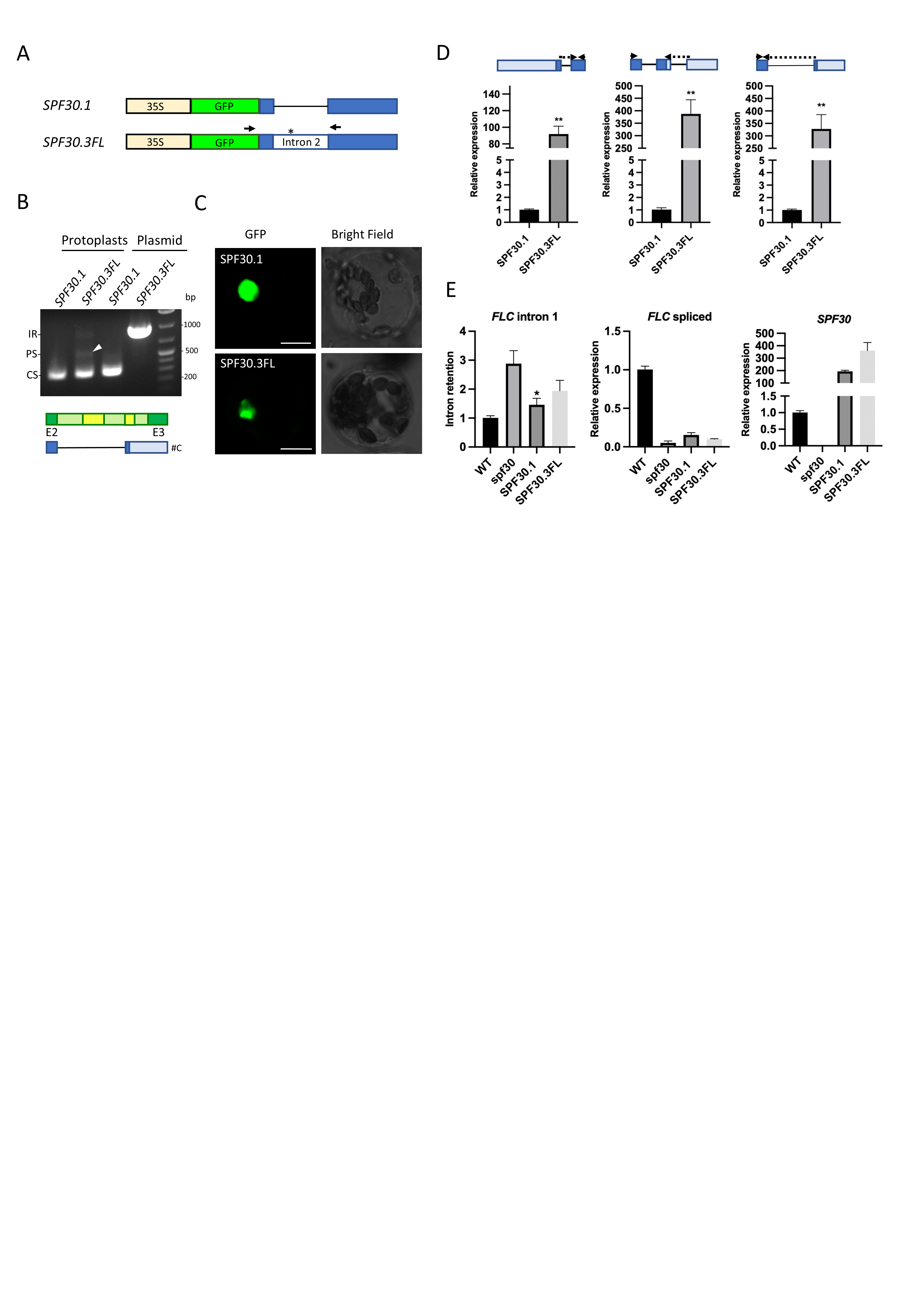

Supplement: kiaf335_Supplementary_Data [file kiaf335_supplementary_data.zip › SPF30 Figure 8.TIF]

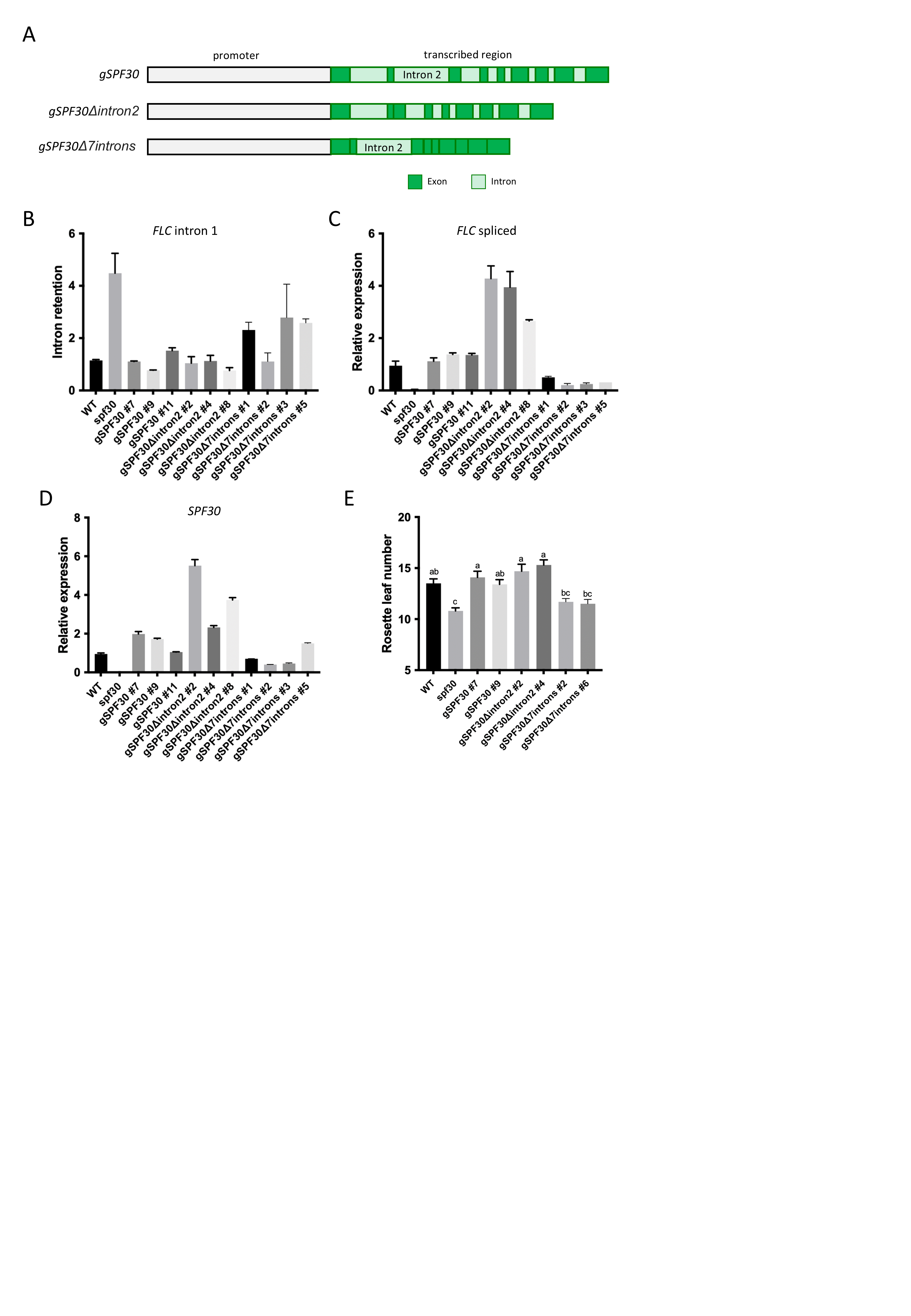

Supplement: kiaf335_Supplementary_Data [file kiaf335_supplementary_data.zip › SPF30 Figure 9.TIF]
